# High-Throughput Sequencing of Small RNAs for Diagnostics of Grapevine Viruses and Viroids in Russia

**DOI:** 10.3390/v13122432

**Published:** 2021-12-03

**Authors:** Emiliya Navrotskaya, Elena Porotikova, Eugeniya Yurchenko, Zsuzsanna Nagyne Galbacs, Eva Varallyay, Svetlana Vinogradova

**Affiliations:** 1 Institute of Bioengineering, Research Center of Biotechnology of the Russian Academy of Sciences, Leninsky Prospect 33, 119071 Moscow, Russia; emiliya.navrotskaya@mail.ru (E.N.); plantvirus@mail.ru (E.P.); 2 Federal State Budgetary Scientific Institution ‘North Caucasian Federal Scientific Horticulture and Viticulture Center’, Protection and Plant Biotechnology Scientific Center, Head, 40 Years of Victory Street 39, 350072 Krasnodar, Russia; yug.agroekos@yandex.ru; 3 Genomics Research Group, Department of Plant Pathology, Institute of Plant Protection, Hungarian University of Agriculture and Life Sciences, Szent-Gyorgyi Albert Street 4, H-2100 Godollo, Hungary; Nagyne.Galbacs.Zsuzsanna@uni-mate.hu (Z.N.G.); Varallyay.Eva@uni-mate.hu (E.V.)

**Keywords:** high-throughput sequencing (HTS), grapevine, virus, viroid, virome, small RNA (sRNA)

## Abstract

The use of high-throughput sequencing (HTS) technology has led to significant progress in the identification of many viruses and their genetic variants. In this study, we used the HTS platform to sequence small RNAs (sRNAs) of grapevine to study the virome. Isolation of RNA was performed using symptomatic grapevines collected from commercial vineyards in Krasnodar Krai in 2017–2018. To determine the viromes of vineyards, we used an integrated approach that included a bioinformatic analysis of the results of sRNA HTS and the molecular method RT-PCR, which made it possible to identify 13 viruses and 4 viroids. Grapevine leafroll-associated virus 4 (GLRaV-4), Grapevine Syrah Virus-1 (GSyV-1), Raspberry bushy dwarf virus (RBDV), Australian grapevine viroid (AGVd), and Grapevine yellow speckle viroid 2 (GYSVd-2) were identified for the first time in Russia. Out of 38 samples analyzed, 37 had mixed infections with 4–11 viruses, indicating a high viral load. Analysis of the obtained sequences of fragments of virus genomes made it possible to identify recombination events in GLRaV-1, GLRaV-2, GLRaV-3, GLRaV-4, GVT, GPGV, GRSPaV, GVA, and GFLV. The obtained results indicate a wide spread of the viruses and a high genetic diversity in the vineyards of Krasnodar Krai and emphasize the urgent need to develop and implement long-term strategies for the control of viral grapevine diseases.

## 1. Introduction

The viticulture and viniculture industries are important economic constituents in many countries [1]. Due to a significant decrease in productivity, grapevine diseases caused by viral pathogens are major obstacles to the production of grapes and wine [2,3].

Grapevines are the host for the largest number of viruses among cultivated species [4]. More than 85 grapevine-infecting viruses are currently known [5]. About 30 of them are caused by the 4 disease complexes [3]. Sixteen nepoviruses are associated with infectious degeneration and decline and characterized by infectious malformations, yellow mosaic, vein banding, stunting and decline [6]. Five viruses are associated with grapevine leafroll disease and cause the reddening or yellowing of leaves with veins remaining green and the down-rolling of leaves [7,8,9]. Six viruses among them are vitiviruses and Grapevine rupestris stem pitting-associated virus (GRSPaV), which affect the woody cylinder, cambium tissues, bark and associated with rugose wood complex [10]. Four viruses of the fleck complex cause clearing of the small veins, with the appearance of translucent spots, wrinkling, twisting and curling upward of the leaves [3].

Traditional diagnostics methods based on the use of immunological (ELISA and lateral flow immunoassay) or molecular methods (PCR, reverse transcription PCR (RT-PCR), real-time RT-PCR, nested PCR, DNA microarray, and loop-mediated isothermal amplification) only allow the detection of viruses if information about the genome of the virus or antibodies against it is available. In contrast, high-throughput sequencing (HTS) technology provides a unique opportunity to detect the presence of both previously described and unknown viruses or viroids in the sample [11,12,13].

Despite the broad capabilities of high-throughput sequencing, there is currently no universal protocol for detecting viruses and obtaining complete information about the nucleotide sequence of any viral genome. The choice of protocol depends on the type of plant, the tissue under study, the virus to be detected, and the experience of the scientist [14,15].

One of the most commonly used methods for the detection of grapevine viruses is sRNA sequencing. It is believed that by using sRNA HTS, it is difficult to detect viruses that are constantly present in a plant, and it is not always possible to assemble complete genomes of new viruses [15]. However, despite existing difficulties, this approach has made it possible to establish sequences of five new viruses, including Grapevine Pinot Gris virus [16], Grapevine Roditis leaf-discoloration-associated virus [17], Grapevine vein clearing virus [18] and others [19,20], and examine the viromes of vineyards [16,21,22,23].

The aim of this study was to investigate the viromes of vineyards and to study the distribution of viruses in vineyards in Krasnodar Krai, using the sRNA high-throughput sequencing method.

In the study, to obtain the most complete description of the viromes, we combined a bioinformatics analysis of the HTS results with the molecular diagnostics method. This allowed us to identify Australian Grapevine Viroid (AGVd), Grapevine leafroll-associated virus 4 (GLRaV-4), Grapevine Syrah virus 1 (GSyV-1), Grapevine yellow speckle viroid 2 (GYSVd-2), and Raspberry bushy dwarf virus (RBDV) for the first time in Russia. We were also able to detect the following viruses that were previously identified in Russia: Grapevine fanleaf virus (GFLV), Grapevine fleck virus (GFkV), Grapevine leafroll-associated virus 1, 2 and 3 (GLRaV-1, -2, -3), Grapevine Pinot gris virus (GPGV), Grapevine rupestris stem pitting-associated virus (GRSPaV), Grapevine rupestris vein feathering virus (GRVFV), Grapevine virus A (GVA), and Grapevine virus T (GVT). In addition, the following viroids were detected: Grapevine yellow speckle viroid (GYSVd-1) and Hop stunt viroid (HSVd).

## 2. Materials and Methods

### 2.1. Plant Sources and Preparation of sRNA Sequencing Libraries

1. Phytosanitary monitoring of commercial vineyards in Krasnodar Krai was carried out from July 2017 to September 2018 The exact coordinates of the vineyard are available in Google Earth (https://earth.google.com/earth/d/1KymmhGwAdv4Wns6VikJ2W_R736-UeR-U?usp=sharing) (accessed on 30 November 2021). For the analysis, samples were collected from grapevine plants with symptoms of viral disease (Supplementary Table S1, Supplementary Photographic Materials). To isolate total RNA, we used 1 g of vine and leaves from each of the 38 plants in accordance with the protocol described by Morante-Carriel [24]. RNA weighing 2–5 μg was extracted from 7–9 plants and combined into one pool, and sRNA sequencing libraries were prepared using the TruSeq Small RNA Library Preparation Kit (Illumina, San Diego, CA, USA) in accordance with the manufacturer’s protocol with modifications following Czotter et al. (2018) [21]. The resulting five libraries were sequenced using a HiScanSQ System (Illumina, USA) at a read length of 1 × 50 bp. Fastq files of the sequenced libraries were deposed in the SRA and can be accessed at accession number PRJNA771403.

### 2.2. Bioinformatics Analysis of HTS Results

**Figure 1:**
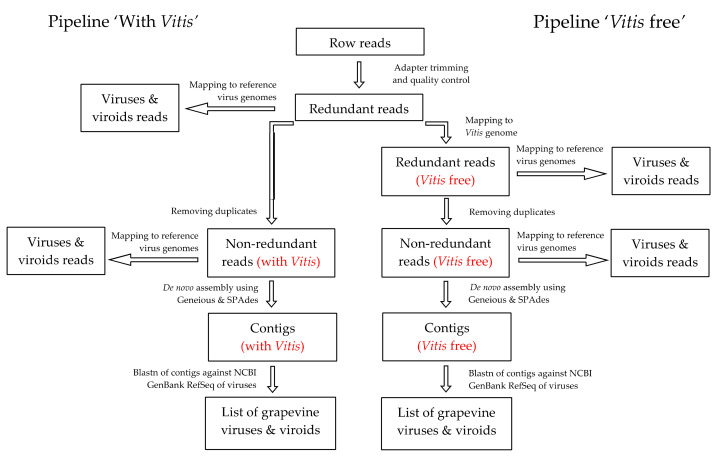
Schematic representation of the bioinformatics pipelines used for virus and viroid detection in the HTS in sRNA libraries.

The sequencing reads were processed using the Geneious Prime v. 2020.0.4 software package (Biomatters, Ltd., Auckland, New Zealand) [25] according to the scheme presented in Figure 1.

We used two bioinformatics pipelines named ‘with *Vitis*’ and ‘*Vitis* free’. According to both pipelines, the reads were trimmed using the BBDuk tool (minimum length 10 bp, minimum quality 20), and quality control was carried out. Then, for the pipeline ‘*Vitis* free’, the reads were mapped to the grapevine reference genome (GCF_000003745), and unmapped reads were used for the removal of duplicated reads to obtain unique (non-redundant) reads and their de novo assembly. The step of mapping to the grapevine reference genome was avoided in the pipeline ‘with *Vitis*’, and redundant reads were directly used for the removal of duplicated reads and de novo assembly. For de novo assembly of non-redundant reads according to both pipelines, we used the Geneious and SPAdes assemblers and tested different kmers (−15, −17, −19). The resulting contigs were analyzed using blastn against the NCBI GenBank RefSeq of viruses and viroids (release date 31 January 2021). For further analysis, we selected contigs of plant viruses with E-Values below −5 to obtain the list of viruses and viroids found in the sRNA libraries. These contigs were also analyzed using the blastn tool in NCBI.

To assess the number of reads corresponding to each virus, we mapped redundant and non-redundant reads of both pipelines to the reference sequences of detected viruses with different sensitivity levels: low and medium–low sensitivity with default program parameters as well as custom sensitivity. The parameters of the custom sensitivity were as follows: gaps maximum per read—10%, maximum gap size—3, word length—18, ignore words repeated more than 12 times, maximum mismatches per read—10%, index word length—13, and maximum ambiguity—4. The coverage of the genomes of the detected viruses was calculated as a percentage after mapping the obtained reads to the corresponding reference genomes.

### 2.3. Data Validation by RT-PCR

Validation of the presence of each virus detected in silico was initially performed by RT-PCR on all pools of total RNA used for the sRNA HTS library preparation. Only viruses and viroids for which at least one contig with an E-Value below −5 was found were validated. If a virus was detected in the pool, we carried out PCR analysis on each total RNA sample of the corresponding library. Reverse transcription was performed using 1 μg of total RNA, Random Hexamer primers, and the RevertAid H Minus Reverse Transcriptase (EP0452, Thermo Fisher Scientific, Waltham, MA, USA) in accordance with the manufacturer’s protocol.

One microliter of cDNA was used to perform PCR for the 18S rRNA gene for quality control and then to detect viruses using Taq DNA Polymerase (EP042, Thermo Fisher Scientific, USA) and previously published primers as well as primers designed on the basis of HTS reads (Supplementary Table S2). The visualization of PCR results was carried out by electrophoresis in 1.2% agarose gel stained with ethidium bromide. One amplicon was sequenced for each virus from two primers using the Sanger method using the Big Dye Terminator v.3.1 Cycle Sequencing Kit on an ABI PRIZM 3730 DNA Analyzer (Thermo Fisher Scientific, USA) in accordance with the manufacturer’s instructions.

Sequencing results were analyzed using Finch TV 1.4.0 software [26]. After validation, the assembled sequences were submitted to GenBank (www.ncbi.nlm.nih.gov/genbank/) (accessed on 30 November 2021) [27]. Additionally, we determined the Pairwise Identity (%) by comparing the validated sequences with each other, with the reference genomes (using the Geneious Prime), and with the closest genomes (according to the results of the blastn analysis) (in NCBI).

### 2.4. Phylogenetic Analysis

To carry out the phylogenetic analysis, we amplified a fragment of the RNA2 polyprotein gene for GFLV and GRVFV, a fragment of the *hsp70* gene for GLRaV-4, and fragments of coat protein genes for GLRaV-1, GLRaV-2, GLRaV-3, GFkV, GVT, GPGV, GRSPaV, GVA, GSyV-1, and RBDV. From each vineyard, we considered one sequenced isolate for this analysis. PCR was performed with 1 μL of cDNA using Phusion Hot Start II High-Fidelity DNA Polymerase (F530L, Thermo Fisher Scientific, USA) and previously published primers or those designed on the basis of HTS reads (Supplementary Table S3). The real-time PCR thermal conditions for all genes were 95 °C for 10 min, followed by 40 cycles of 95 °C for 10 s, and 55 °C (signal collection temperature) for 1 min. The amplification conditions were 98 °C for 30 s, followed by 40 cycles of 98 °C for 10 s, 56 °C for 30 s, 72 °C for 30 s per 1 kb, and a final elongation step of 72 °C for 10 min. The amplicons were sequenced bidirectionally using the Sanger method, and the nucleotide sequence was assembled using the Finch TV 1.4.0 program and submitted to GenBank (www.ncbi.nlm.nih.gov/genbank/) (accessed on 30 November 2021) [27] (Supplementary Table S4). Moreover, to determine the GLRaV-2 group, we used in silico RFLP analysis [28,29]. Multiple sequence alignment was performed using the Geneious Alignment tool of the Geneious Prime program. To determine the pairwise identity (%), the resulting sequences were compared with each other, with the reference genome, and with the closest genome. Phylogenetic trees were constructed using the maximum likelihood (ML) method with 1000 bootstrap replicates [30] in the RDP v4.10 program [31].

### 2.5. Recombination Analysis

The nucleotide sequences used for the phylogeny study of the detected viruses and others taken from GenBank were analyzed for the presence of recombinations, using the RDP v4.10 program with default program parameters. This program includes a set of eight recombination detection tools [31]. Recombination sites identified by three or more of the eight tools were considered ‘significant and clear recombination events’ [32]. Sites detected by two or fewer methods were considered ‘tentative recombination events’.

## 3. Results and Discussion

### 3.1. Phytosanitary Monitoring

**Figure 2:**
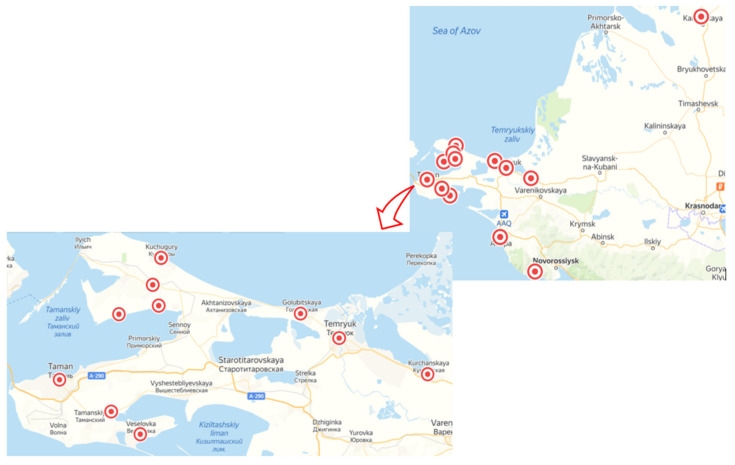
Locations of selected vineyards in Krasnodar Krai in Russia.

Phytosanitary monitoring of 12 commercial vineyards planted with 18 cultivars was carried out in Krasnodar Krai from July 2017 to September 2018 (Figure 2).

Among the selected vines, cultivars of Russian and foreign selection that are widespread worldwide were identified. Out of the 38 selected plants, 14 samples showed leafroll symptoms, four showed mosaic symptoms, 14 showed fanleaf symptoms, and 14 showed reddening of varying extents. We also observed shoot deformation, points along the veins and over the entire surface of the leaf, circles on the leaves, overgrowth, and diminishment of absence of leaves (Supplementary Table S1). Photographs of the selected plants are presented in the Supplementary Photographic Materials.

### 3.2. Results of the sRNA HTS Bioinformatics Analysis

As a result of sequencing of the sRNA libraries, we obtained 14.8–20.6 million reads for each library. After trimming the data, 14.3–20 million reads remained in the pipeline ‘with *Vitis’* and 8.3–16 million redundant reads in the pipeline *‘Vitis* free’. The numbers of non-redundant reads were much lower, 1.8–2.8 million and 1.8–2.8 million reads, respectively (Supplementary Table S5).

After assembling unique reads using the SPAdes assembler, we obtained 156–1128 contigs with N50 from 149 until 227 bp. At the same time, the Geneious assembler allowed us to assemble a larger number of contigs: 136,580–259,103 but of smaller length (N50 = 56–57 bp). Contig assembly using SPAdes and Geneious with kmer15, −17, and −19 showed that, in most cases, assembling with kmer15 allowed more virus contigs to be detected than assembling with kmer17 and kmer19 (Supplementary Table S6).

The use of two assemblers for bioinformatics data processing in Geneious allowed us to detect contigs of grapevine viruses for the blastn analysis stage. For instance, the contigs of the GRVFV (library code Zs85-SV2_S19, Lib-04_S21), GLRaV-4 (Lib-03_S20), RSPaV (Lib-03_S20), GVA (Lib-05_S22, Lib-06_S23) GSyV-1 (Zs85-SV2_S19, Lib-03_S20, Lib-04_S21, Lib-05_S22, Lib-06_S23), GFLV (Lib-04_S21), AGVd (Lib-03_S20), and GYSVd-2 (Lib-04_S21) viruses were only identified when we used the Geneious assembler. These viruses were validated in individual plants by RT-PCR (Supplementary Table S6).

When mapping all reads to the reference virus genomes, we also evaluated a number of Geneious program settings: low sensitivity, medium–low sensitivity, and custom sensitivity. As a result, we determined that, for our data, the best option was to map the reads using the ‘custom sensitivity’ settings. Using the default ‘medium–low sensitivity‘ settings of the program resulted in the mapping of non-specific reads and a large number of gaps. The ‘low sensitivity‘ parameters were found to be too strict for our data, i.e., reads with a small number of substitutions were not mapped to the reference sequence under these conditions. The results obtained from mapping the reads to reference virus sequences using different sensitivity parameters are shown in Supplementary Table S7.

The optimal pipeline for our data was found to be the pipeline ‘with *Vitis*’ including mapping of reads to reference sequences using the ‘custom sensitivity’ parameters.

Using this approach, we were able to identify contigs of 12 grapevine viruses, GFLV, GLRaV-1, GLRaV-2, including GLRaV-2RG (=GRSLaV [33]), GLRaV-3, GLRaV-4, GVA, GFkV, GRVFV, GSyV-1, GRSPaV, GPGV, and RBDV, as well as 4 viroids: HSVd, AGVd, GYSVd-1, and GYSVd-2 (Supplementary Table S6). In addition, we detected the contigs of the Arabis mosaic virus (ArMV), Grapevine deformation virus (GDeV), and Grapevine Red Globe virus (GRGV). The ArMV, GDeV, and GFLV viruses are known to be highly homologous [34,35]. As a result of the blastn analysis, we established that most ArMV and GDeV reads had high percent identity with the GFLV genome. A similar situation was observed in the analysis of contig mapping to the reference genomes of both GRGV and GFkV viruses, which belong to the genus *Maculavirus*, as well as GAMaV and GRVFV viruses, which belong to the *Marafivirus* genus [21,36,37].

According to the results of the blastn analysis of the de novo assembly of reads into contigs, none of the data processing algorithms allowed us to detect GVT. However, as a result of mapping the unique and redundant reads (‘with *Vitis’*) of each of the libraries to the reference genome GVT NC_035203, we were able to find 7787 non-redundant reads in the Lib-03_S20 library, one non-redundant read in the Lib-04_S21 library, and two redundant read in the Lib-05_S22 library (Supplementary Table S6). In a number of earlier studies devoted to the analysis of the grapevine virome, GVT was not detected in sRNA libraries [22,38,39,40]. However, a reanalysis of the results obtained in 2018 and 2020 [21,22] by bioinformatics and molecular methods confirmed the presence of the virus in Hungarian vineyards [41].

During the course of this study, based on the sRNA HTS results, we were unable to reconstruct the complete genomes of the detected viruses; however, there is evidence that this is possible [16,17,18]. Despite this, the approach based on using sRNA and combining material from several symptomatic samples for sequencing allowed us to perform monitoring and identify viruses present in the pooled sample at the lowest cost so that we could proceed to the analysis of each plant by RT-PCR at the next stage of validation. In the future, if a detailed analysis is required, it will be possible to sequence sRNA with large coverage or total RNA to obtain longer reads and carry out a detailed analysis of the virome of an individual sample.

### 3.3. Validation of the Results of sRNA HTS by RT-PCR and the Phylogenetic Analysis of Detected Viruses

**Figure 3:**
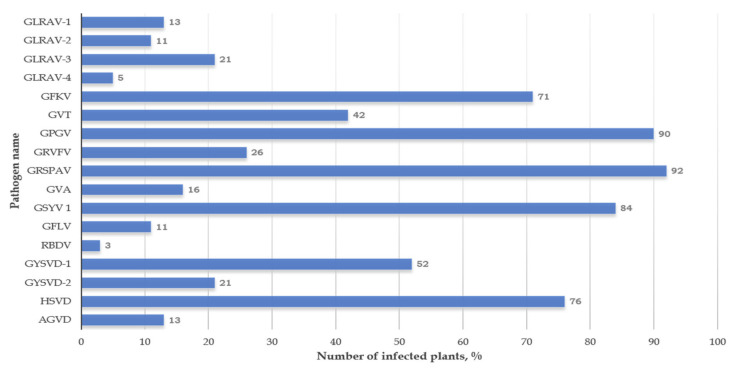
Frequency of occurrence of grapevine viruses (percentage of the total number of collected samples).

Summary of virus detection by RT-PCR is presented in Figure 3. The most frequently detected viruses in the samples were GFkV, GPGV, GRSPaV, GSyV-1, GYSVd-1, and HSVd. These pathogens were found in more than 50% of the samples.

Moreover, the analyzed samples contained viruses from the Closteroviridae family—GLRaV-1 (13% of samples), GLRaV-2 (11%), GLRaV-3 (21%), and GLRaV-4 (5%); the Betaflexiviridae family—GVT (42%) and GVA (16%); the Secoviridae family—GFLV (11%); and the Tymovidae family—GRVFV (26%). ArMV, GDeV, GRSLaV, and GRGV could not be detected in the samples, which confirms the results from the reinvestigation of the automated pipeline usage during the bioinformatics analysis. Based on the validation results, 37 out of 38 plants were determined to be simultaneously infected with 4 to 11 pathogens (Supplementary Table S8).

Thus, our results show that the reliability of HTS differs for the detection of different types of viruses, which is consistent with the data obtained earlier [21,22,42]. Grapevine diseases may be caused and modified by interactions among multiple infectious agents [43]. The symptoms cannot be correlated with a causative agent due to the existence of multiple infections in the vineyard.

As a next step, the nucleotide sequences obtained for each detected virus and viroid were phylogenetically analyzed. The numbers of sequences selected for the construction of a phylogenetic tree and the search for recombination sites of viral genome fragments are presented in Supplementary Table S9, while the results are presented below for each virus family.

**Closteroviridae.**

Currently, six different Grapevine leafroll-associated viruses are known (GLRaV-1, GLRaV-2, GLRaV-3, GLRaV-4, GLRaV-7, and GLRaV-13) [5]. Along with GFLV, GLRaV causes one of the most important viral grapevine diseases, affecting grapevines worldwide [44]. Previously, we identified GLRaV-1, GLRaV-2, and GLRaV-3 in Russia [29,45,46,47]. In this work, we were able to detect four Grapevine leafroll-associated viruses, namely GLRaV-1, GLRaV-2, GLRaV-3, and GLRaV-4; it should be noted that this was the first detection of GLRaV-4 in Russia.

GLRaV-1 was detected in five samples from three libraries. The Russian isolates cluster with isolates from China and Italy and belong to the second phylogenetic group (according to Lehad et al. (2019) [48] (Supplementary Figure S1). The coat protein genes were found to be 93.8% identical to each other and 89.6–94.4% identical to the GLRaV-1 reference sequence NC_016509 (Supplementary Table S4).

Eight recombination events were detected in GLRaV-1 coat protein genes (Supplementary Table S10). In the Russian isolate V1609 (MZ031984), potential parents were two isolates found in other vineyards: the major parent CA21 (JF811846.1) and the minor parent V471 (MW810492).

GLRaV-2 was detected in four samples from the Zs85-SV2_S19 library (10.5% of the total number of samples in the study). At the same time, we were unable to confirm the presence of the GLRaV-2RG strain, the contigs of which were mapped to GRSLaV NC_004724.1 using two pairs of primers. Using the restrictions of the GLR2CP1/GLR2CP2 fragment, we established that MZ065369 belongs to group 3, like other Russian isolates that we found earlier in Krasnodar Krai [29]. Phylogenetic analysis of the complete nucleotide sequence of the coat protein gene of the detected isolate showed that it is 87.9% identical to the sequence of the reference genome GLRaV-2 NC_007448. The Russian isolate was shown to cluster with isolates of the H4 CNP group [49], the Russian isolate published by us in 2019 [46], and isolates from Italy (Supplementary Figure S2).

Two recombination events were detected in GLRaV-2 coat protein genes (Supplementary Table S10).

GLRaV-3 was detected in three libraries and eight samples (21% of the total number of samples in the study). The sequences of the coat protein genes of the Russian isolates were found to have a high level of identity to each other (99.7%) and to the GLRaV-3 reference sequence NC_004667 (99–99.4%) (Supplementary Table S4). According to the phylogenetic analysis, the identified isolates clustered with representative isolates of phylogroup I (according to Diaz-Lara et al. (2018) [50]), next to isolates from Portugal, Chile, Greece, and China (Supplementary Figure S3).

Seven recombination events were detected in GLRaV-3 coat protein genes (Supplementary Table S10).

According to modern taxonomy, GLRaV-4 belongs to the *Ampelovirus* genus [51], while GLRaV-5, GLRaV-6, GLRaV-9, GLRaV-Car, GLRaV-Pr, and GLRaV-Ob are its strains [52]. During this study, GLRaV-4 was detected in only one sample in the Lib-03_S20 library. This virus was detected in Russia for the first time. We amplified a fragment of the *hsp70* sequence, and as a result, we found that our isolate had a rather low percentage identity (73.1%) to the GLRaV-4 reference sequence NC_016416 (Supplementary Table S4); however, the percentage identity of this fragment of the viral genome with the closest isolate from the USA was 96.4%. The phylogenetic analysis showed that the identified isolate clustered with isolates from the USA, and this was supported by a bootstrap value of 93% (Supplementary Figure S4).

Two recombination events were detected in GLRaV-4 sequences (Supplementary Table S10).

**Secoviridae.**

GFLV (genus *Nepovirus*), which is responsible for fanleaf degeneration disease, is one of the most important grapevine viruses worldwide. The presence of this pathogen has been confirmed on all continents where grapes are grown [53]. In the Russian territory, this virus was previously detected in Krasnodar Krai and the Republic of Crimea [29,54].

During the study, we identified only one representative of the genus *Nepovirus*, GFLV, which was detected in four samples from two libraries (10.5% of the total number of samples in the study). Analysis of the sequence of the RNA2 polyprotein gene fragment obtained as a result of sequencing showed that the Russian isolates were 95.6% identical to each other and 90.3–94.1% identical to the reference gene NC_003523 (Supplementary Table S4). According to the phylogenetic analysis, the identified isolates clustered into separate clades next to genetic variants from Italy, Spain, and Tunisia (Supplementary Figure S5).

Thirty-seven recombination events were detected in GFLV polyprotein sequences (Supplementary Table S10).

**Betaflexiviridae.**

GVA, a member of the *Vitivirus* genus, is one of the most common grapevine viruses. According to the literature, GVA is found in almost all regions of the world where grapes are grown [55,56]. In Russia, we previously detected GVA in vineyards in the Republic of Crimea and Krasnodar Krai [29,57].

During this study, we were able to detect GVA in six plants from three libraries. The coat protein genes of the detected isolates were 87.5% identical to each other and 87.2–88.8% identical to the reference genome GVA NC_003604 (Supplementary Table S4). As a result of the phylogenetic analysis of the coat protein genes of the Russian isolates, we identified three GVA molecular groups (according to Alabi et al. (2014) [58]): I, II, and IV. The isolates clustered with genetic variants from Greece, China, and the USA (Supplementary Figure S6). Moreover, one isolate clustered separately from representative isolates of the previously described GVA molecular groups.

Eight recombination events were found in GVA sequences (Supplementary Table S10).

GRSPaV (*Foveavirus*) is a widespread virus that infects grapevines [21,59,60]. In Russia, it has been found in commercial vineyards [29,61]. The data obtained in this study are in line with the trend showing that the virus is widespread in vineyards worldwide [62,63,64]. GRSPaV was detected in all libraries by sRNA HTS and in 35 samples using RT-PCR. For the phylogenetic analysis, we amplified and sequenced 22 sequences of the GRSPaV coat protein genes, which were 88.2% identical to each other and 81.9–98.5% identical to the GRSPaV reference sequence NC_001948 (Supplementary Table S4). The Russian isolates had a uniform distribution throughout the phylogenetic tree. Eleven isolates belonged to the GRSPaV molecular group III, four to group VII, two to group I, and three to group II (according to Hily et al. (2018) and Selmi et al. (2020) [65,66]). Two isolates clustered separately from the representative isolates of the previously described GRSPaV molecular groups (Supplementary Figure S7).

Twenty-three recombination events were found in GRSPaV coat protein genes (Supplementary Table S10), which may explain why frequent recombination can occur [67] as well as the existence of several remarkably different strains of this virus.

Another representative of the *Foveavirus* genus that we detected in the analyzed libraries was GVT. Recently, an analysis of the results of total RNA library sequencing allowed us to identify this virus in a commercial vineyard [47]. In this study, as a result of the bioinformatics analysis, GVT reads were found in three libraries. The sequencing data validation approach that we used (first examining the RNA library pools and then, for positive results, examining each sample for the presence of the virus) made it possible to identify GVT in all five sRNA libraries and in 16 samples (42.1% of the total number of samples in the study). Phylogenetic analysis of the coat protein genes of the Russian GVT isolates and sequences from GenBank showed a uniform distribution of the Russian isolates throughout the phylogenetic tree (Supplementary Figure S8). Our isolates clustered together with isolates that were previously assigned to groups IV, V, and I by Demian et al. (2021) [41]. Sequencing of sRNAs for the detection of viruses of the *Foveavirus* genus was performed previously, in particular, for GRSPaV [16,17,18]. However, in these studies, scientists were not able to detect GVT. Our results support the idea that the combined application of computer analysis methods for sequenced sRNAs and molecular biological methods is necessary for the most complete characterization of the grapevine virome, at least for some viruses, including GVT.

Five recombination events were detected in GVT sequences (Supplementary Table S10).

Since the discovery of GPGV (*Trichovirus*) [16], the virus has been shown to be widespread in vineyards around the world, including in France [68], USA [69], Republic of Korea [70], Turkey [71], China [72], Canada [73], Germany [74], Pakistan [75], Spain [76], Brazil [77], Chile [78], Lebanon [79], Moldova [80], Georgia [81], Iran [82], Australia [83], Czech Republic [84], Algeria [85], England [86], Armenia [87], Bulgaria [88], Argentina [89], California [90], Japan [91], Belgium [92], Romania [93], Hungary [21], and Russia [47]. In 89% (34 out of 38) of the samples, we detected GPGV. Among those found, only two plants clearly contained symptomatic versions, while the rest of the plants contained asymptomatic variants. This could support the idea that the asymptomatic version is widespread [21,22,94]. The nucleotide sequences of the coat protein genes of the Russian isolates were 97.9% identical to each other and 96.4–98.3% identical to the GPGV reference sequence NC_015782 (Supplementary Table S4). On the phylogenetic tree, the Russian isolates clustered next to isolates from Hungary, Brazil, France, Spain, Slovakia, Algeria, and Italy (Supplementary Figure S9).

One recombination event was detected in the GPGV coat protein gene (Supplementary Table S10).

**Tymovidae**.

GFkV (genus *Maculavirus*) is widespread worldwide [95]. Recently, it was detected in the territories of Bosnia and Herzegovina [96], the USA [97], Canada [98], Macedonia [99], the Republic of Korea [100], and the United Kingdom [101], but it is known to also be present in other countries. This is the first time that this virus has been described in the Russian territory [29]. We detected GFkV in all libraries by the bioinformatics method (Supplementary Table S6) and by RT-PCR (27 samples, 71.1%). The coat protein genes of the detected isolates were 94.5–97.9% identical to the Italian GFkV reference sequence NC_003347 and 96.1% identical to each other (Supplementary Table S4). The Russian isolates had a uniform distribution throughout the phylogenetic tree (Supplementary Figure S10). The analysis found no recombination events in GFkV coat protein genes (Supplementary Table S10).

Since the discovery of GRVFV (tentative genus *Marafivirus*) in vineyards in Greece (Elbeaino et al. (2001)), this virus has been found in a number of European countries. It has also been detected in China [102], Australia [103], Canada [104], Pakistan [105], Iran [106], Japan [107], and the Republic of Korea [108]. GRVFV was first detected in Russia during the analysis of the virome of a vineyard [47]. In this study, we detected GRVFV using bioinformatics methods in each library and validated its presence in 10 samples (26.3%) (Supplementary Table S10). Comparison of the genome fragments of Russian isolates showed that they were 87.7% identical to each other and 84.2–91.8% identical to the GRVFV reference sequence NC_034205, which indicates the high genetic diversity of this virus (Supplementary Table S4). The Russian isolates had a uniform distribution throughout the phylogenetic tree (Supplementary Figure S11). The analysis found no recombination events in sequences of the GRVFV genome fragments (Supplementary Table S10).

GSyV-1 (*Marafivirus*) has been described as a virus that infects grapevines in the USA [109], Chile [110], China [111], Spain [112], Italy [16], Republic of Korea [113], Hungary [114], Africa [115], Turkey [116], Slovakia [117], and other countries. This virus was found for the first time here in the Russian territory. GSyV-1 was detected using bioinformatics methods in all libraries, and this was confirmed by RT-PCR (33 samples infected, or 86.8%) (Supplementary Table S4 and Supplementary Table S9). For the phylogenetic analysis, the coat protein genes of 11 isolates were amplified. The obtained sequences were 93.9% identical to each other and 91.4–98.3% identical to the GSyV-1 reference sequence NC_012484 (Supplementary Table S4). The results of the phylogenetic sequence analysis of the GSyV-1 coat protein gene are provided in Supplementary Figure S12. The GSyV-1 isolates obtained for the first time in Russia clustered with isolates from Brazil, Hungary, and USA; one isolate (MZ436820) clustered separately (Supplementary Figure S12). The analysis found no recombination events in GSyV-1 coat protein genes (Supplementary Table S10).

**Idaeovirus**.

RBDV is an economically important virus that infects raspberries and grapevines [118]. It has been identified on raspberries in Slovenia, Hungary, Turkey, Japan, China, Argentina, Sweden, Finland, and Belarus and on grapevines in Slovenia [119], Hungary [120], and Serbia [121]. This virus was detected here for the first time in Russia; it was not previously detected on either raspberries or grapevines. Using the bioinformatics analysis, RBDV was found in one library, and using RT-PCR, its presence was shown in one sample. The phylogenetic analysis using a 930 bp fragment of the RNA1 gene of RBDV (MZ457018) showed that the Russian isolate clustered next to isolates from Serbia, Hungary, and Ecuador (Supplementary Figure S13), while the phylogenetic analysis using the sequence of the RNA2 coat protein gene of RBDV (MZ457017) showed that the Russian isolate clustered next to isolates from Slovenia and Hungary (Supplementary Figure S13). It is worth noting that these isolates were sampled from both *Rubus* sp. and *Vitis* sp. In addition, a fragment of the RBDV RNA1 gene clustered next to isolates that were found not only on grapevines, but also on *Rubus* sp., *Fraxinus excelsior*, and *Chenopodium quinoa* plants, which indicates that there is a wide range of host plants for this pathogen. The nucleotide sequence of the RBDV coat protein gene (MZ457017) obtained by us was found to be 95.4% identical to the RBDV RNA2 reference sequence NC_003740. Moreover, a fragment of the RBDV RNA1 gene obtained as a result of data validation (MZ457018) was 94.4% identical to the RBDV RNA1 reference sequence NC_003739 (Supplementary Table S4). The analysis found no recombination events in RBDV sequences.

**Viroids.**

Grapevine viroids have been known for a long time and are widespread worldwide [122]. Currently, six viroids that infect grapevine are known [123]. Previously, we found HSVd and GYSVd-1 in Russia [47]. In this study, we detected the presence of four viroids: HSVd, AGVd, GYSVd-1 and GYSVd-2. It should be noted that AGVd and GYSVd-2 were identified in Russia for the first time.

Among all known viroids, HSVd has the widest range of host plants [124,125,126]. To date, the GenBank contains HSVd sequences found on hop, wild apple tree, apricot, sweet cherry, sour cherry, hibiscus, cucumber, grapevine, citrus fruits, plum, and peach [127]. As a result of the phylogenetic analysis of HSVd sequences, the Russian isolates were classified as belonging to the Plum–Hop/Cit3 group and the Hop group (Supplementary Figure S14). The sequences were found to be 98.8% identical to each other and 91.4–95.8% identical to the HSVd reference sequence NC_001351 (Supplementary Table S4). The analysis found no recombination events in HSVd sequences.

As a result of the bioinformatics analysis, GYSVd-1 was detected in all sRNA libraries, and this was confirmed by RT-PCR. It should be noted that in order to validate GYSVd-1, the number of amplification cycles for all libraries had to be increased from 40 to 45. PCR products obtained under the new conditions were also sequenced and their levels of similarity to GYSVd-1 were confirmed. The nucleotide sequences of these isolates were 97.8% identical to each other and 87.7–99.4% identical to the GYSVd-1 reference sequences NC_001920 (Supplementary Table S4). The Russian isolates of GYSVd-1 clustered on the phylogenetic tree next to isolates from Australia, Turkey, Spain, Chile, and Hungary (Supplementary Figure S15). No recombination events were detected in GYSVd-1 sequences.

GYSVd-2 was found in four out of five libraries (Zs85-SV2_S19, Lib-03_S20, Lib-04_S21, Lib-05_S22). In addition, according to the results of the de novo assembly by the SPAdes assembler, one contig (42 nucleotides) of GYSVd-2 was found in the Lib-06_S23 library, but the sequence was not amplified by RT-PCR. Moreover, the results of the blastn analysis showed that contig was 92.7% identical to the GYSVd-1 genome, which, together with the validation of GYSVd-1 by RT-PCR in the library Lib-06_S23, indicates that only GYSVd-1 is present in this library. The nucleotide sequences of the identified isolates were 97.9% identical to each other and 96.2–99.5% identical to the GYSVd-2 reference sequence NC_003612 (Supplementary Table S4). The Russian isolates of GYSVd-2 clustered on the phylogenetic tree next to isolates from China, Iran, India, the Republic of Korea, and Nigeria (Supplementary Figure S16). No recombination events were detected in sequences of GYSVd-2.

AGVd has been found in many grape-producing countries, such as Australia [128], Tunisia [129,130], USA [42], China [131], Iran [132], Italy [133], India [134], Thailand [135], and Greece [136]. We identified AGVd in two out of five libraries (Zs85-SV2_S19 and Lib-03_S20) in five samples. The nucleotide sequences of the Russian isolates were 93.2% identical to each other and 93.3–99% identical to the AGVd reference sequence NC_003553. On the phylogenetic tree, the Russian isolates clustered next to isolates from China, Iran, Australia, and India (Supplementary Figure S17). No recombination events were identified in AGVd sequences.

Thus, in this study, we used the HTS method to analyze sRNA libraries for the detection of plant viruses for the first time in Russia. The reliability and power of this approach were confirmed, making it possible to identify a wide species composition for the grapevine virome. Moreover, we detected five grapevine viruses and viroids for the first time in the Russian territory: GLRaV-4, GSyV-1, RBDV, AGVd, and GYSVd-2. Another 10 viruses and 2 viroids previously detected in Russia, some of which are economically important ones (for example, GLRaV-1, GLRaV-2, GLRaV-3, GVA, GFLV), were also detected by us in commercial vineyards.

The approach of using sRNA and combining material from several symptomatic samples for sequencing allowed us to carry out monitoring and determine the virome of the combined sample at the lowest cost, and at the next stage of validation, we were able to analyze the virome of an individual plant by RT-PCR. The detection of at least a few contigs or reads of a virus in a plant allows additional research to be performed in the future and the complete genome of the detected virus to be assembled using higher coverage of sequencing sRNA or, more preferably, the total RNA from an individual sample.

Our data show that exact analysis of the virome of each sample requires a combination of the sRNA HTS method and molecular biology-based methods. In particular, Sanger sequencing of the coat protein genes of the detected viruses made it possible to carry out a phylogenetic analysis and identify the molecular groups of the studied viruses. The phylogenetic analysis of the sequences of the detected viruses identified the presence of GVA in groups I, II and IV; GRSPaV in groups I, II, III, VII, and VIII; GVT in groups I, IV, and V; GLRaV-1 in group II; GLRaV-2 in group H4 CNP; GLRaV-3 in group I; and HSVd in groups Hop and Plum–Hop/Cit3. The clustering of the Russian isolates (HSVd, GLRaV-1, GRSPaV, and GVA) separately from the representative members of the groups from the GenBank database indicates their genetic diversity and suggests the presence of new molecular groups. This suggests that further analysis of the complete genomes of these viruses is needed. In addition, our recombination analysis of genome fragments of detected viruses allowed us to expand knowledge about their genetic variability and to discover new recombination sites for further confirmation using molecular biological methods.

Our research shows the need for the use of different approaches in the analysis of bioinformatics data. In particular, the use of two assemblers and the analysis of data without removing the reads of the host plant made it possible to thoroughly characterize the species composition of the virome. Moreover, using GVT as an example, we demonstrated that, at least for some viruses, mapping of reads from sRNA libraries to the reference genomes of grapevine viruses and viroids leads to the most reliable detection.

Both well-known varieties, such as Merlot, Saperavi, and Cabernet Sauvignon, and valuable local wine varieties, Krasnostop Zolotovsky, for example, were affected by a number of viruses, including economically important ones. The largest number of viruses was found in the samples of table varieties that can be explained by the source of the planting material. Wine varieties are much more common in Russia, but the importance of table varieties is also great; therefore, it is important to use high-quality planting materials and methods of viral infection elimination, which should be introduced in the grapevine selection.

In this study, among the detected viruses were both the economically important GLRaV-1, GLRaV-3, and GFLV, and viruses that were recently discovered (GVT, GSyV-1, and so on); their harmfulness is still being studied. Economically important viruses cause systemic infections in vineyards, reducing the yield and quality of berries, and they are used in the certification of planting material in many countries. Of particular importance are viruses that do not show symptoms and do not strongly affect the yield but are important for grafting, for example, and can interact with other viruses and strengthen their symptoms. Thus, it is important to obtain the most complete information about the viruses that cause the symptoms using metagenomics methods, as well as the viruses of which the infection is asymptomatic. In addition, the information about the presence of a virus complex in the virome can help in phenomics and in the study of the correlation between the manifestation of symptoms and the presence of viruses in a plant.

## 4. Conclusions

The application of the HTS method to the analysis of sRNA libraries in order to study the biodiversity of viral pathogens in Russian vineyards was shown to be expedient and useful. The complexity of virus populations detected in Krasnodar Krai highlights the need for detection methods that are able to identify all viruses and their variants in vineyards. A lack of systemic phytosanitary monitoring and agrotechnical, pest, carrier, and vector control are possible reasons for the presence of multiple infections. As the isolates are usually not clustered together on the phylogenetic trees, geographically, it is highly possible that they entered the country independently through infected planting material. The study of viral associations, with multiple infections, can help in the study of pathogenic effects on host plants as well as in the certification of plant material when laying vineyards. A comprehensive, coordinated approach is imperative to reduce the viral load.

## Data Availability

Representative sequences were deposited in GenBank under the accession numbers MZ031984, MZ065366-MZ065369, MW810492, MZ605444, MZ065370-MZ065375, MZ091480-MZ091497, MZ209411, MZ229615-MZ229625, MZ464054, MZ476948-MZ476966, MZ146886-MZ146892, MZ146898, MZ579707, MZ146893-MZ146897, MZ158160-MZ158182, MZ268015, MZ436819-MZ436829, MZ196509-MZ196513, MZ457017, MZ457018, MZ835424-MZ835433, MZ196514, MZ665997-MZ665999, MZ803184, MZ196515, MZ825532-MZ825549, MZ196517, MZ603185, and MZ196516.

## Supplementary Materials

The following are available online at https://www.mdpi.com/article/10.3390/v13122432/s1. Supplementary Figures: Figure S1: Phylogenetic analysis of global isolates of GLRaV-1; Figure S2: Phylogenetic analysis of global isolates of GLRaV-2; Figure S3: Phylogenetic analysis of global isolates of GLRaV-3; Figure S4: Phylogenetic analysis of global isolates of GLRaV-4; Figure S5: Phylogenetic analysis of global isolates of GFLV; Figure S6: Phylogenetic analysis of global isolates of GVA; Figure S7: Phylogenetic analysis of global isolates of GRSPaV; Figure S8: Phylogenetic analysis of global isolates of GVT; Figure S9: Phylogenetic analysis of global isolates of GPGV; Figure S10: Phylogenetic analysis of global isolates of GFkV; Figure S11: Phylogenetic analysis of global isolates of GRVFV; Figure S12: Phylogenetic analysis of global isolates of GSyV-1; Figure S13: Phylogenetic analysis of global isolates of RBDV; Figure S14: Phylogenetic analysis of global isolates of HSVd; Figure S15: Phylogenetic analysis of global isolates of GYSVd-1; Figure S16: Phylogenetic analysis of global isolates of GYSVd-2; Figure S17: Phylogenetic analysis of global isolates of AGVd. Supplementary Tables: Table S1: Basic information about the sampled vineyards; Table S2: Primers used for validation of the HTS results [28,29,39,47,61,117,137,138,139,140,141,142,143,144,145,146,147,148,149,150,151]; Table S3: Primers used for the phylogenetic analysis [21,29,47,57,61,110,142,144,145,147,149,152,153,154,155,156,157]; Table S4: Amplified sequences of viruses uploaded into GenBank with their identifiers; Table S5: Initial statistics for the sequencing results; Table S6: Summary of the two bioinformatic approaches used in the study for the sRNA HTS analysis according to libraries and viruses; Table S7: Supplementary information on the bioinformatics analysis on different sensitivity levels; Table S8: Supplementary information on HTS validation by RT-PCR; Table S9: Initial data for the alignment of virus genomes; Table S10: Test of novel recombination events calculated for viruses. Photographic material showing symptoms. This supplementary material contains photographs of all symptomatic samples used in the experiment.

## References

[1] Meng B., Martelli G.P., Golino D.A., Fuchs M. (2017). The Grapevine, Viticulture, and Winemaking: A Brief Introduction. Grapevine Viruses: Molecular Biology, Diagnostics and Management.

[2] Mannini F., Digiaro M., Meng B., Martelli G.P., Golino D.A., Fuchs M. (2017). The Effects of Viruses and Viral Diseases on Grapes and Wine. Grapevine Viruses: Molecular Biology, Diagnostics and Management.

[3] Martelli G.P., Meng B., Martelli G.P., Golino D.A., Fuchs M. (2017). An Overview on Grapevine Viruses, Viroids, and the Diseases They Cause. Grapevine Viruses: Molecular Biology, Diagnostics and Management.

[4] Martelli G. Where Grapevine Virology Is Heading to. Proceedings of the 19th Congress of the International Council for the Study of Virus and Virus-like Diseases of the Grapevine (ICVG).

[5] Fuchs M. (2020). Grapevine Viruses: A Multitude of Diverse Species with Simple but Overall Poorly Adopted Management Solutions in the Vineyard. J. Plant Pathol..

[6] Digiaro M., Elbeaino T., Martelli G.P., Meng B., Martelli G.P., Golino D.A., Fuchs M. (2017). Grapevine Fanleaf Virus and Other Old World Nepoviruses. Grapevine Viruses: Molecular Biology, Diagnostics and Management.

[7] Naidu R.A., Meng B., Martelli G.P., Golino D.A., Fuchs M. (2017). Grapevine Leafroll-Associated Virus 1. Grapevine Viruses: Molecular Biology, Diagnostics and Management.

[8] Angelini E., Aboughanem-Sabanadzovic N., Dolja V.V., Meng B., Meng B., Martelli G.P., Golino D.A., Fuchs M. (2017). Grapevine Leafroll-Associated Virus 2. Grapevine Viruses: Molecular Biology, Diagnostics and Management.

[9] Burger J.T., Maree H.J., Gouveia P., Naidu R.A., Meng B., Martelli G.P., Golino D.A., Fuchs M. (2017). Grapevine Leafroll-Associated Virus 3. Grapevine Viruses: Molecular Biology, Diagnostics and Management.

[10] Minafra A., Mawassi M., Goszczynski D., Saldarelli P., Meng B., Martelli G.P., Golino D.A., Fuchs M. (2017). Grapevine Vitiviruses. Grapevine Viruses: Molecular Biology, Diagnostics and Management.

[11] Boonham N., Kreuze J., Winter S., van der Vlugt R., Bergervoet J., Tomlinson J., Mumford R. (2014). Methods in Virus Diagnostics: From ELISA to next Generation Sequencing. Virus Res..

[12] Massart S., Olmos A., Jijakli H., Candresse T. (2014). Current Impact and Future Directions of High Throughput Sequencing in Plant Virus Diagnostics. Virus Res..

[13] Al Rwahnih M., Daubert S., Golino D., Islas C., Rowhani A. (2015). Comparison of Next-Generation Sequencing Versus Biological Indexing for the Optimal Detection of Viral Pathogens in Grapevine. Phytopathology.

[14] Roossinck M.J. (2017). Deep Sequencing for Discovery and Evolutionary Analysis of Plant Viruses. Virus Res..

[15] Roossinck M.J., Martin D.P., Roumagnac P. (2015). Plant Virus Metagenomics: Advances in Virus Discovery. Phytopathology.

[16] Giampetruzzi A., Roumi V., Roberto R., Malossini U., Yoshikawa N., La Notte P., Terlizzi F., Credi R., Saldarelli P. (2012). A New Grapevine Virus Discovered by Deep Sequencing of Virus- and Viroid-Derived Small RNAs in Cv Pinot Gris. Virus Res..

[17] Maliogka V.I., Olmos A., Pappi P.G., Lotos L., Efthimiou K., Grammatikaki G., Candresse T., Katis N.I., Avgelis A.D. (2015). A Novel Grapevine Badnavirus Is Associated with the Roditis Leaf Discoloration Disease. Virus Res..

[18] Zhang Y., Singh K., Kaur R., Qiu W. (2011). Association of a Novel DNA Virus with the Grapevine Vein-Clearing and Vine Decline Syndrome. Phytopathology.

[19] Blouin A.G., Keenan S., Napier K.R., Barrero R.A., MacDiarmid R.M. (2018). Identification of a Novel Vitivirus from Grapevines in New Zealand. Arch. Virol..

[20] Blouin A.G., Chooi K.M., Warren B., Napier K.R., Barrero R.A., MacDiarmid R.M. (2018). Grapevine Virus I, a Putative New Vitivirus Detected in Co-Infection with Grapevine Virus G in New Zealand. Arch. Virol..

[21] Czotter N., Molnar J., Szabó E., Demian E., Kontra L., Baksa I., Szittya G., Kocsis L., Deak T., Bisztray G. (2018). NGS of Virus-Derived Small RNAs as a Diagnostic Method Used to Determine Viromes of Hungarian Vineyards. Front. Microbiol..

[22] Demian E., Jaksa-Czotter N., Molnar J., Tusnady G.E., Kocsis L., Varallyay E. (2020). Grapevine Rootstocks Can Be a Source of Infection with Non-Regulated Viruses. Eur. J. Plant Pathol..

[23] Pooggin M.M. (2018). Small RNA-Omics for Plant Virus Identification, Virome Reconstruction, and Antiviral Defense Characterization. Front. Microbiol..

[24] Morante-Carriel J., Sellés-Marchart S., Martínez-Márquez A., Martínez-Esteso M.J., Luque I., Bru-Martínez R. (2014). RNA Isolation from Loquat and Other Recalcitrant Woody Plants with High Quality and Yield. Anal. Biochem..

[25] Geneious Bioinformatics Software for Sequence Data Analysis. https://www.geneious.com/.

[26] FinchTV.

[27] Sayers E.W., Cavanaugh M., Clark K., Ostell J., Pruitt K.D., Karsch-Mizrachi I. (2020). GenBank. Nucleic Acids Res..

[28] Bertazzon N., Angelini E. (2004). Advances in the Detection of Grapevine Leafroll-Associated Virus 2 Variants. J. Plant Pathol..

[29] Porotikova E., Terehova U., Volodin V., Yurchenko E., Vinogradova S. (2021). Distribution and Genetic Diversity of Grapevine Viruses in Russia. Plants.

[30] Tamura K., Nei M. (1993). Estimation of the Number of Nucleotide Substituions in the Control Region of Mitochondrial DNA in Humans and Chimpanzees. Mol. Biol. Evol..

[31] Martin D.P., Murrell B., Golden M., Khoosal A., Muhire B. (2015). RDP4: Detection and Analysis of Recombination Patterns in Virus Genomes. Virus Evol..

[32] Mekuria T.A., Gutha L.R., Martin R.R., Naidu R.A. (2009). Genome Diversity and Intra- and Interspecies Recombination Events in Grapevine Fanleaf Virus. Phytopathology.

[33] Alkowni R., Zhang Y.-P., Rowhani A., Uyemoto J.K., Minafra A. (2011). Biological, Molecular, and Serological Studies of a Novel Strain of Grapevine Leafroll-Associated Virus 2. Virus Genes.

[34] Elbeaino T., Digiaro M., Ghebremeskel S., Martelli G.P. (2012). Grapevine Deformation Virus: Completion of the Sequence and Evidence on Its Origin from Recombination Events between Grapevine Fanleaf Virus and Arabis Mosaic Virus. Virus Res..

[35] Vigne E., Marmonier A., Fuchs M. (2008). Multiple Interspecies Recombination Events within RNA2 of Grapevine Fanleaf Virus and Arabis Mosaic Virus. Arch. Virol..

[36] Cretazzo E., Padilla C.V., Velasco L. First Report of Grapevine Red Globe Virus in Grapevine in Spain. https://www.cabi.org/ISC/abstract/20173050622.

[37] Sabanadzovic S., Abou-Ghanem N., Castellano M.A., Digiaro M., Martelli G.P. (2000). Grapevine Fleck Virus-like Viruses in Vitis. Arch. Virol..

[38] Chiumenti M., Giampetruzzi A., Pirolo C., Morelli M., Saldarelli P., Minafra A., Bottalico G., La Notte P., Campanale A., Savino V. Approaches of next Generation Sequencing to Investigate Grapevine Diseases of Unknown Etiology. Proceedings of the 17th Congress of ICVG.

[39] Eichmeier A., Komínková M., Komínek P., Baránek M. (2016). Comprehensive Virus Detection Using Next Generation Sequencing in Grapevine Vascular Tissues of Plants Obtained from the Wine Regions of Bohemia and Moravia (Czech Republic). PLoS ONE.

[40] Sidharthan V.K., Sevanthi A.M., Jaiswal S., Baranwal V.K. (2020). Robust Virome Profiling and Whole Genome Reconstruction of Viruses and Viroids Enabled by Use of Available MRNA and SRNA-Seq Datasets in Grapevine (*Vitis vinifera* L.). Front. Microbiol..

[41] Demian E., Holczbauer A., Galbacs Z.N., Jaksa-Czotter N., Turcsan M., Olah R., Varallyay E. (2021). Variable Populations of Grapevine Virus T Are Present in Vineyards of Hungary. Viruses.

[42] Al Rwahnih M., Daubert S., Golino D., Rowhani A. (2009). Deep Sequencing Analysis of RNAs from a Grapevine Showing Syrah Decline Symptoms Reveals a Multiple Virus Infection That Includes a Novel Virus. Virology.

[43] Komar V., Vigne E., Demangeat G., Fuchs M. (2007). Beneficial Effect of Selective Virus Elimination on the Performance of *Vitis vinifera* Cv. Chardonnay. Am. J. Enol. Vitic..

[44] Maree H.J., Almeida R.P.P., Bester R., Chooi K.M., Cohen D., Dolja V.V., Fuchs M.F., Golino D.A., Jooste A.E.C., Martelli G.P. (2013). Grapevine Leafroll-Associated Virus 3. Front. Microbiol..

[45] Porotikova E.V., Risovannaya V.I., Volkov Y.A., Dmitrenko U.D., Volodin V.A., Gorislavets S.M., Stranishevskaya E.P., Agranovsky A.A., Kamionskaya A.M., Vinogradova S.V. (2016). Occurrence of Grapevine Leafroll-Associated Viruses-1 and -3 in Crimea. Moscow Univ. Biol. Sci. Bull..

[46] Porotikova E.V., Dmitrenko U.D., Yurchenko E.G., Vinogradova S.V. (2019). First Report of Grapevine Leafroll-Associated Virus 2 in Russian Grapevines (*Vitis vinifera*). Plant Dis..

[47] Vinogradova S.V., Navrotskaya E.V., Porotikova E.V., Massart S., Varallyay E. (2021). The First Virome of Russian Vineyard. Plants.

[48] Lehad A., Selmi I., Louanchi M., Aitoua M., Mahfoudhi N. (2019). Occurrence and Diversity of Grapevine Leafroll—Associated Virus 1 in Algeria. Phytopathol. Mediterr..

[49] Fonseca F., Esteves F., Teixeira Santos M., Brazao J., Eiras-Dias J.E. (2016). Genetic Variants of Grapevine Leafroll-Associated Virus 2 Infecting Portuguese Grapevine Cultivars. Phytopathol. Mediterr..

[50] Diaz-Lara A., Klaassen V., Stevens K., Sudarshana M.R., Rowhani A., Maree H.J., Chooi K.M., Blouin A.G., Habili N., Song Y. (2018). Characterization of Grapevine Leafroll-Associated Virus 3 Genetic Variants and Application towards RT-QPCR Assay Design. PLoS ONE.

[51] Lefkowitz E.J., Dempsey D.M., Hendrickson R.C., Orton R.J., Siddell S.G., Smith D.B. (2018). Virus Taxonomy: The Database of the International Committee on Taxonomy of Viruses (ICTV). Nucleic Acids Res..

[52] Adiputra J., Jarugula S., Naidu R.A. (2019). Intra-Species Recombination among Strains of the Ampelovirus Grapevine Leafroll-Associated Virus 4. Virol. J..

[53] Andret-Link P., Laporte C., Valat L., Ritzenthaler C., Demangeat G., Vigne E., Laval V., Pfeiffer P., Stussi-Garaud C., Fuchs M. (2004). Grapevine fanleaf virus: Still a major threat to the grapevine industry. J. Plant Pathol..

[54] Volodin V., Gorislavets S., Risovannaya V., Stranishevskaya E., Shadura N., Volkov Y., Matveykina E. (2020). Detection of a Viral Infection Complex (GLRAV-1, -3 and GFLV) in Vineyards of Crimea. Vitic. Winemak..

[55] Ebrahimi Ghomi M., Shamsbakhsh M., Pourrahim R. (2007). Study on the status of three grapevine viruses in North-Eastern vineyards of Iran. Appl. Entomol. Phytopathol..

[56] Rakhshandehroo F., Pourrahim R., Zadeh H.Z., Rezaee S., Mohammadi M. (2005). Incidence and Distribution of Viruses Infecting Iranian Vineyards. J. Phytopathol..

[57] Porotikova E.V., Dmitrenko U.D., Volodin V.A., Volkov Y.A., Gorislavets S.M., Stranishevskaya E.P., Risovannaya V.I., Kamionskaya A.M., Vinogradova S.V. (2016). First Report of Grapevine Virus A in Russian Grapevines. Plant Dis..

[58] Alabi O.J., Rwahnih M.A., Mekuria T.A., Naidu R.A. (2014). Genetic Diversity of Grapevine Virus A in Washington and California Vineyards. Phytopathology.

[59] Martelli G. (1993). Rugose Wood Complex. Graft-Transmissible Disease of Grapevines, Handbook for Detection and Diagnosis.

[60] Zhang Y.-P., Uyemoto J.K., Golino D.A., Rowhani A. (1998). Nucleotide Sequence and RT-PCR Detection of a Virus Associated with Grapevine Rupestris Stem-Pitting Disease. Phytopathology.

[61] Dmitrenko U.D., Porotikova E.V., Gorislavets S.M., Risovannaya V.I., Volkov Y.A., Stranishevskaya E.P., Kamionskaya A.M., Vinogradova S.V. (2016). First Report of Grapevine Rupestris Stem Pitting-Associated Virus in Russia. Plant Dis..

[62] Komorowska B., Berniak H., Golis T. (2014). Detection of Grapevine Viruses in Poland. J. Phytopathol..

[63] Pacifico D., Stigliano E., Sposito L., Spinelli P., Garfì G., Gristina A.S., Fontana I., Carimi F. (2016). Survey of Viral Infections in Spontaneous Grapevines from Natural Environments in Sicily. Eur. J. Plant Pathol..

[64] Katarina H., Saldarelli P., Čarija M., Černi S., Goran Z., Mucalo A., Tomislav R. (2020). Predominance and Diversity of GLRaV-3 in Native Vines of Mediterranean Croatia. Plants.

[65] Hily J.-M., Candresse T., Garcia S., Vigne E., Tannière M., Komar V., Barnabé G., Alliaume A., Gilg S., Hommay G. (2018). High-Throughput Sequencing and the Viromic Study of Grapevine Leaves: From the Detection of Grapevine-Infecting Viruses to the Description of a New Environmental Tymovirales Member. Front. Microbiol..

[66] Selmi I., Pacifico D., Lehad A., Stigliano E., Crucitti D., Carimi F., Naima M. Genetic Diversity of Grapevine Rupestris Stem Pitting-Associated Virus Isolates from Tunisian Grapevine Germplasm—Selmi—2020—Plant Pathology—Wiley Online Library. https://bsppjournals.onlinelibrary.wiley.com/doi/full/10.1111/ppa.13183?casa_token=QfyTerWj6LwAAAAA%3AK3ug7fUFAs91pMxBeulOZ4YFpR7LKLdCsPYdlycRc0fK8AvOk8lFHyRIx3ouoH7zhJCG3_99W2j9ci8vMaw.

[67] Glasa M., Predajna L., Soltys K., Sihelska N., Nagyova A., Wetzel T., Sabanadzovic S. (2017). Analysis of Grapevine Rupestris Stem Pitting-Associated Virus in Slovakia Reveals Differences in Intra-Host Population Diversity and Naturally Occurring Recombination Events. Plant Pathol. J..

[68] Beuve M., Candresse T., Tannières M., Lemaire O. (2015). First Report of Grapevine Pinot Gris Virus (GPGV) in Grapevine in France. Plant Dis..

[69] Al Rwahnih M., Golino D., Rowhani A. (2015). First Report of Grapevine Pinot Gris Virus Infecting Grapevine in the United States. Plant Dis..

[70] Cho I.S., Jung S.M., Cho J.D., Choi G.S., Lim H.S. (2013). First Report of *Grapevine Pinot Gris Virus* Infecting Grapevine in Korea. N. Dis. Rep..

[71] Gazel M., Caglayan K., Elçi E., Öztürk L. (2015). First Report of Grapevine Pinot Gris Virus in Grapevine in Turkey. Plant Dis..

[72] Fan X.D., Dong Y.F., Zhang Z.P., Ren F., Hu G.J., Li Z.N., Zhou J. (2015). First Report of Grapevine Pinot Gris Virus in Grapevines in China. Plant Dis..

[73] Xiao H., Shabanian M., McFadden-Smith W., Meng B. (2015). First Report of Grapevine Pinot Gris Virus in Commercial Grapes in Canada. Plant Dis..

[74] Reynard J.-S., Schumacher S., Menzel W., Fuchs J., Bohnert P., Glasa M., Wetzel T., Fuchs R. (2016). First Report of Grapevine Pinot Gris Virus in German Vineyards. Plant Dis..

[75] Rasool S., Naz S., Rowhani A., Golino D.A., Westrick N.M., Farrar K.D., Al Rwahnih M. (2017). First Report of Grapevine Pinot Gris Virus Infecting Grapevine in Pakistan. Plant Dis..

[76] Ruiz-García A.B., Olmos A. (2017). First Report of Grapevine Pinot Gris Virus in Grapevine in Spain. Plant Dis..

[77] Fajardo T.V.M., Eiras M., Nickel O. (2017). First Report of Grapevine Pinot Gris Virus Infecting Grapevine in Brazil. Australas. Plant Dis. Notes.

[78] Zamorano A., Medina G., Fernández C., Cui W., Quiroga N., Fiore N. (2019). First Report of Grapevine Pinot Gris Virus in Grapevine in Chile. Plant Dis..

[79] Abou Kubaa R., Choueiri E., Jreijiri F., El Khoury Y., Saldarelli P. (2020). First Report of Grapevine Pinot Gris Virus in Lebanon and the Middle East. J. Plant Pathol..

[80] Abou Kubaa R., Lanotte P., Saldarelli P. (2019). First Report of Grapevine Pinot Gris Virus in Grapevine in Moldavia. J. Plant Pathol..

[81] Casati P., Maghradze D., Quaglino F., Ravasio A., Failla O., Bianco P.A. (2015). First Report of Grapevine Pinot Gris Virus in Georgia. J. Plant Pathol..

[82] Tokhmechi K., Koolivand D. (2020). First Report of Grapevine Pinot Gris Virus Infecting Grapevine in Iran. J. Plant Pathol..

[83] Wu Q., Habili N. (2017). The Recent Importation of Grapevine Pinot Gris Virus into Australia. Virus Genes.

[84] Eichmeier A., Peňázová E., Pavelková R., Mynarzová Z., Saldarelli P. (2016). Detection of Grapevine Pinot Gris Virus in Certified Grapevine Stocks in Moravia, Czech Republic. J. Plant Pathol..

[85] Bertazzon N., Rahali M., Angelini E., Crespan M., Migliaro D. (2020). First Report of Grapevine Pinot Gris Virus Infecting Grapevine in Algeria. Plant Dis..

[86] Silva G., Lecourt J., Clover G.R.G., Seal S.E. (2018). First Record of *Grapevine Pinot Gris Virus* Infecting *Vitis vinifera* in the United Kingdom. N. Dis. Rep..

[87] Eichmeier A., Penazova E., Nebish A. (2019). First Report of Grapevine Pinot Gris Virus on Grapevines in Armenia. Plant Dis..

[88] Bertazzon N., Angelini E., Signorotto M., Genov N. (2021). First Report of Grapevine Pinot Gris Virus and Grapevine Leafroll-Associated Virus 2 in Bulgarian Vineyards. J. Plant Dis. Prot..

[89] Debat H., Luna F., Moyano S., Zavallo D., Asurmendi S., Gomez-Talquenca S. (2020). First Report of Grapevine Pinot Gris Virus Infecting Grapevine in Argentina. J. Plant Pathol..

[90] Rwahnih M.A., Diaz-Lara A., Arnold K., Cooper M.L., Smith R.J., Zhuang G., Battany M.C., Bettiga L.J., Rowhani A., Golino D. (2021). Incidence and Genetic Diversity of Grapevine Pinot Gris Virus in California. Am. J. Enol. Vitic..

[91] Abe J., Tomoyuki N. (2021). First Report of Grapevine Pinot Gris Virus in Wild Grapevines (*Vitis coignetiae*) in Japan. J. Plant Pathol..

[92] Massart S., Vankerkoven L., Blouin A.G., Nourinejhad Zarghani S., Wetzel T. (2020). First Report of Grapevine Pinot Gris Virus and Grapevine Rupestris Stem Pitting-Associated Virus in Grapevine in Belgium. Plant Dis..

[93] Guta I.-C., Buciumeanu E.-C. (2021). Grapevine Pinot Gris Virus Infecting Grapevines in Romania–Short Communicaiton. Hortic. Sci..

[94] Tarquini G., Zaina G., Ermacola P., De Amicis F., Franco-Orozco B., Loi N., Martini M., Luca Bianchi G., Pagliari L., Firrao G. Agroinoculation of Grapevine Pinot Gris Virus in Tobacco and Grapevine Provides Insights on Viral Pathogenesis. https://journals.plos.org/plosone/article?id=10.1371/journal.pone.0214010.

[95] Sabanadzovic S., Aboughanem-Sabanadzovic N., Martelli G.P., Meng B., Martelli G.P., Golino D.A., Fuchs M. (2017). Grapevine Fleck and Similar Viruses. Grapevine Viruses: Molecular Biology, Diagnostics and Management.

[96] Crnogorac A., Gašpar M., Davino S., Mandić A., Matić S. (2020). First Report of Grapevine Fleck Virus in Vineyards of Bosnia and Herzegovina. J. Plant Pathol..

[97] Kanuya E., Clayton L.A., Naidu R.A., Karasev A.V. (2012). First Report of Grapevine Fleck Virus in Idaho Grapevines. Plant Dis..

[98] Poojari S., Lowery T., Rott M., Schmidt A.-M., DeLury N., Boulé J., Úrbez-Torres J.R. (2016). First Report and Prevalence of Grapevine Fleck Virus in Grapevines (*Vitis vinifera*) in Canada. Plant Dis..

[99] Kostadinovska E., Mitrev S., Bianco P.A., Casati P., Bulgari D. (2014). First Report of Grapevine Virus A and Grapevine Fleck Virus in the Former Yugoslav Republic of Macedonia. Plant Dis..

[100] Jo Y., Song M.K., Choi H., Park J.S., Lee J.W., Cho W.K. (2017). First Report of Grapevine Fleck Virus and Grapevine Virus E in Grapevine in Korea. Plant Dis..

[101] Immanuel T.M., Delmiglio C., Ward L.I., Denton J.O., Clover G.R.G. (2015). First Reports of Grapevine Virus A, Grapevine Fleck Virus, and Grapevine Leafroll-Associated Virus 1 in the United Kingdom. Plant Dis..

[102] Ma Y.X., Li S.F., Zhang Z.X. (2017). First Report of Grapevine Rupestris Vein Feathering Virus in an Old Grapevine in China. Plant Dis..

[103] Wu Q., Kehoe M.A., Kinoti W.M., Wang C.P., Rinaldo A., Tyerman S., Habili N., Constable F.E. (2021). First Report of Grapevine Rupestris Vein Feathering Virus in Grapevine in Australia. Plant Dis..

[104] Xiao H., Meng B. (2016). First Report of Grapevine Asteroid Mosaic-Associated Virus and Grapevine Rupestris Vein Feathering Virus in Grapevines in Canada. Plant Dis..

[105] Mahmood M., Gentili A., Naz S., Faggioli F. (2019). First Report of Grapevine Rupestris Vein Feathering Virus in Pakistan. J. Plant Pathol..

[106] Khalili M., Zarghani S.N., Massart S., Dizadji A., Olmos A., Wetzel T., Ruiz-García A.B. (2020). First Report of Grapevine Rupestris Vein Feathering Virus in Grapevine in Iran. J. Plant Pathol..

[107] Aoki Y., Suzuki S. (2021). First Report of Grapevine Rupestris Vein Feathering Virus in *Vitis vinifera* L. from Japan. Plant Dis..

[108] Cho I.S., Chung B.N., Hammond J., Moon J.S., Lim H.S. (2018). First Report of Grapevine Rupestris Vein Feathering Virus Infecting Grapevines in Korea. Plant Dis..

[109] Mekuria T.A., Naidu R.A. (2010). First Report of Grapevine Virus Sequences Highly Similar to Grapevine Syrah Virus-1 from Washington Vineyards. Plant Dis..

[110] Engel E.A., Rivera P.A., Valenzuela P.D.T. (2010). First Report of Grapevine Syrah Virus-1 in Chilean Grapevines. Plant Dis..

[111] Ahmed I., Fan X.D., Zhang Z.P., Ren F., Hu G.J., Li Z.N., Khaskheli M.I., Dong Y.F. (2017). First Report of Grapevine Syrah Virus-1 in Grapevines in China. Plant Dis..

[112] Ruiz-García A.B., Sabaté J., Lloria O., Laviña A., Batlle A., Olmos A. (2017). First Report of Grapevine Syrah Virus-1 in Grapevine in Spain. Plant Dis..

[113] Cho I.S., Yang C.Y., Kwon S.J., Yoon J.Y., Kim D.H., Choi G.S., Hammond J., Moon J.S., Lim H.S. (2019). First Report of Grapevine Syrah Virus 1 Infecting Grapevines in Korea. Plant Dis..

[114] Czotter N., Szabó E., Molnar J., Kocsis L., Deák T., Bisztray G., Tusnády G.E., Burgyán J., Várallyay É. (2015). First Description of Grapevine Syrah Virus 1 in Vineyards of Hungary. J. Plant Pathol..

[115] Oosthuizen K., Coetzee B., Maree H.J., Burger J.T. (2016). First Report of Grapevine Syrah Virus 1 in South African Grapevines. Plant Dis..

[116] Caglayan K., Gazel M., Kocabag H.D. (2017). First Report of Grapevine Syrah Virus 1 in Grapevine in Turkey. J. Plant Pathol..

[117] Glasa M., Predajňa L., Šoltys K., Sabanadzovic S., Olmos A. (2015). Detection and Molecular Characterisation of Grapevine Syrah Virus-1 Isolates from Central Europe. Virus Genes.

[118] Mavrič Pleško I., Lamovšek J., Lešnik A., Viršček Marn M. (2020). Raspberry Bushy Dwarf Virus in Slovenia—Geographic Distribution, Genetic Diversity and Population Structure. Eur. J. Plant Pathol..

[119] Mavrič I., Marn M.V., Koron D., Žežlina I. (2003). First Report of Raspberry Bushy Dwarf Virus on Red Raspberry and Grapevine in Slovenia. Plant Dis..

[120] Pleško I.M., Marn M.V., Nyerges K., Lázár J. (2012). First Report of Raspberry Bushy Dwarf Virus Infecting Grapevine in Hungary. Plant Dis..

[121] Jevremovic D., Paunovic S. (2011). Raspberry Bushy Dwarf Virus: A Grapevine Pathogen in Serbia. Pesticidi i Fitomedicina.

[122] Polivka H., Staub U., Gross H.J. (1996). Variation of Viroid Profiles in Individual Grapevine Plants: Novel Grapevine Yellow Speckle Viroid 1 Mutants Show Alterations of Hairpin I. J. Gen. Virol..

[123] Di Serio F., Izadpanah K., Hajizadeh M., Navarro B., Meng B., Martelli G.P., Golino D.A., Fuchs M. (2017). Viroids Infecting the Grapevine. Grapevine Viruses: Molecular Biology, Diagnostics and Management.

[124] Cañizares M.C., Marcos J.F., Pallás V. (1999). Molecular Characterization of an Almond Isolate of Hop Stunt Viroid (HSVd) and Conditions for Eliminating Spurious Hybridization in Its Diagnosis in Almond Samples. Eur. J. Plant Pathol..

[125] Marquez-Molins J., Gomez G., Pallas V. (2021). Hop Stunt Viroid: A Polyphagous Pathogenic RNA That Has Shed Light on Viroid–Host Interactions. Mol. Plant Pathol..

[126] Zhang Z., Zhou Y., Guo R., Mu L., Yang Y., Li S., Wang H. (2012). Molecular Characterization of Chinese Hop Stunt Viroid Isolates Reveals a New Phylogenetic Group and Possible Cross Transmission between Grapevine and Stone Fruits. Eur. J. Plant Pathol..

[127] Hataya T., Tsushima T., Sano T., Hadidi A., Flores R., Randles J.W., Palukaitis P. (2017). Chapter 19—Hop Stunt Viroid. Viroids and Satellites.

[128] Rezaian M.A. (1990). Australian Grapevine Viroid—Evidence for Extensive Recombination between Viroids. Nucleic Acids Res..

[129] Elleuch A., Fakhfakh H., Pelchat M., Landry P., Marrakchi M., Perreault J.-P. (2002). Sequencing of Australian Grapevine Viroid and Yellow Speckle Viroid Isolated from a Tunisian Grapevine without Passage in an Indicator Plant. Eur. J. Plant Pathol..

[130] Elleuch A., Marrakchi M., Perreault J.P., Fakhfakh H. (2003). First Report of Australian Grapevine Viroid from the Mediterranean Region. J. Plant Pathol..

[131] Jiang D., Peng S., Wu Z., Cheng Z., Li S. (2009). Genetic Diversity and Phylogenetic Analysis of Australian Grapevine Viroid (AGVd) Isolated from Different Grapevines in China. Virus Genes.

[132] Zaki-Aghl M., Izadpanah K., Niazi A., Behjatnia A., Afsharifar A. (2013). Molecular and Biological Characterization of the Iranian Isolate of the Australian Grapevine Viroid. J. Agric. Sci. Technol..

[133] Gambino G., Navarro B., La Notte P., Mannini F., Di Serio F. Survey on Viroids Infecting Grapevine in Italy: Identification and Characterization of Australian Grapevine Viroid and Grapevine Yellow Speckle Viroid 2. https://link.springer.com/article/10.1007%2Fs10658-014-0458-x.

[134] Adkar-Purushothama C.R., Kanchepalli P.R., Yanjarappa S.M., Zhang Z., Sano T. (2014). Detection, Distribution, and Genetic Diversity of Australian Grapevine Viroid in Grapevines in India. Virus Genes.

[135] Saengmanee P., Burns P., Wetzel T. (2018). First Report of Australian Grapevine Viroid in Grapevine in Thailand. Plant Dis..

[136] Kryovrysanaki N., Katsarou K., Olmos A., Ruiz-García A.B., Kalantidis K., Pappi P. (2021). First Report of Australian Grapevine Viroid in Grapevine in Greece. J. Plant Pathol..

[137] Barbara D.J., Morton A., Ramcharan S., Cole I.W., Phillips A., Knight V.H. (2001). Occurrence and Distribution of Raspberry Bushy Dwarf Virus in Commercial Rubus Plantations in England and Wales. Plant Pathol..

[138] Čepin U., Gutiérrez-Aguirre I., Ravnikar M., Pompe-Novak M. (2016). Frequency of Occurrence and Genetic Variability of Grapevine Fanleaf Virus Satellite RNA. Plant Pathol..

[139] Cigsar I., Digiaro M., Gokalp K., Ghanem-Sabanadzovic N.A., Stradis A.D., Boscia D., Martell G.P. (2003). Grapevine deformation virus, a novel nepovirus from Turkey. J. Plant Pathol..

[140] Daldoul S., Massart S., Ruiz-García A.B., Olmos A., Wetzel T. (2018). First Report of Grapevine Rupestris Vein Feathering Virus in Grapevine in Germany. Plant Dis..

[141] Gambino G., Gribaudo I. (2006). Simultaneous Detection of Nine Grapevine Viruses by Multiplex Reverse Transcription-Polymerase Chain Reaction with Coamplification of a Plant RNA as Internal Control. Phytopathology.

[142] Glasa M., Predajňa L., Komínek P., Nagyová A., Candresse T., Olmos A. (2014). Molecular Characterization of Divergent Grapevine Pinot Gris Virus Isolates and Their Detection in Slovak and Czech Grapevines. Arch. Virol..

[143] Gottula J., Lapato D., Cantilina K., Saito S., Bartlett B., Fuchs M. (2013). Genetic Variability, Evolution, and Biological Effects of Grapevine Fanleaf Virus Satellite RNAs. Phytopathology.

[144] Hajizadeh M., Navarro B., Bashir N.S., Torchetti E.M., Di Serio F. (2012). Development and Validation of a Multiplex RT-PCR Method for the Simultaneous Detection of Five Grapevine Viroids. Sci. Direct.

[145] Jiang D., Zhang Z., Wu Z., Guo R., Wang H., Fan P., Li S. (2009). Molecular Characterization of Grapevine Yellow Speckle Viroid-2 (GYSVd-2). Virus Genes.

[146] Osman F., Rowhani A. (2006). Application of a Spotting Sample Preparation Technique for the Detection of Pathogens in Woody Plants by RT-PCR and Real-Time PCR (TaqMan). Sci. Direct.

[147] Padilla C.V., Cretazzo E., Hita I., López N., Padilla V., Velasco L. (2010). First Report of Grapevine Leafroll-Associated Virus 5 in Spain. Plant Dis..

[148] Rowhani A., Golino D.A., Uyemoto J.K., Zhang Y.P. Isolation and Partial Characterization of Two New Viruses from Grapevine. Proceedings of the 13th Meeting of the International Council for the Study of Viruses and Virus-Like Diseases of the Grapevine (ICVG).

[149] Sano T., Mimura R., Ohshima K. (2001). Phylogenetic Analysis of Hop and Grapevine Isolates of Hop Stunt Viroid Supports a Grapevine Origin for Hop Stunt Disease. Virus Genes.

[150] Wetzel T., Bassler A., Golino D.A., Uyemoto J.K. (2006). A RT/PCR-Partial Restriction Enzymatic Mapping (PREM) Method for the Molecular Characterisation of the Large Satellite RNAs of Arabis Mosaic Virus Isolates. Sci. Direct.

[151] Cretazzo E., Velasco L. (2017). High-throughput Sequencing Allowed the Completion of the Genome of Grapevine Red Globe Virus and Revealed Recurring Co-infection with Other Tymoviruses in Grapevine. Plant Pathol..

[152] Ghanem-Sabanadzovic N.A., Sabanadzovic S., Castellano M.A., Boscia D., Martelli G.P. (2000). Properties of a New Isolate of Grapevine Leafroll-Associated Virus 2. Vitis.

[153] Naidu R.A., Mekuria T.A. (2010). First Report of Grapevine Fleck Virus from Washington Vineyards. Plant Dis..

[154] Glasa M., Predajňa L., Sihelská N., Šoltys K., Ruiz-García A.B., Olmos A., Wetzel T., Sabanadzovic S. (2018). Grapevine Virus T Is Relatively Widespread in Slovakia and Czech Republic and Genetically Diverse. Virus Genes.

[155] Wetzel T., Jardak R., Meunier L., Ghorbel A., Reustle G.M., Krczal G. (2002). Simultaneous RT/PCR Detection and Differentiation of Arabis Mosaic and Grapevine Fanleaf Nepoviruses in Grapevines with a Single Pair of Primers. J. Virol. Methods.

[156] Reynard J.-S., Brodard J., Dubuis N., Yobregat O., Kominek P., Schumpp O., Schaerer S. (2017). First Report of Grapevine Rupestris Vein Feathering Virus in Swiss Grapevines. Plant Dis..

[157] Minafra A., Hadidi A. (1994). Sensitive Detection of Grapevine Virus A, B, or Leafroll-Associated III from Viruliferous Mealybugs and Infected Tissue by CDNA Amplification. J. Virol. Methods.

