# Supplementary material for: High-Throughput Sequencing of Small RNAs for Diagnostics of Grapevine Viruses and Viroids in Russia
Source: Viruses. 2021 Dec 3;13(12):2432. doi: 10.3390/v13122432 (PMC8709451; doi:10.3390/v13122432)

Library Zs85-SV2_S19

1606 Novichok {GLRaV-3, GFkV, GVT, GPGV, GRVFV, GRSPaV, GSyV-1, GYSVd-2, AGVd}

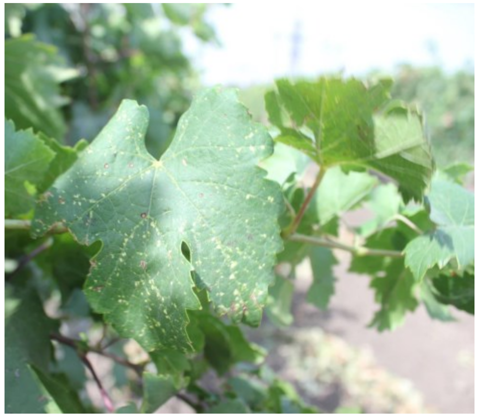
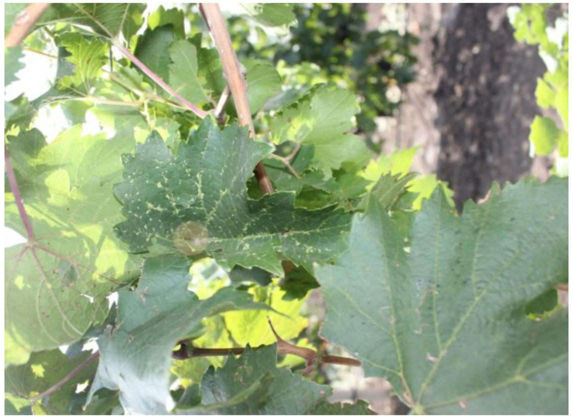
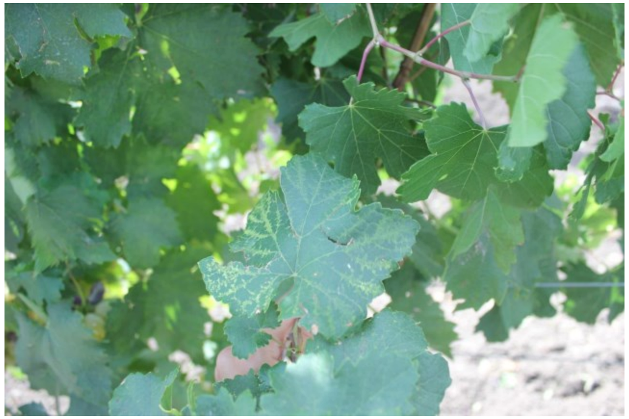

1609 Livia {GLRaV-1, GLRaV-3, GFkV, GVT, GPGV, GRSPaV, GVA, GSyV-1, HSVd}

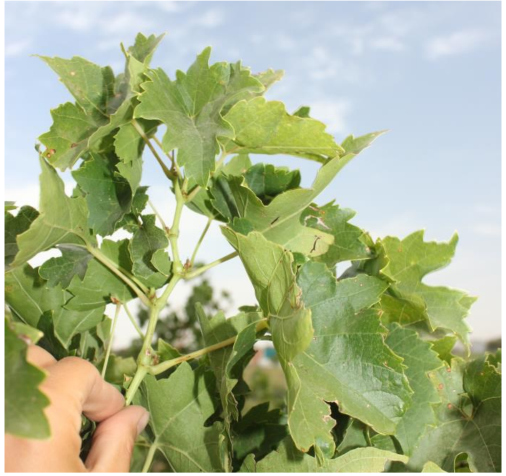
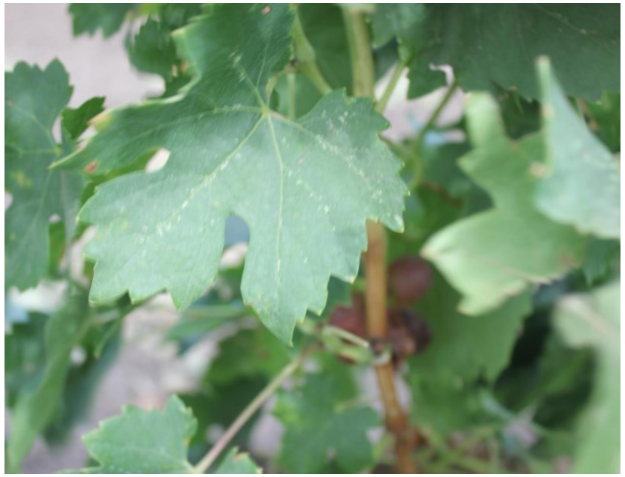
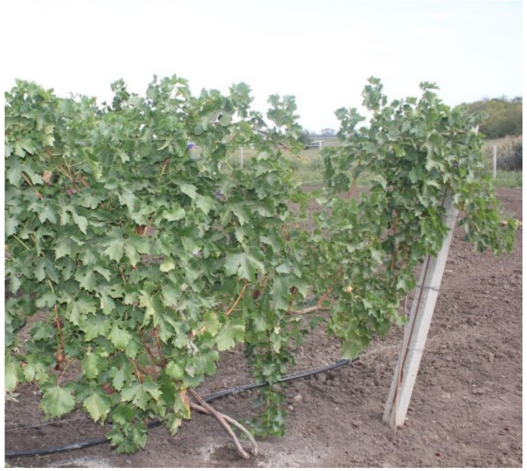

1610 Livia {GLRaV-1, GLRaV-2, GLRaV-3, GFkV, GPGV, GRVFV, GRSPaV, GVA, GSyV-1, HSVd}

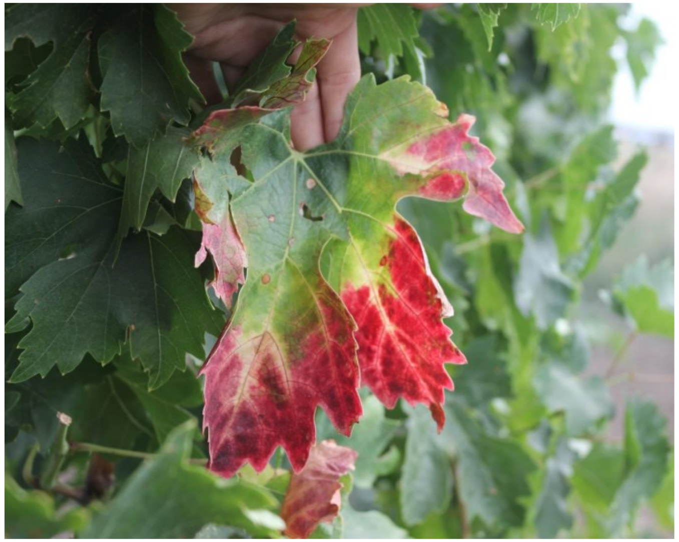
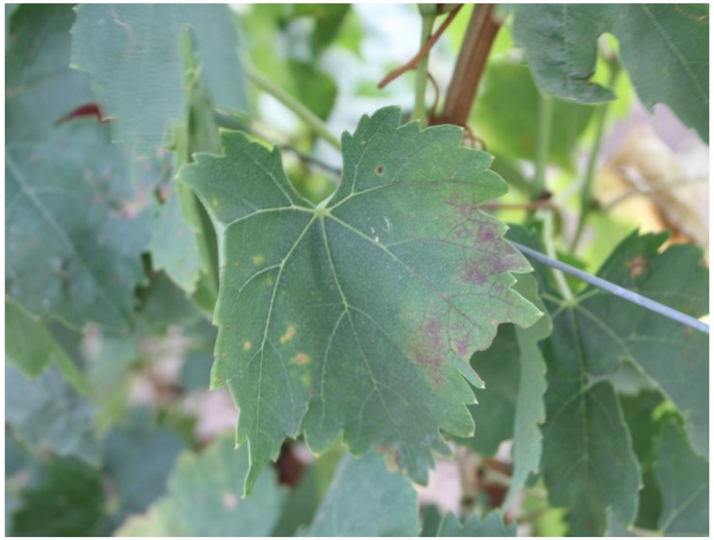
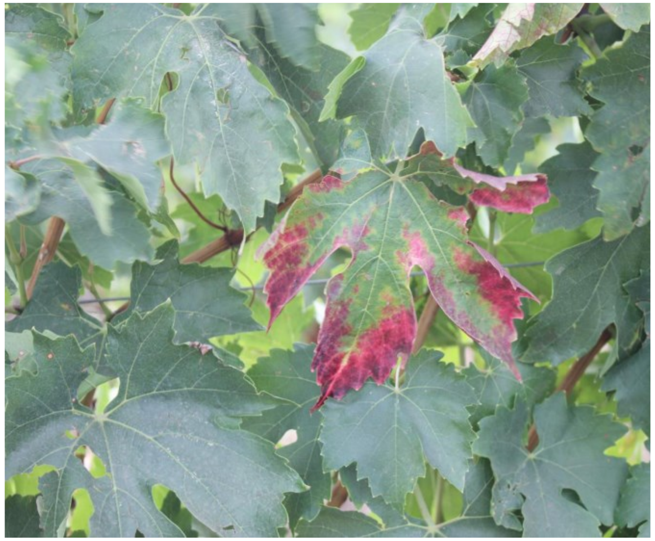

1612 Preobrazhenie {GFkV, GPGV, GRSPaV, GSyV-1, HSVd}

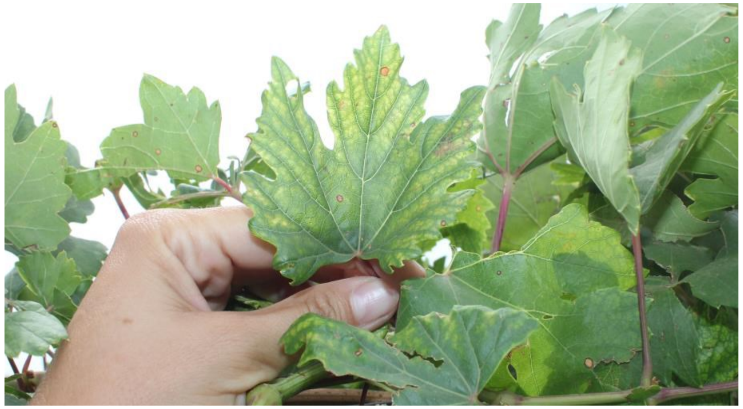
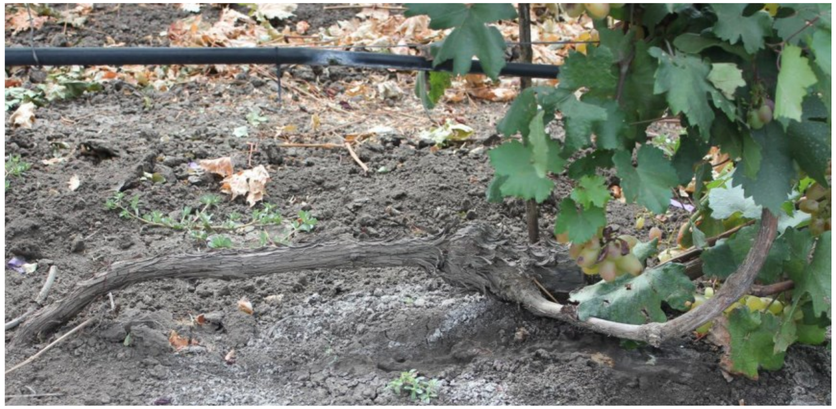
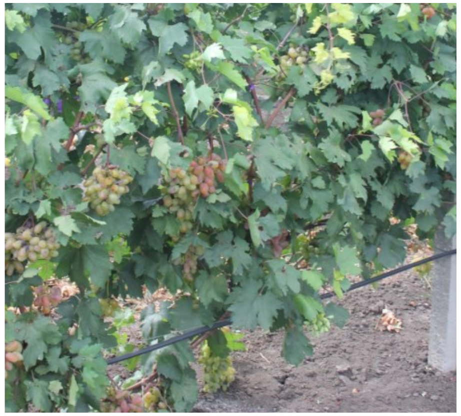

1615 Livia {GLRaV-2, GFkV, GVT, GPGV, GRSPaV, GYSVd-2, AGVd}

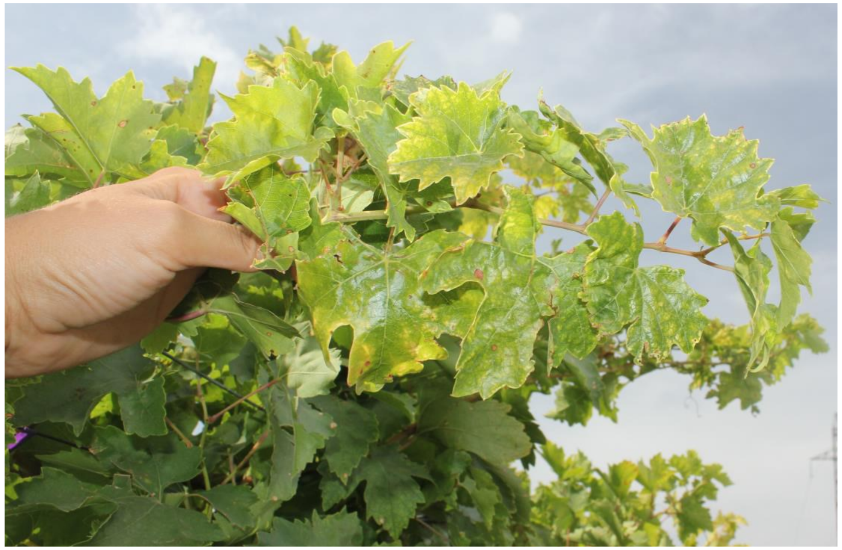
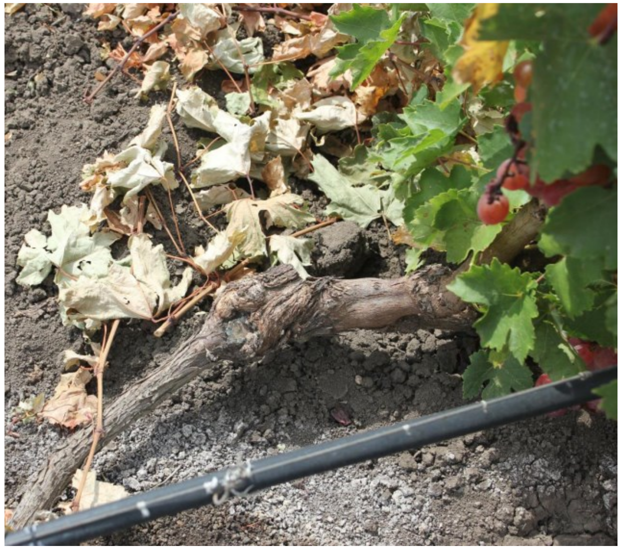
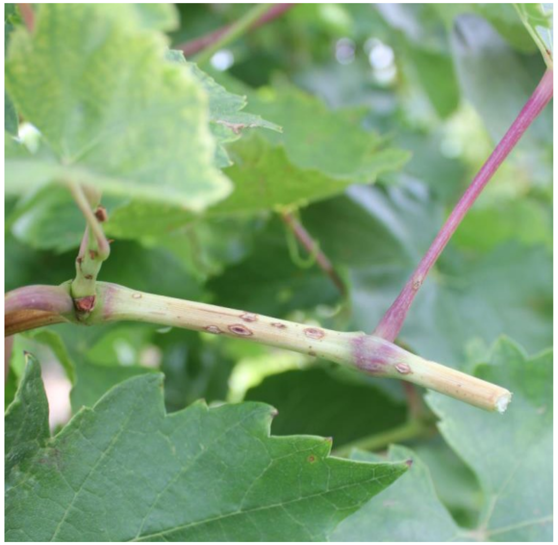

1619 Livia {GLRaV-2, GLRaV-3, GFkV, GPGV, GRSPaV, GSyV-1, GYSVd-1, HSVd}

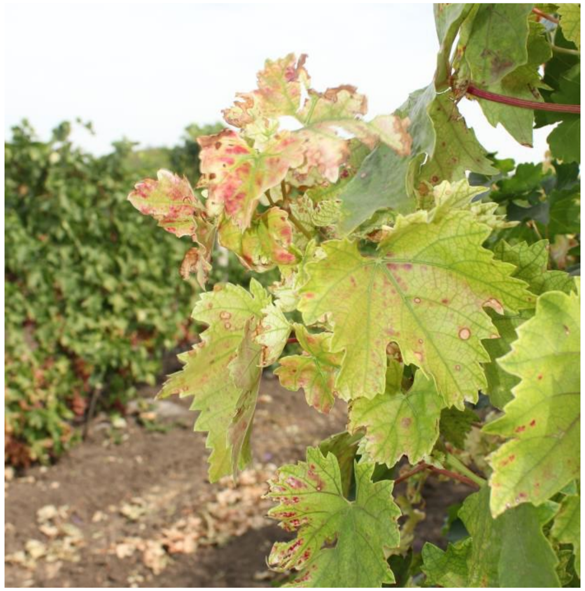
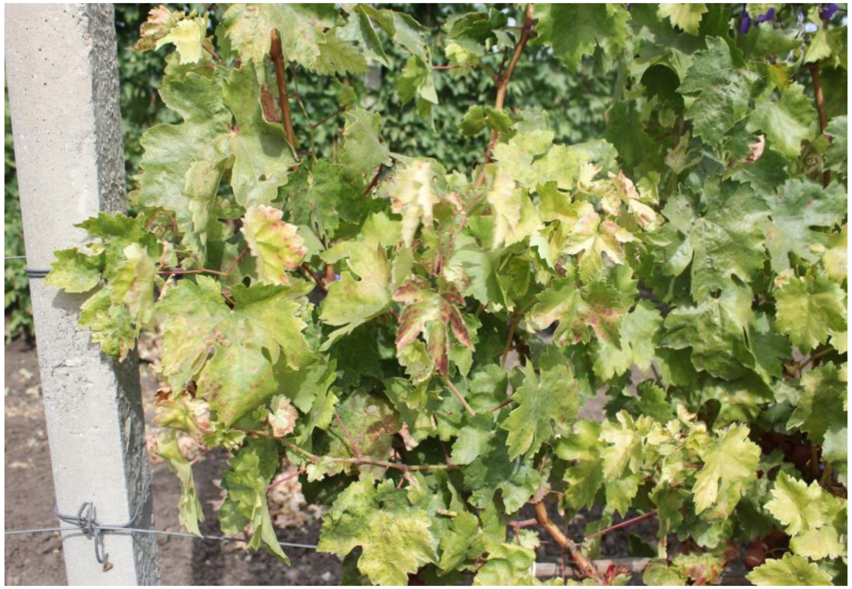
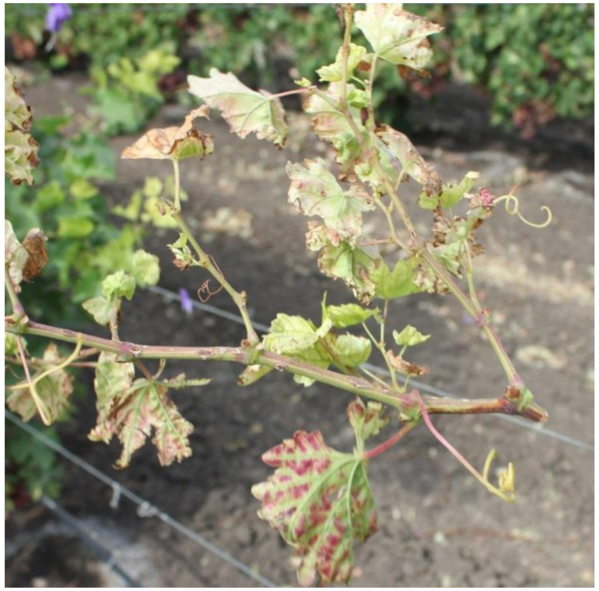

1620 Livia {GLRaV-2, GLRaV-3, GFkV, GPGV, GRVFV, GRSPaV, GSyV-1, GYSVd-1, GYSVd-2, HSVd, AGVd}

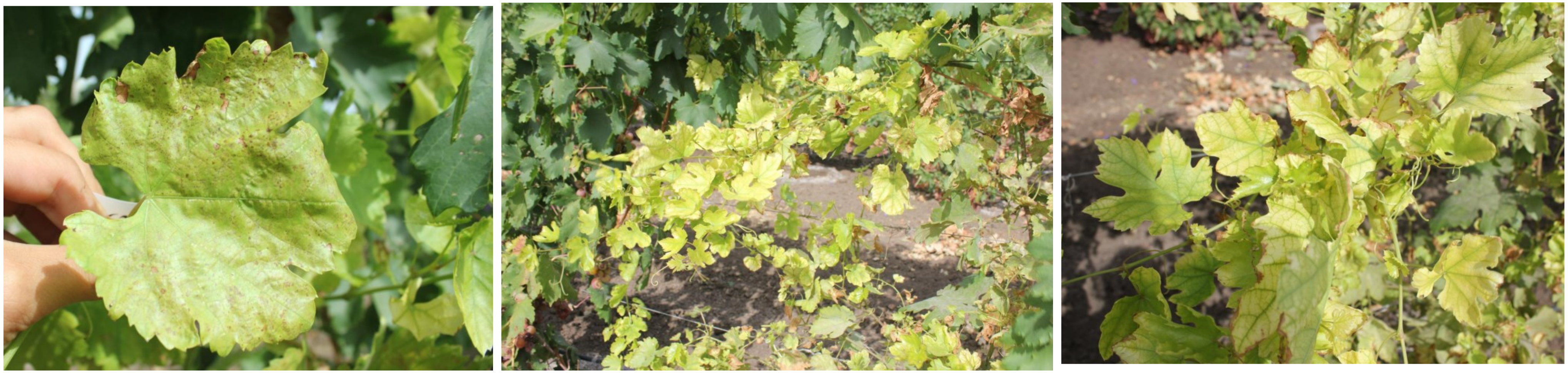

Library Lib-03_S20

263 Krasnostop Zolotovsky {GLRaV-3, GPGV, GRSPaV, GSyV-1, GYSVd-2, HSVd, AGVd}

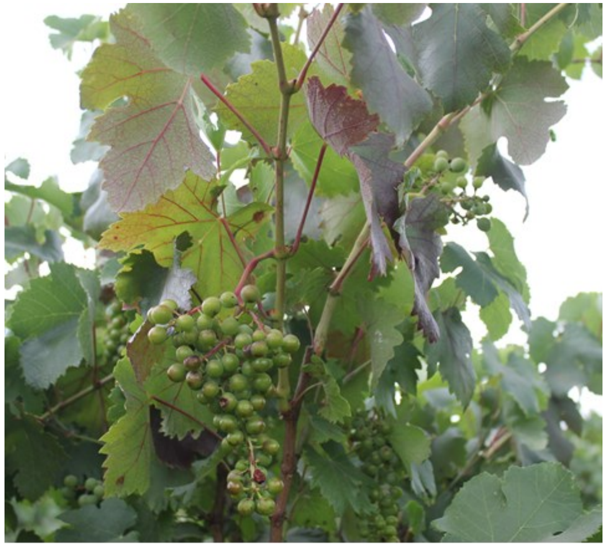
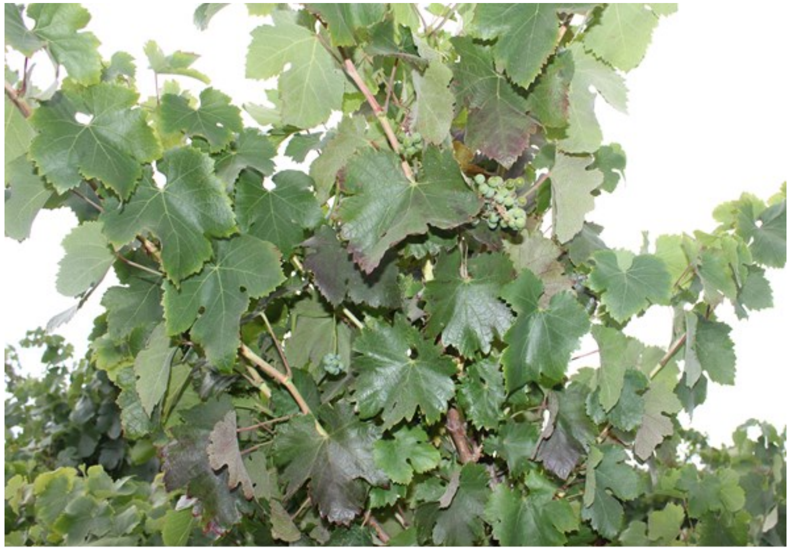
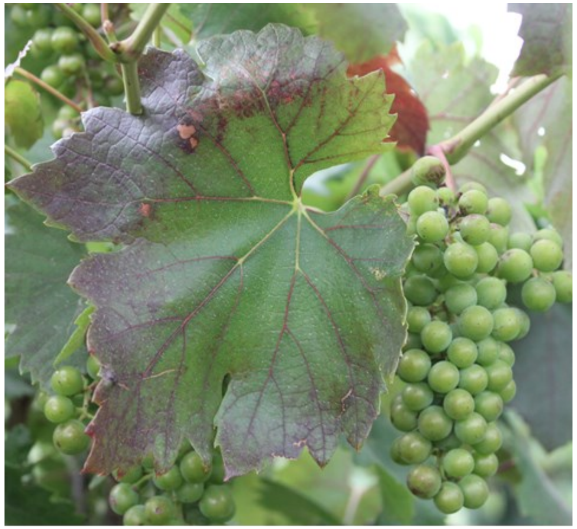

347 Krasnostop Zolotovsky {GFkV, GVT, GPGV, GRSPaV, GSyV-1, GYSVd-1, AGVd}

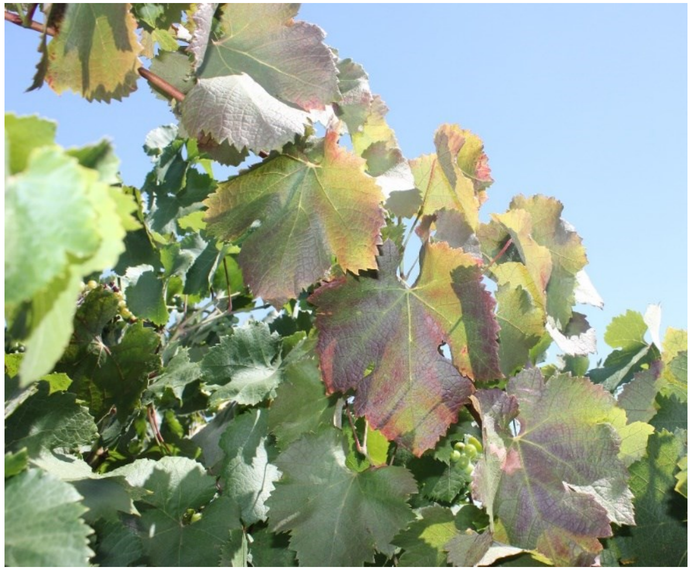
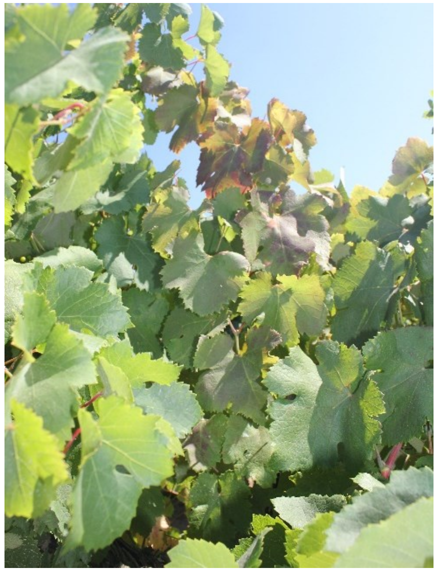
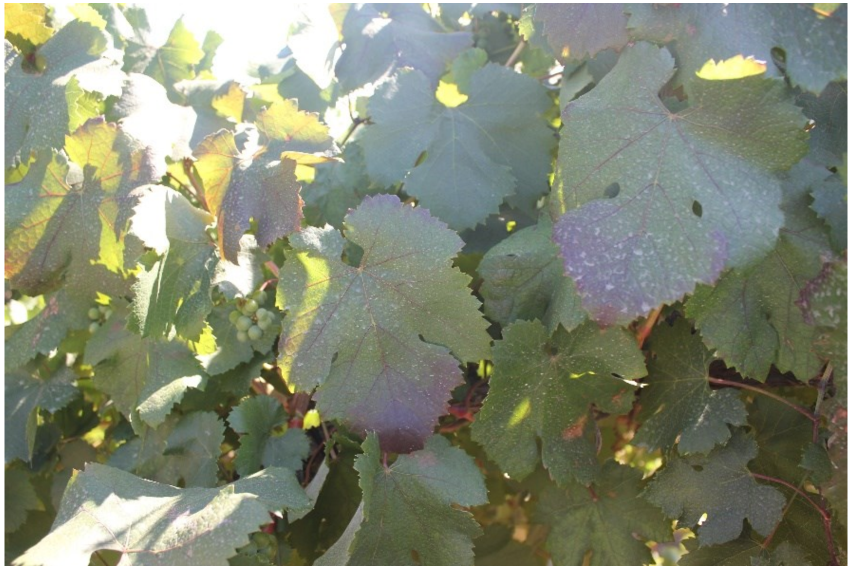

351 Pervenec Magaracha {GFkV, GVT, GPGV, GRSPaV, GYSVd-1, HSVd}

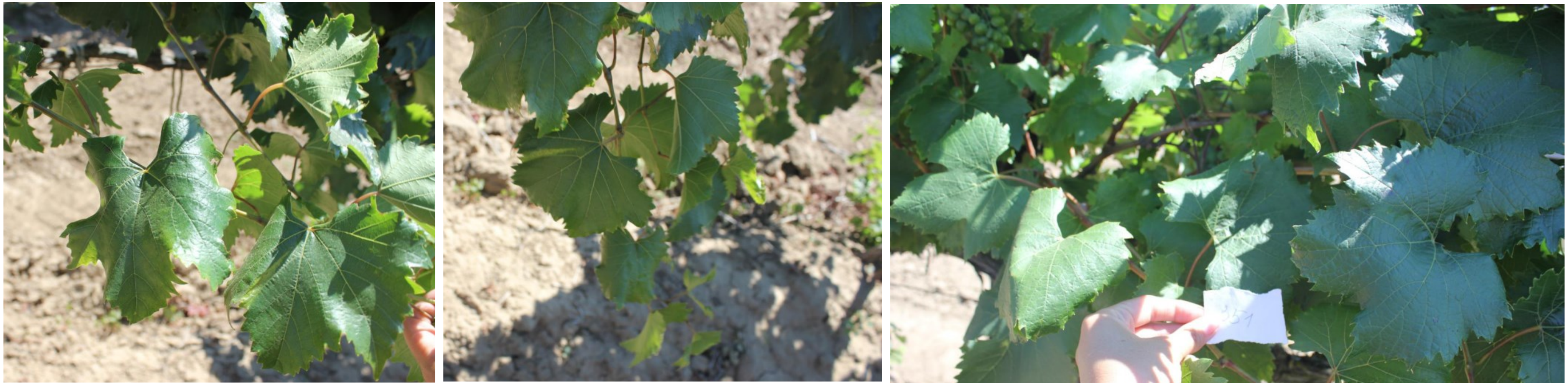

622 Krasnostop Zolotovsky {GFkV, GVT, GPGV, GRSPaV, GSyV-1, GYSVd-2, HSVd}

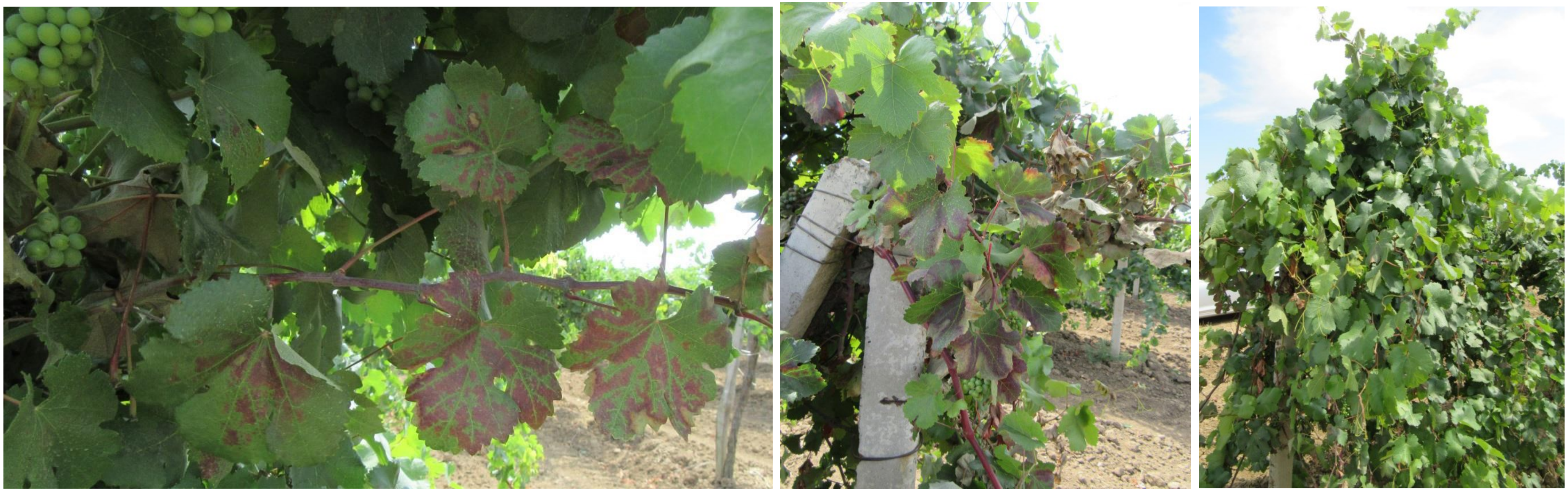

628 Krasnostop Zolotovsky {GLRaV-3, GFkV, GVT, GPGV, GSyV-1, GYSVd-1, HSVd}

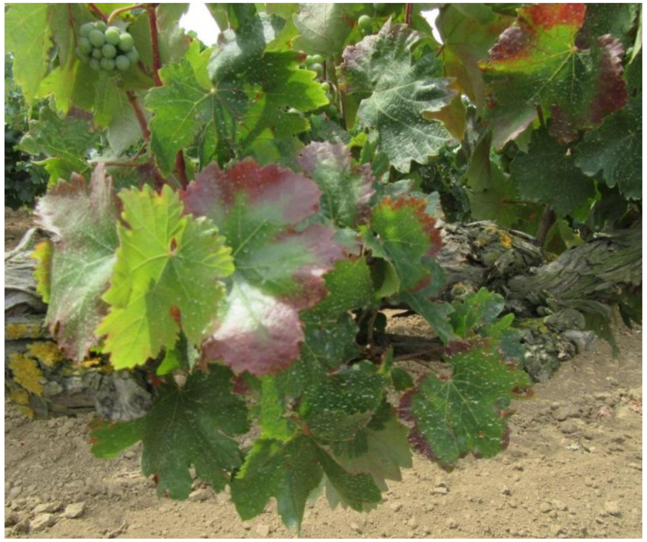

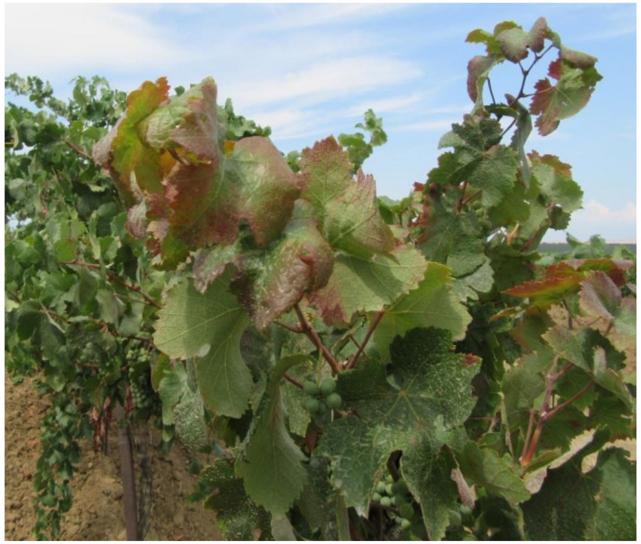

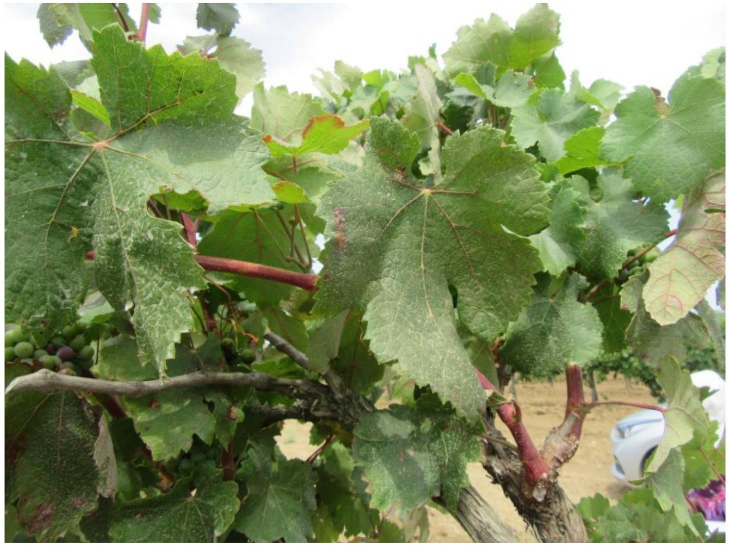

650 Saperavi {GLRaV-4, GFkV, GPGV, GRSPaV, GSyV-1, GYSVd-1, HSVd}

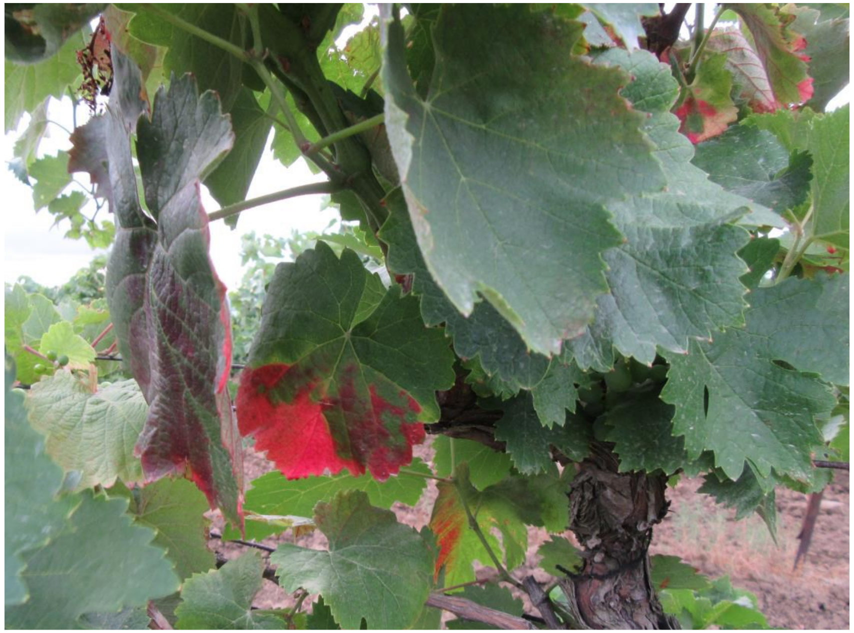

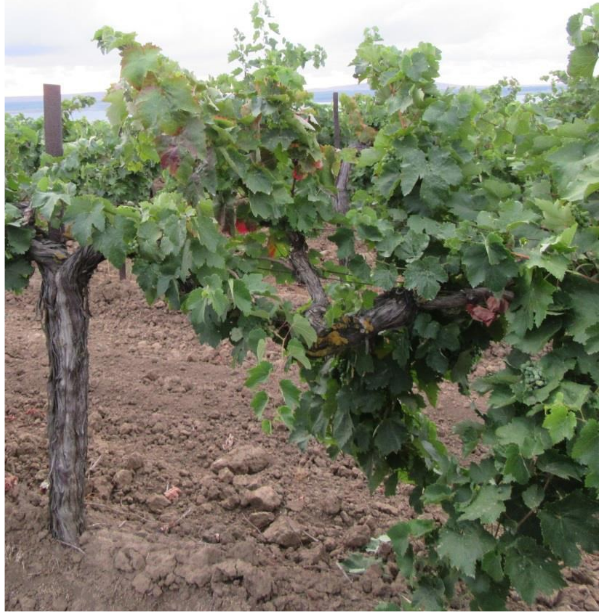

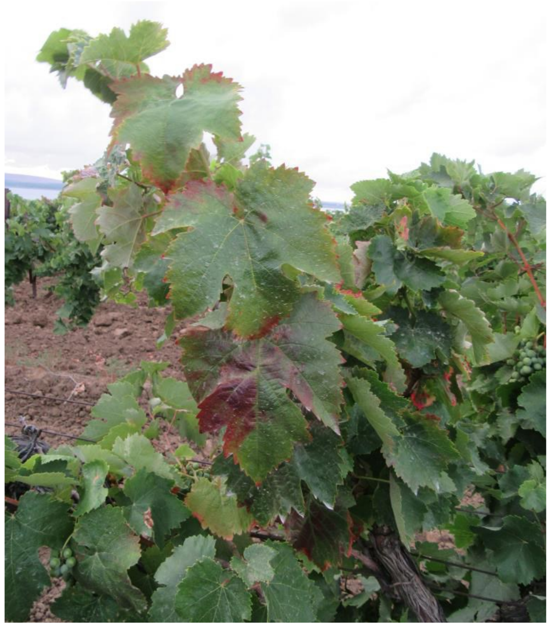

654 Saperavi {GFkV, GVT, GPGV, GRVFV, GRSPaV, GSyV-1, GYSVd-1, HSVd}

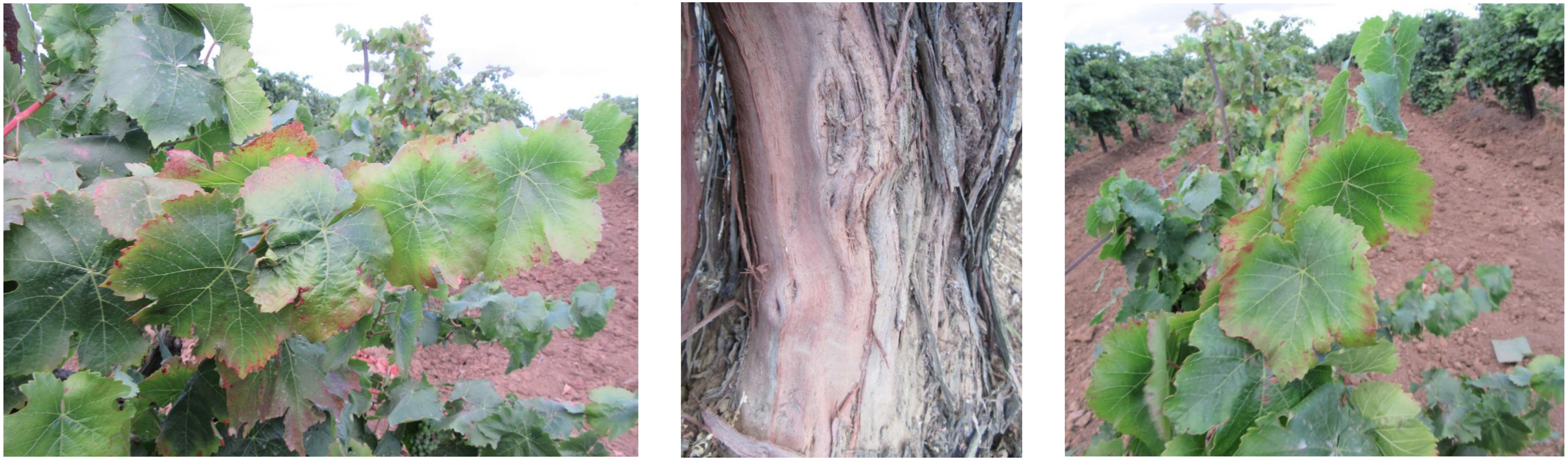

1512 Saperavi {GLRaV-1, GFkV, GVT, GPGV, GRSPaV, GSyV-1, GYSVd-1, HSVd}

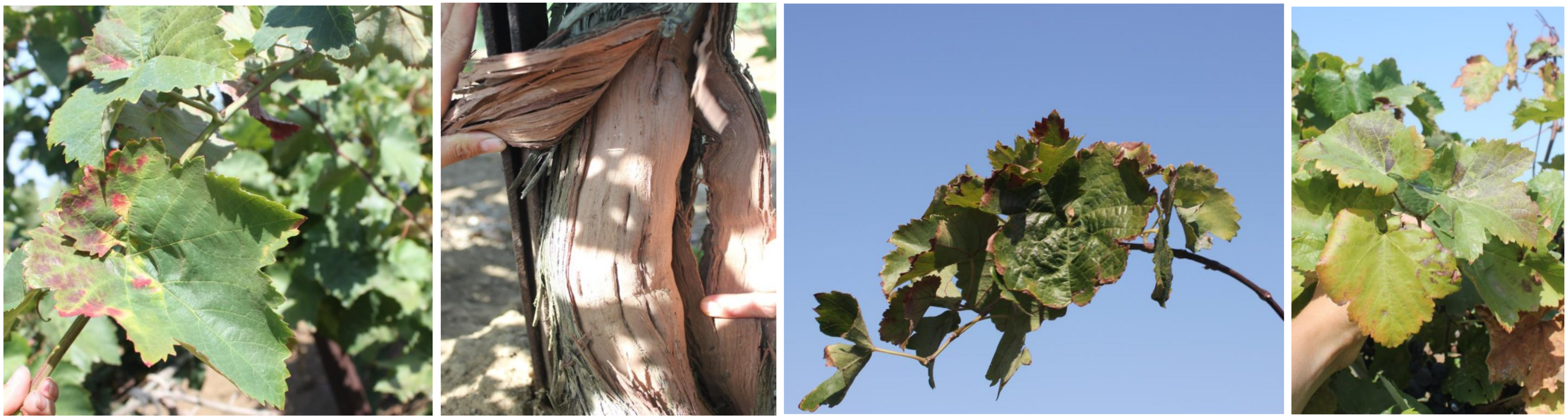

1515 Saperavi {GLRaV-4, GFkV, GVT, GPGV, GRVFV, GRSPaV, GSyV-1, GYSVd-1, HSVd}

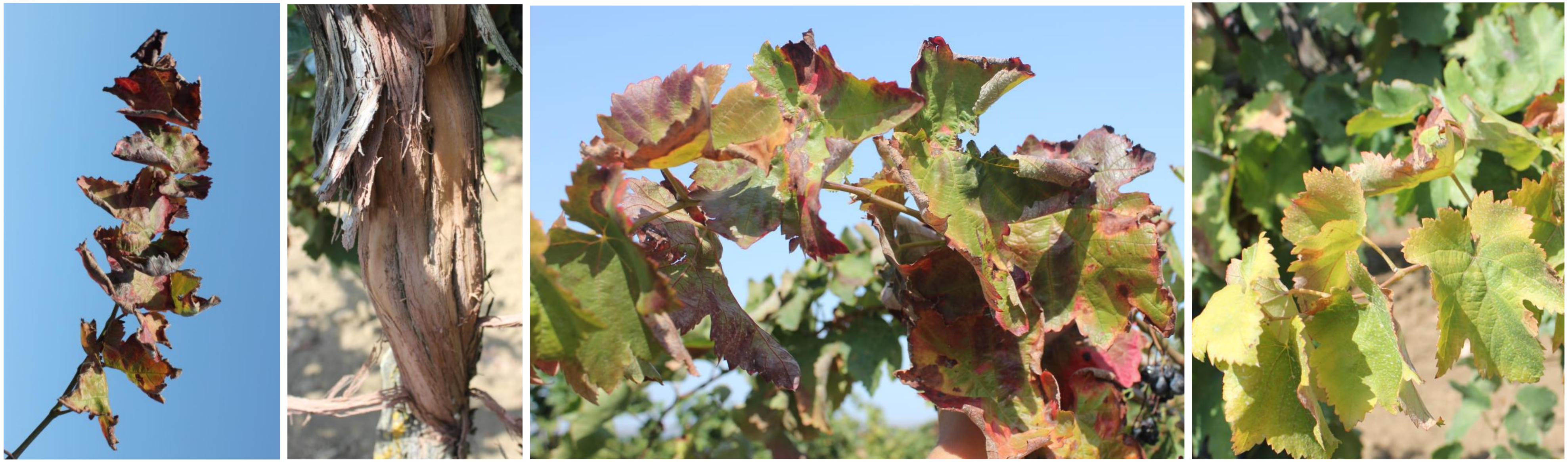

Library Lib-04_S21

289 Chardonnay {GPGV, GRSPaV, GSyV-1, HSVd}

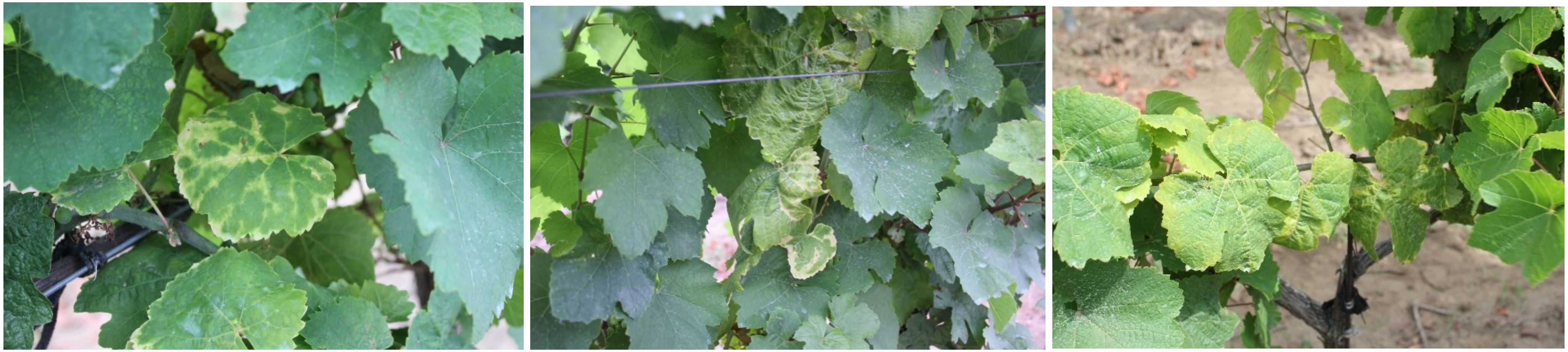

290 Riesling {GPGV}

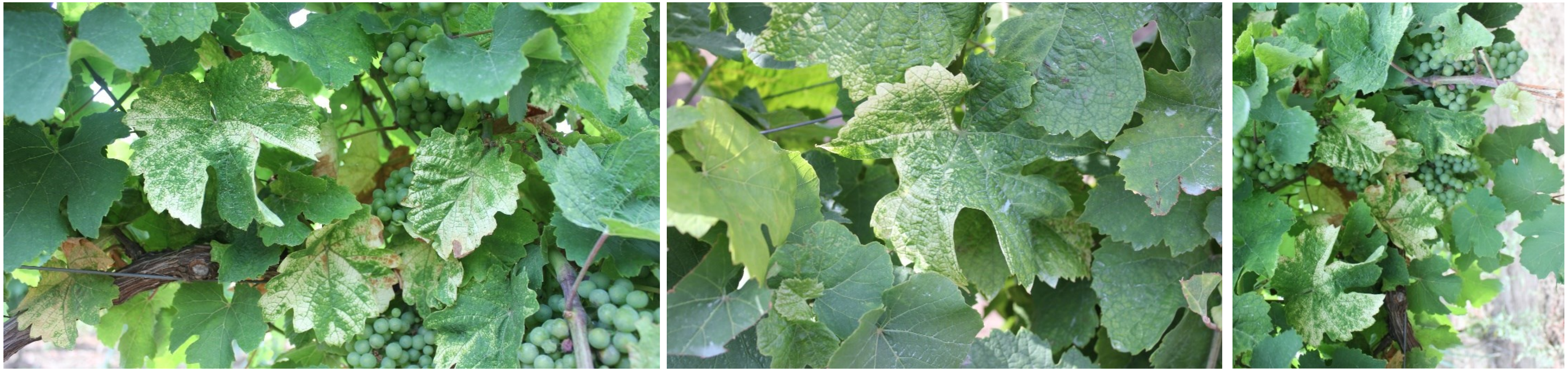

377 Moldova {GFkV, GPGV, GRSPaV, GSyV-1, GFLV, GYSVd-1, HSVd}

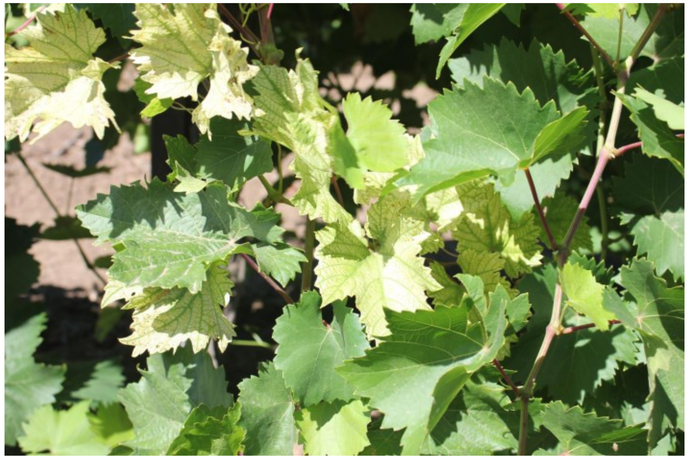

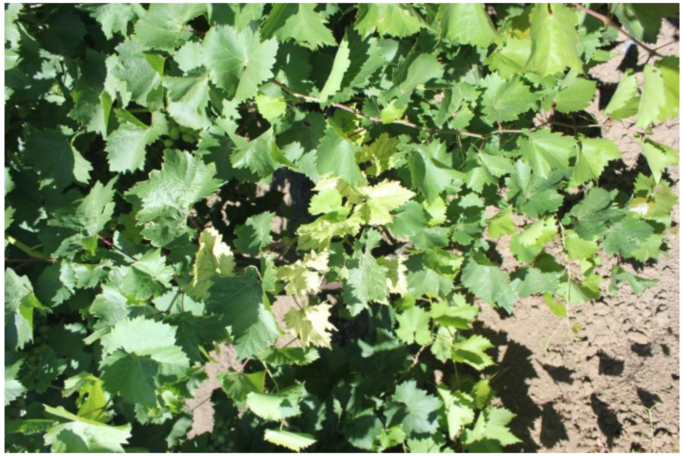

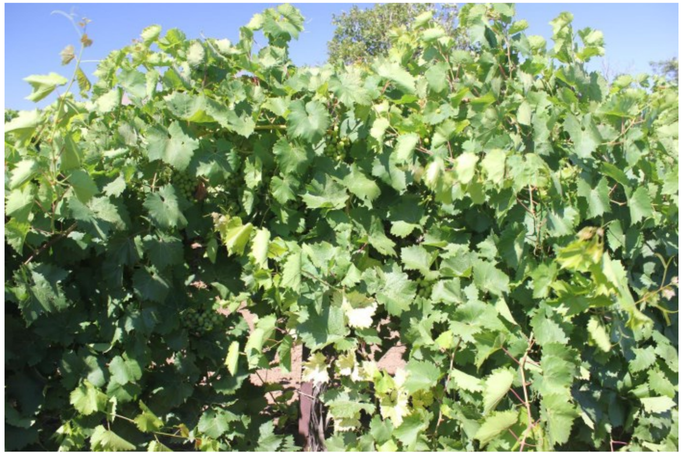

429 Moldova {GLRaV-3, GFkV, GPGV, GRVFV, GRSPaV, GSyV-1, HSVd}

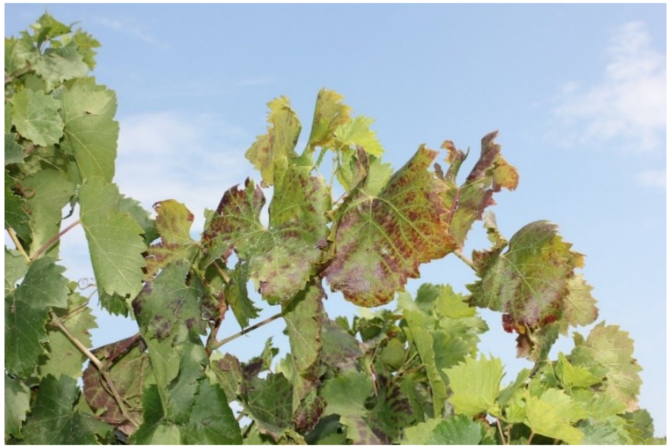

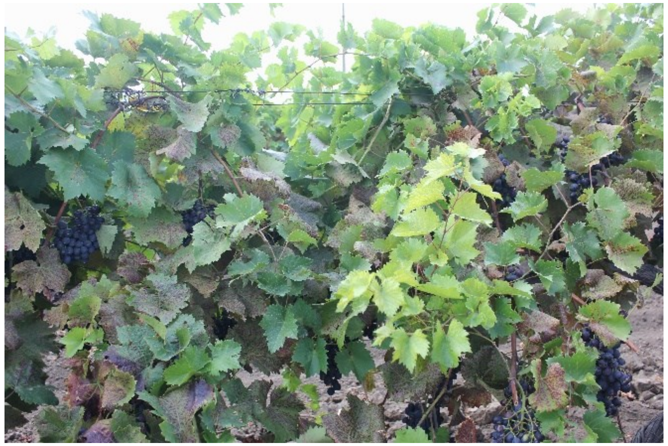

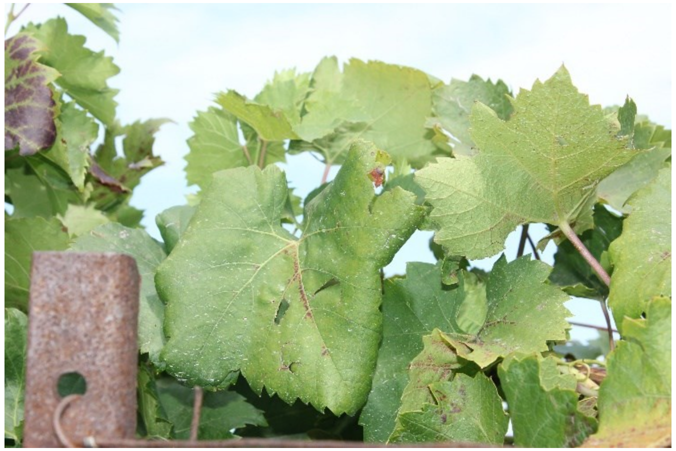

1524 Moldova {GVT, GPGV, GRSPaV, GSyV-1, GYSVd-1, HSVd}

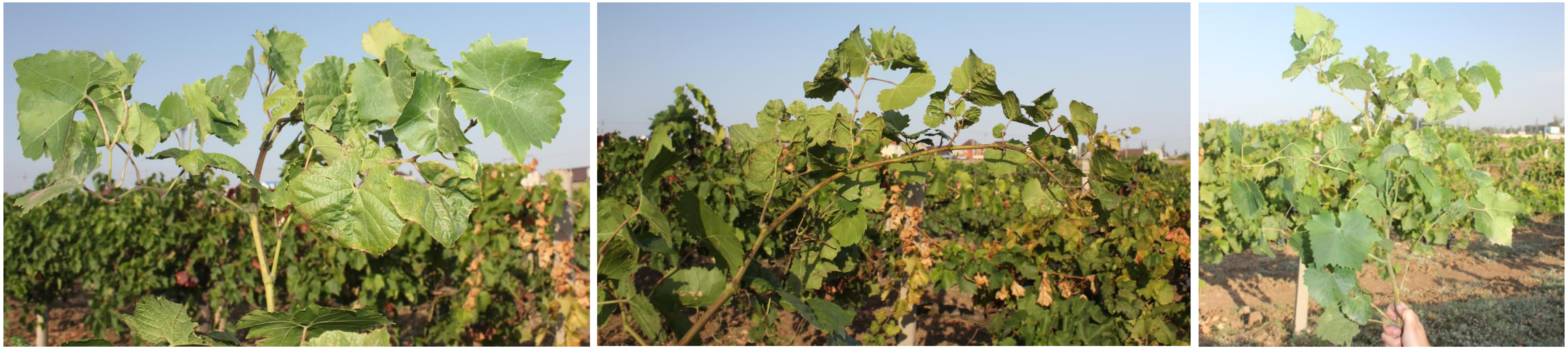

1533 Moldova {GPGV, GRSPaV, GSyV-1, GYSVd-1, HSVd}

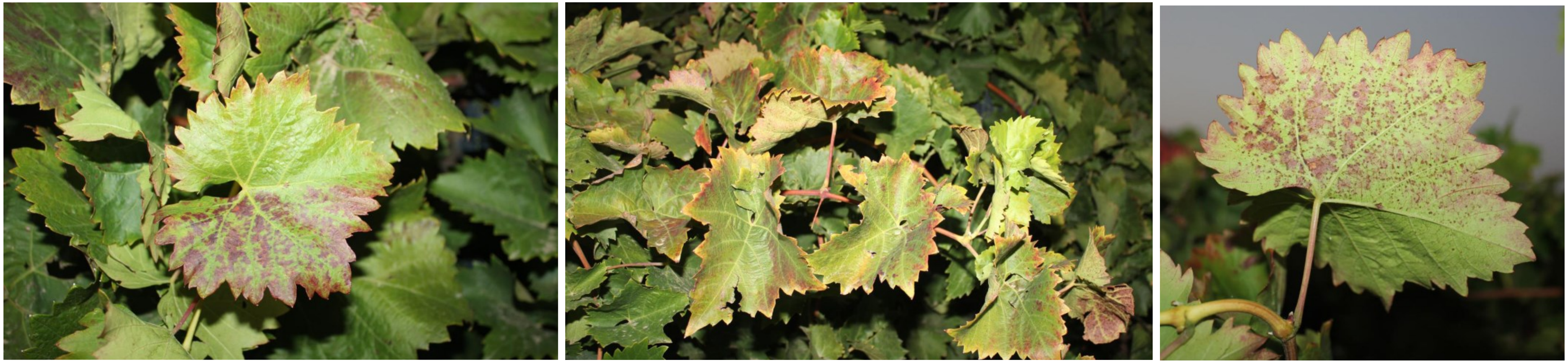

588 Chardonnay {GRSPaV, GSyV-1, GYSVd-1, GYSVd-2, HSVd}

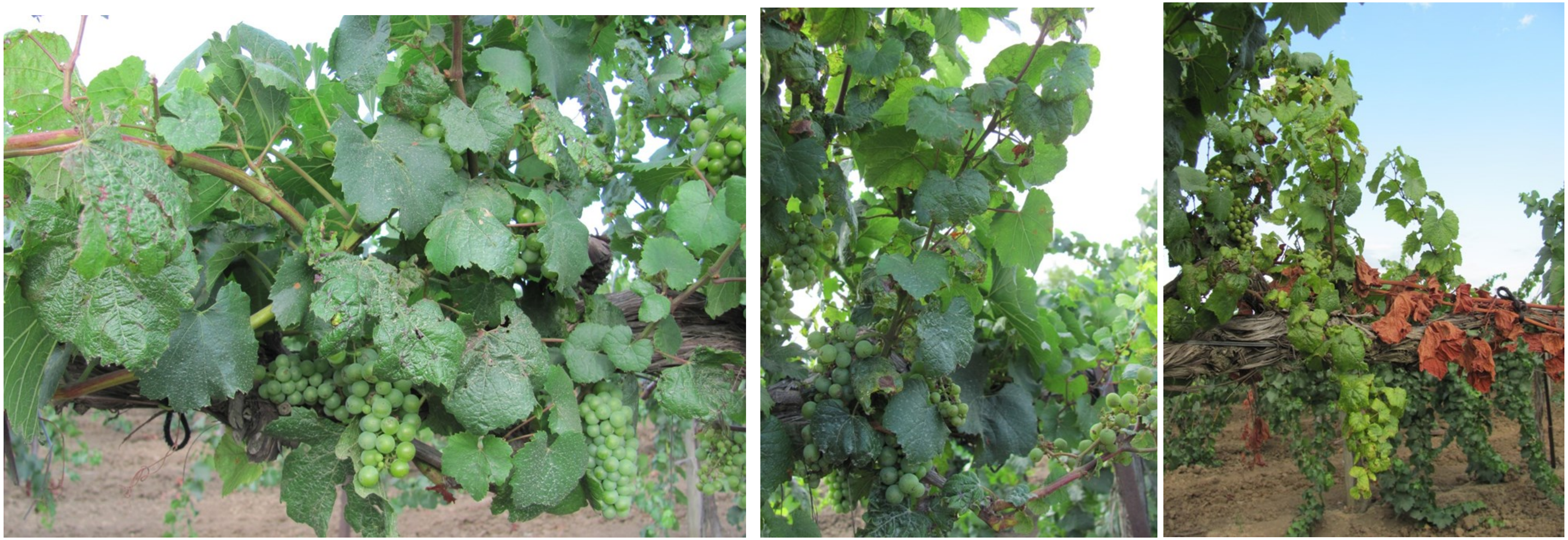

Library **Lib-05_S22**

267 Rkatsiteli {GFkV, GPGV, GRSPaV, GSyV-1, GYSVd-2}

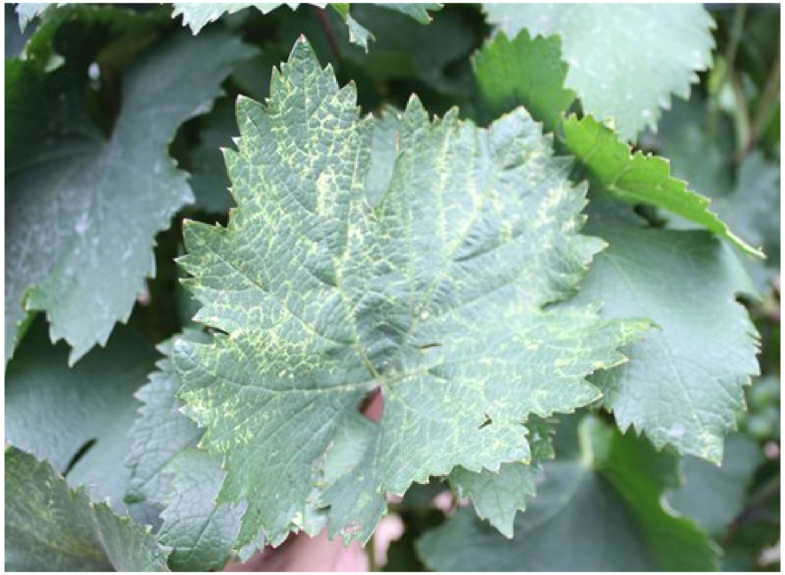
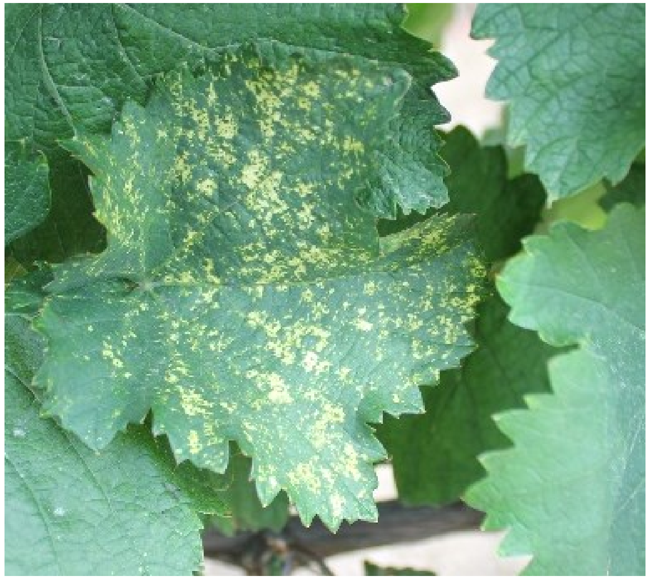
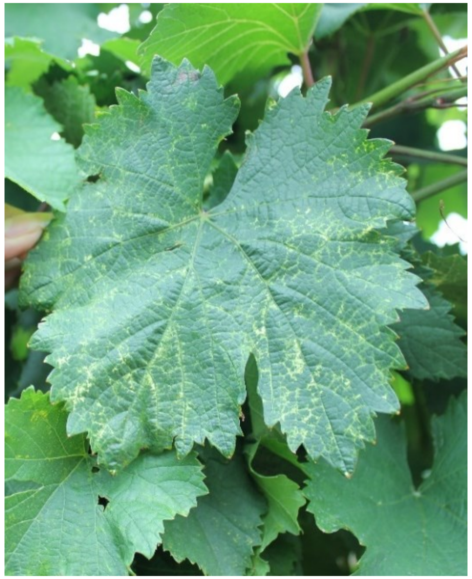

269 Rkatsiteli {GVT, GPGV, GRSPaV, GSyV-1, GFLV, GYSVd-1, HSVd}

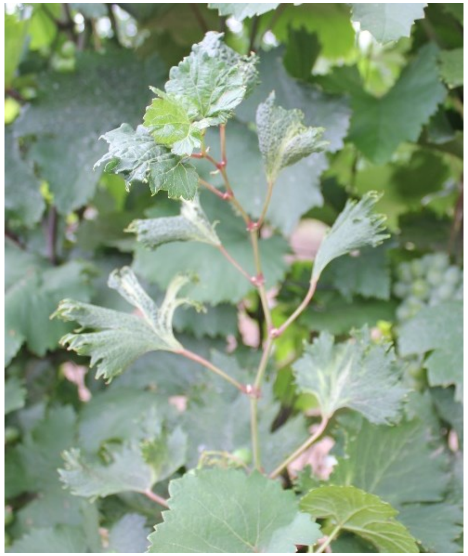
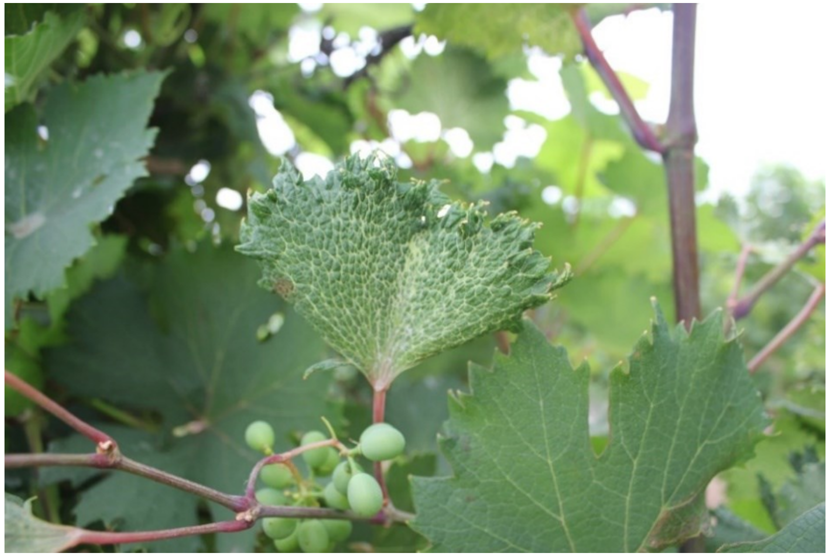
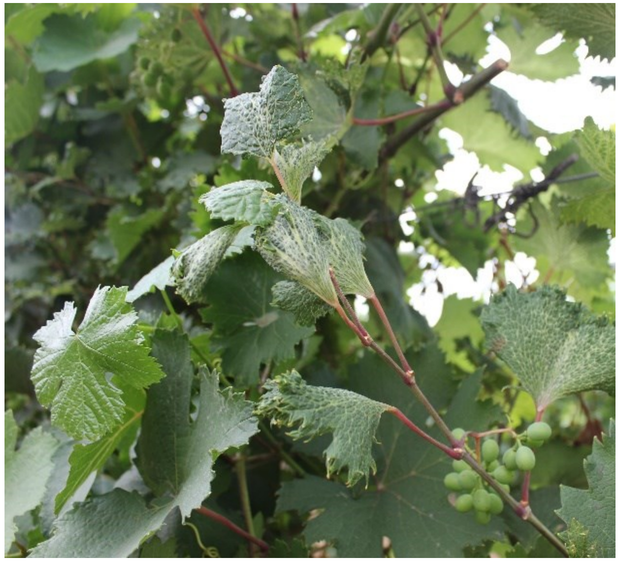

301 Zhupskij {GFkV, GVT, GPGV, GRVFV, GRSPaV, GVA, GSyV-1, GFLV, GYSVd-1, HSVd}

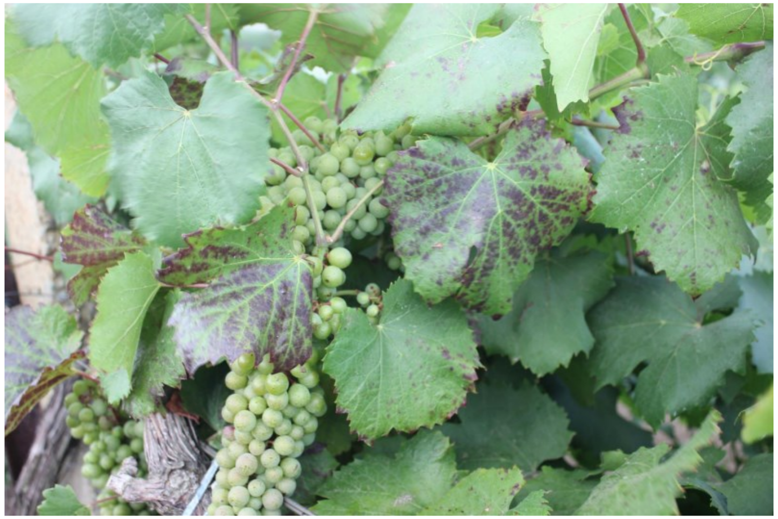

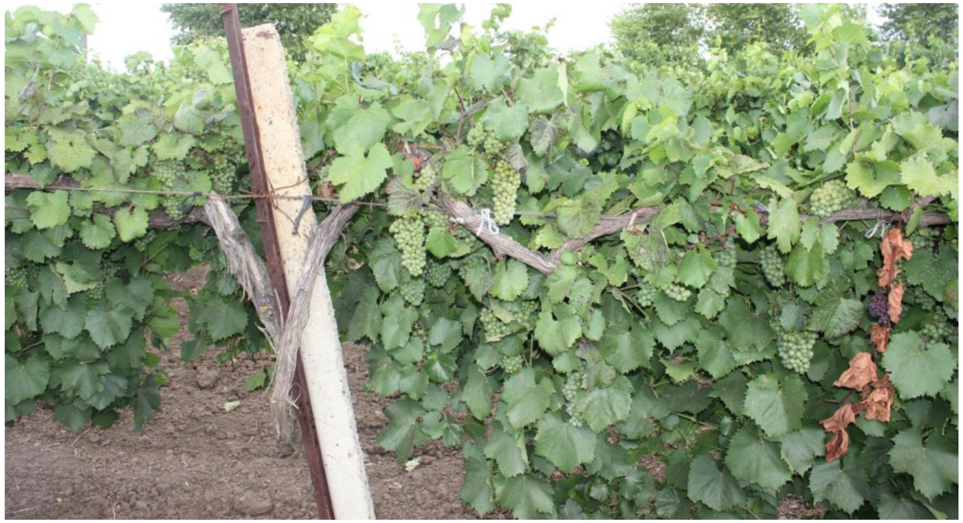

302 Cabernet Sauvignon {GPGV, GRSPaV, GSyV-1, GYSVd-1, GYSVd-2}

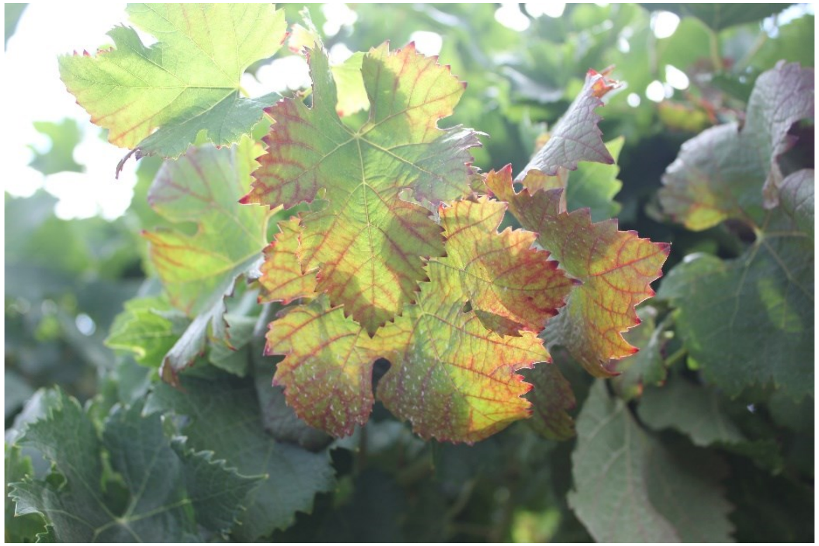

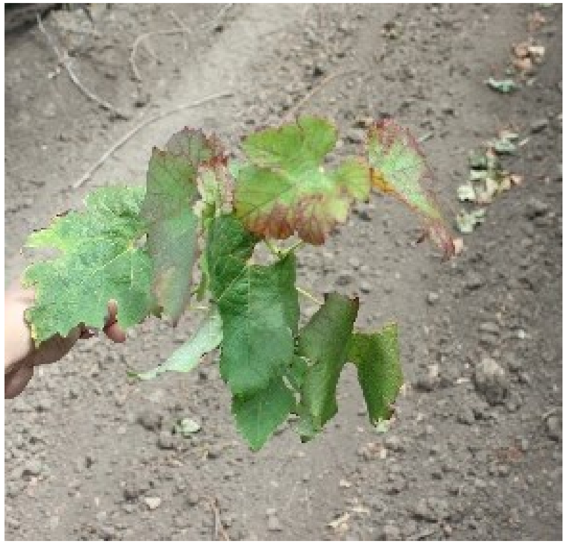

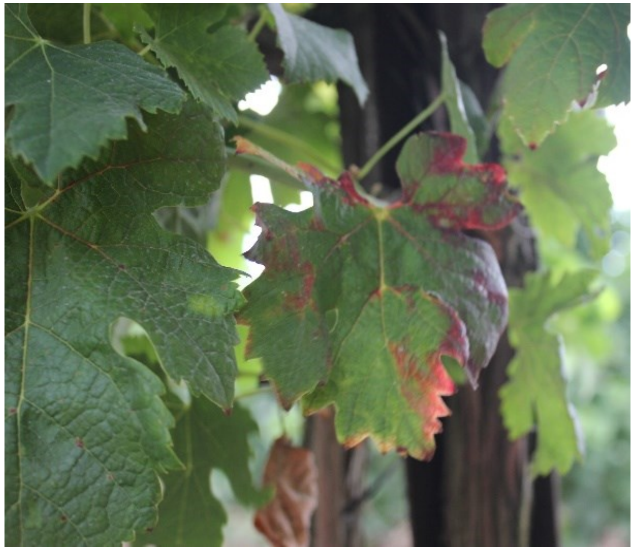

337 Cabernet Sauvignon {GFkV, GVT, GPGV, GRVFV, GRSPaV, GFLV, HSVd}

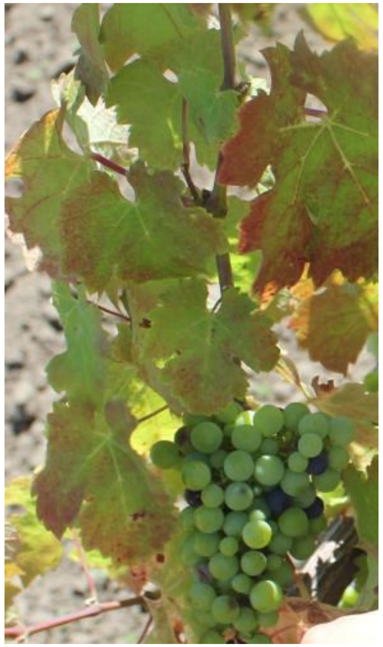

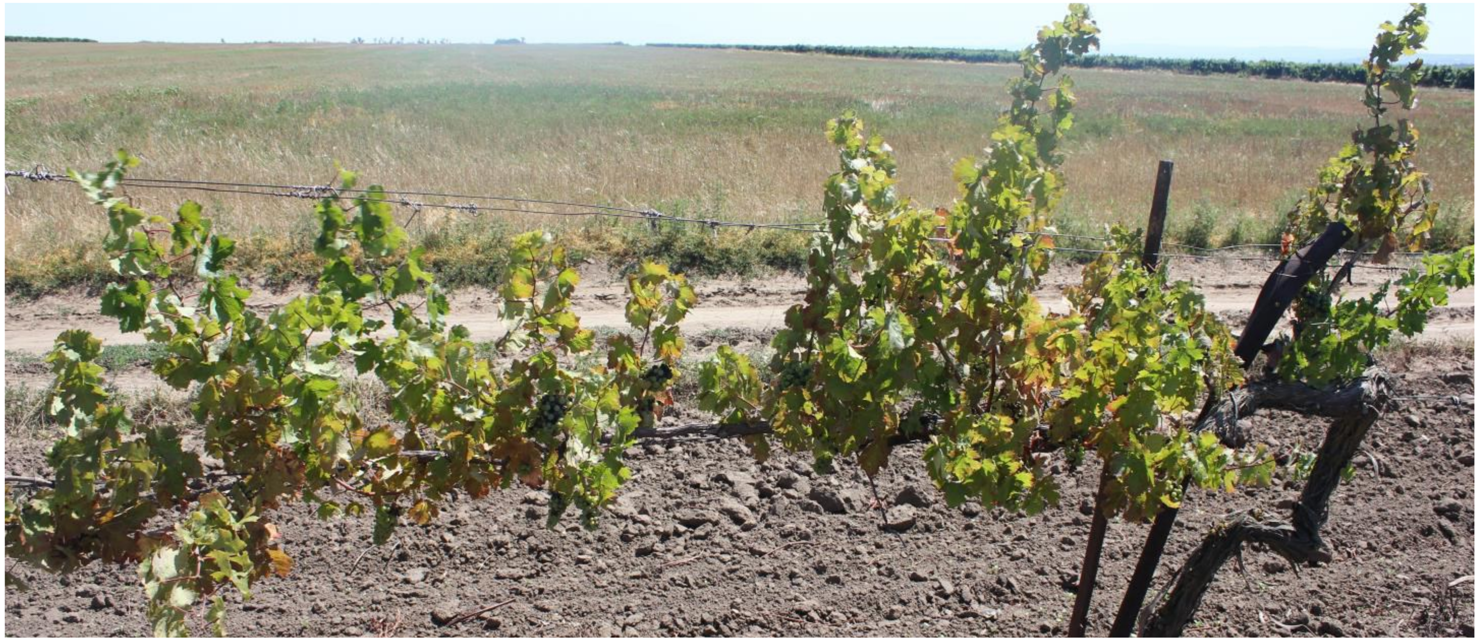

370 Cabernet Sauvignon {GFkV, GRVFV, GRSPaV, GSyV-1}

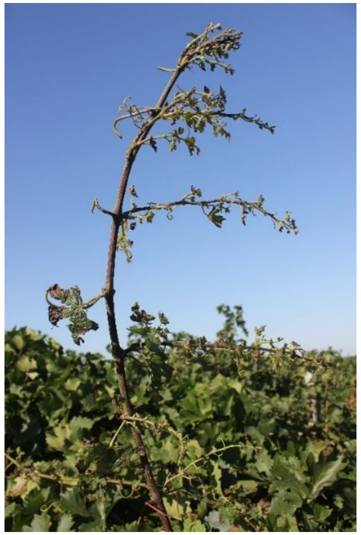

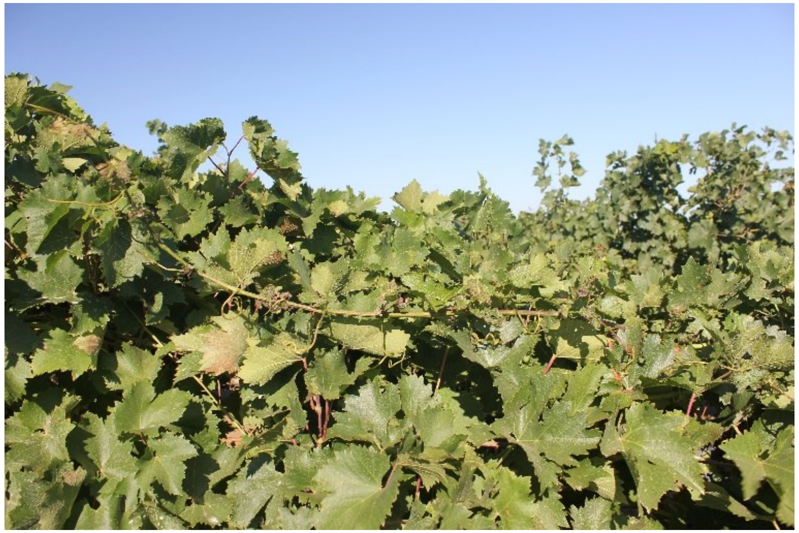

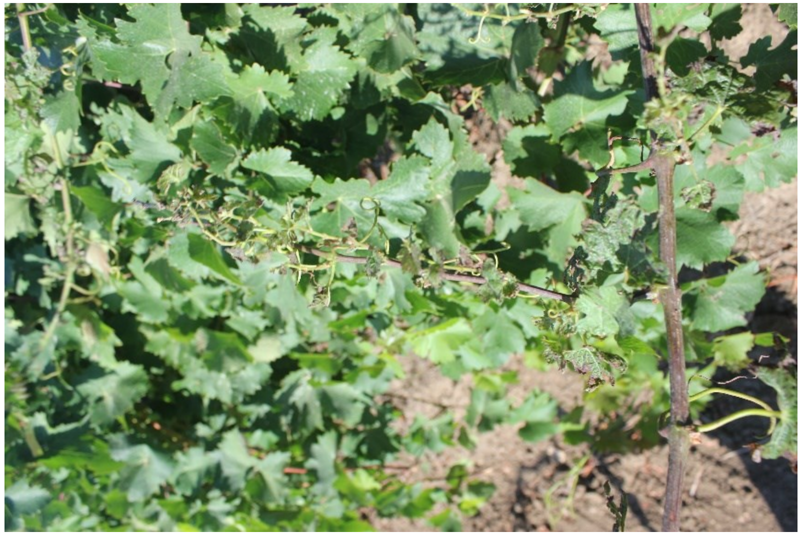

508 Augustine {GFkV, GPGV, GRSPaV, GSyV-1, GYSVd-1, HSVd}

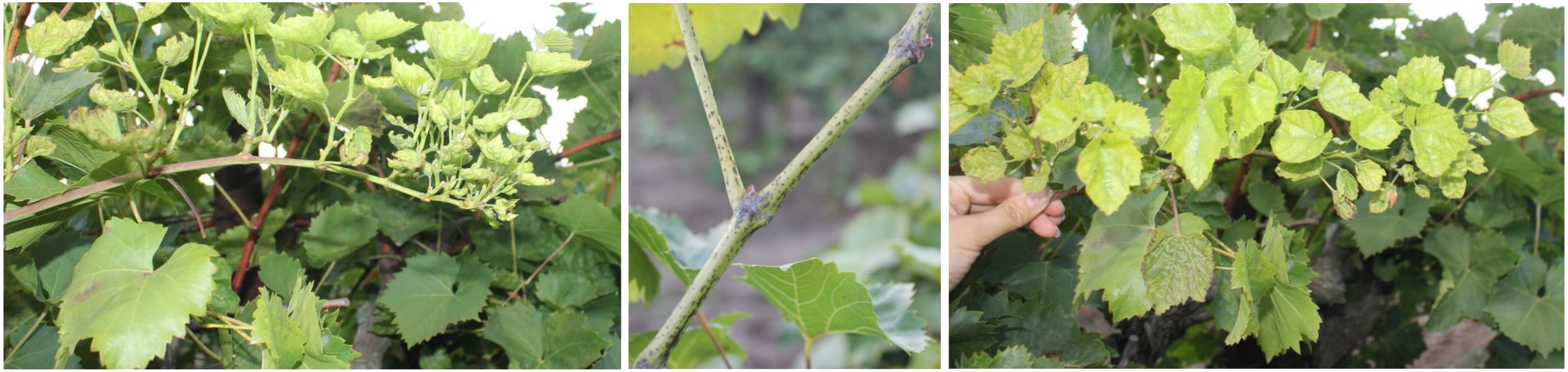

635 Frankovka {GFkV, GVT, GPGV, GRSPaV, GVA, GSyV-1, GYSVd-1, HSVd}

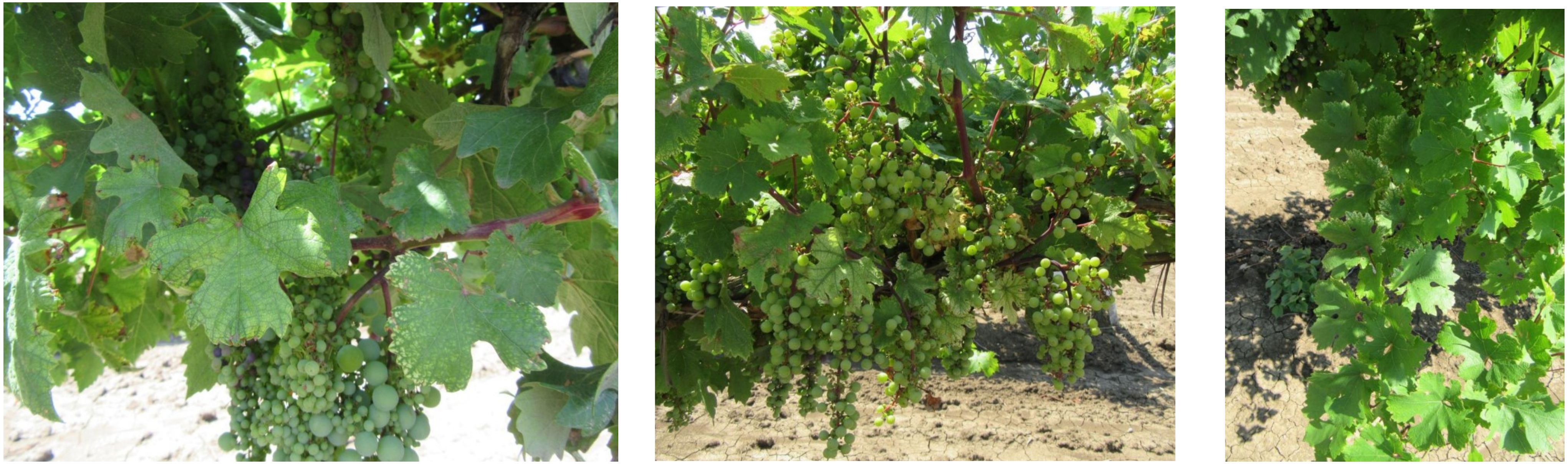

Library **Lib-06_S23**

384 Merlot {GFkV, GPGV, GRSPaV, GYSVd-1, HSVd}

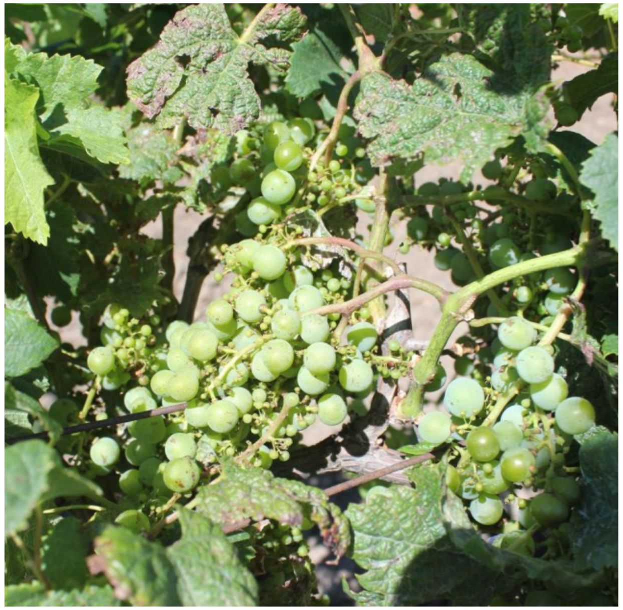
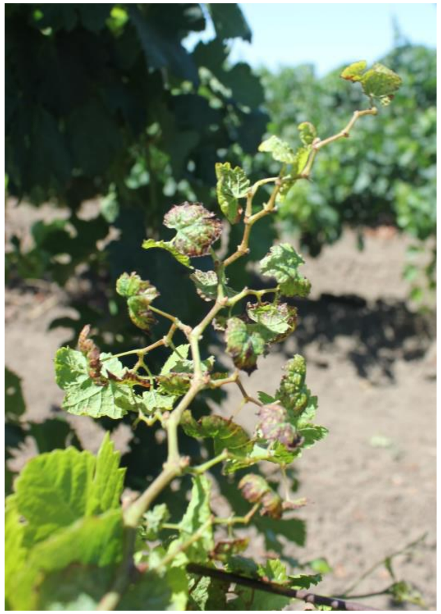
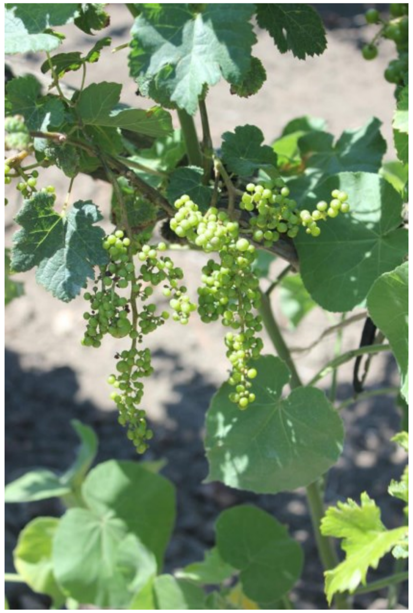
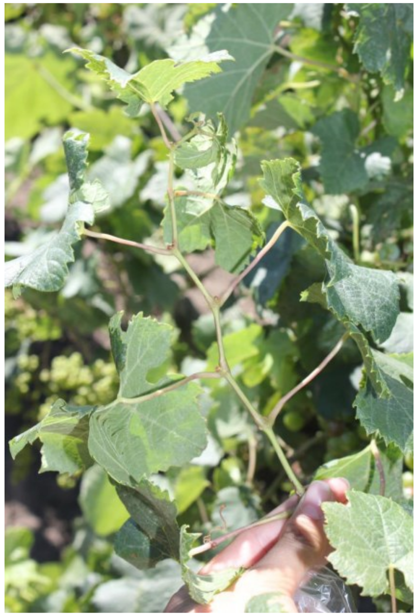

393 Isabella {GLRaV-1, GFkV, GPGV, GRSPaV, HSVd}

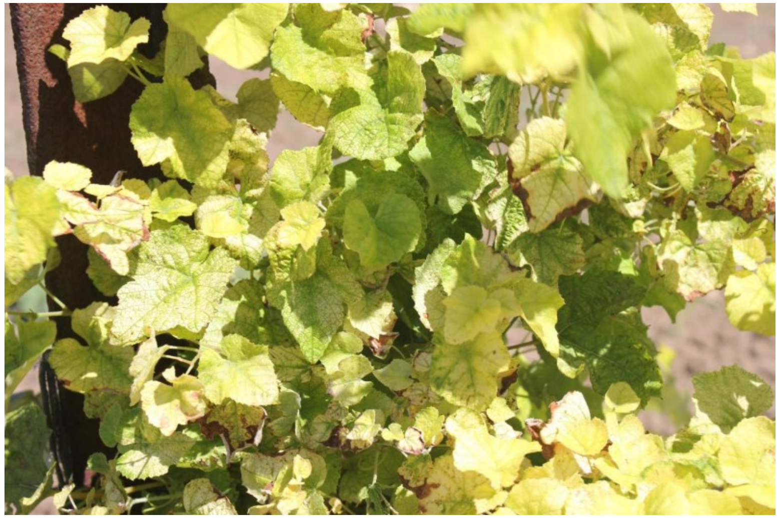
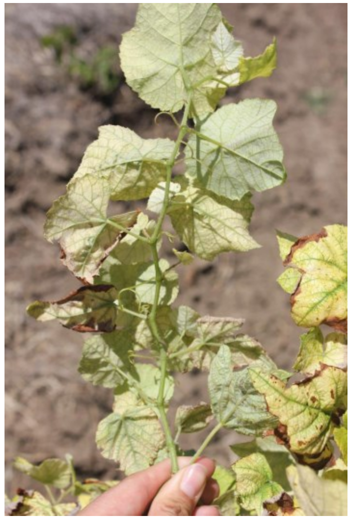
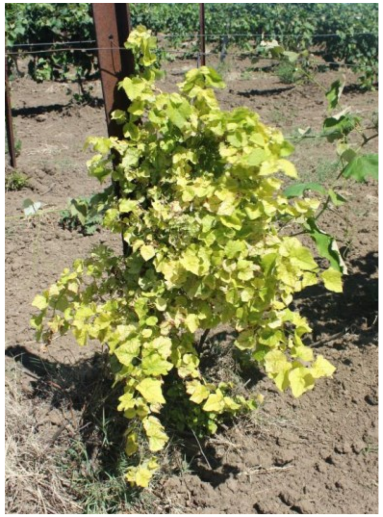

409 Syrah {GPGV, GRSPaV, GSyV-1, HSVd}

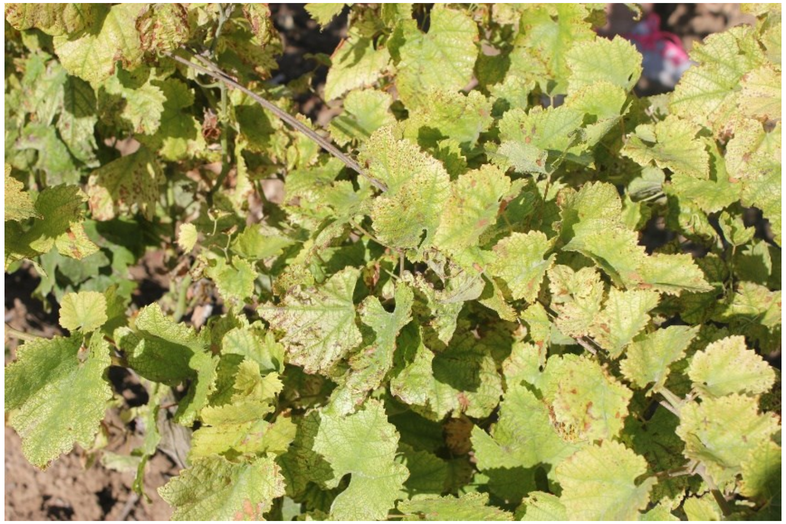
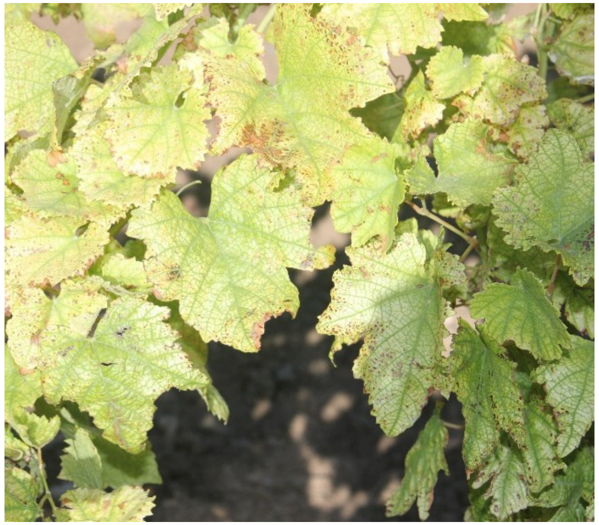
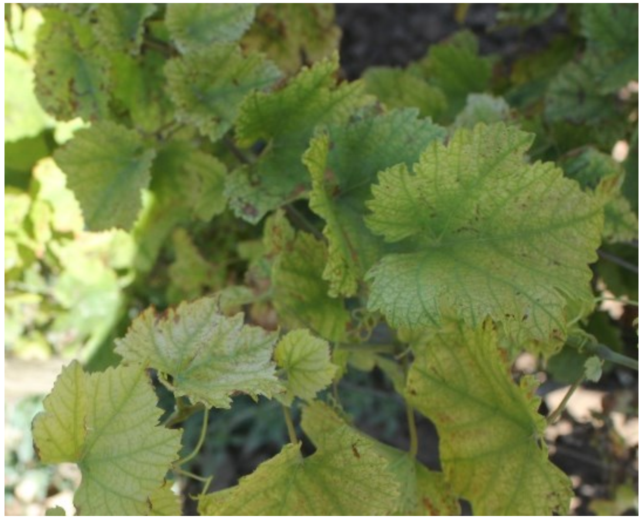

410 Syrah {GPGV, GRSPaV, GSyV-1, HSVd}

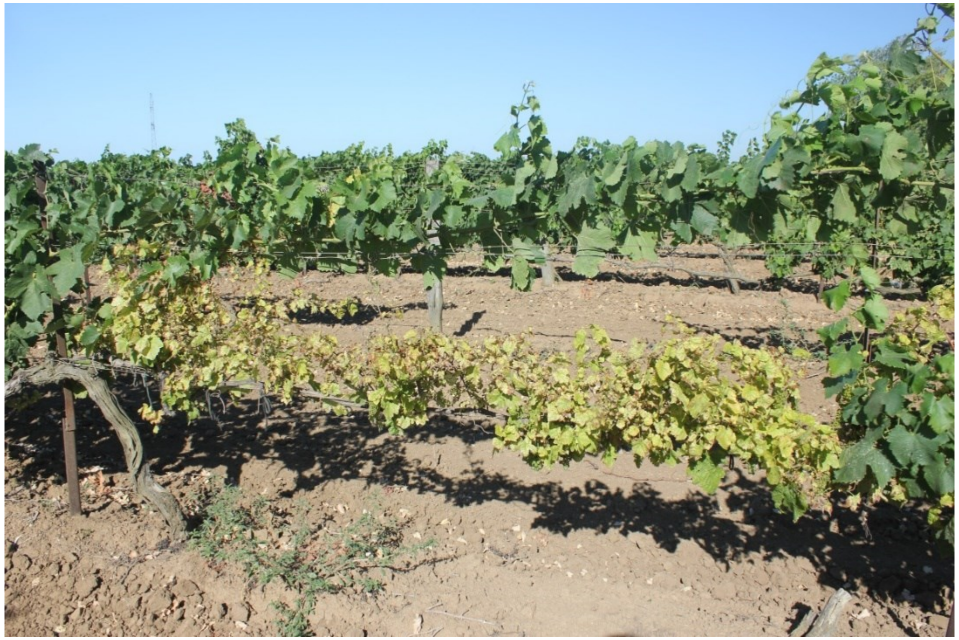
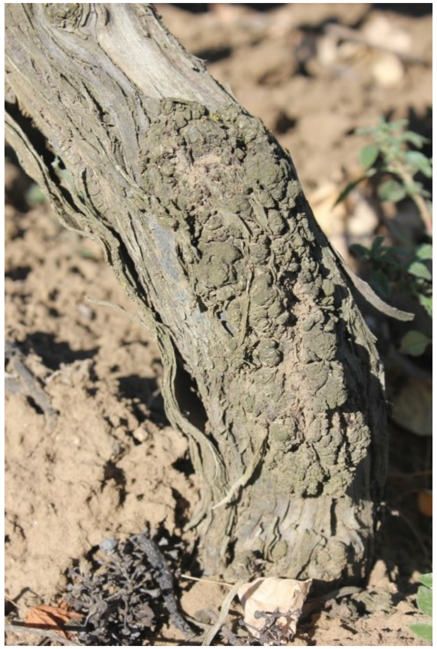
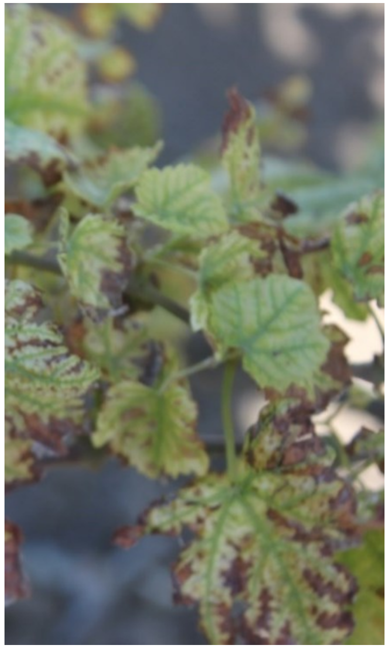

449 Chardonnay {GFkV, GPGV, GRVFV, GRSPaV, GVA, GSyV-1}

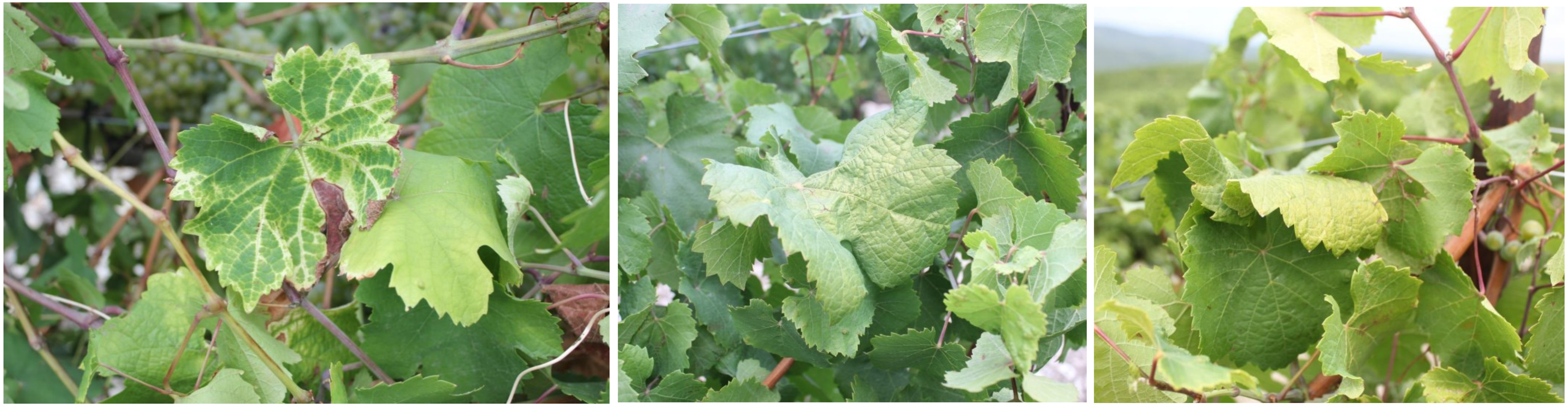

471 Aligoté {GLRaV-1, GVT, GVA, GSyV-1}

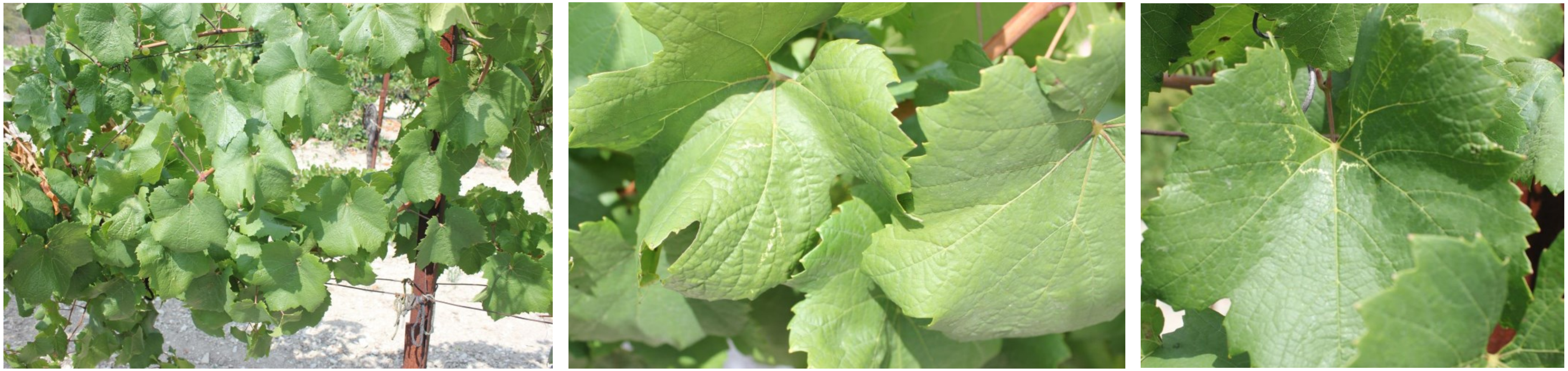

476 Riesling {GFkV, GRSPaV, GSyV-1, RBDV, GYSVd-1, HSVd}

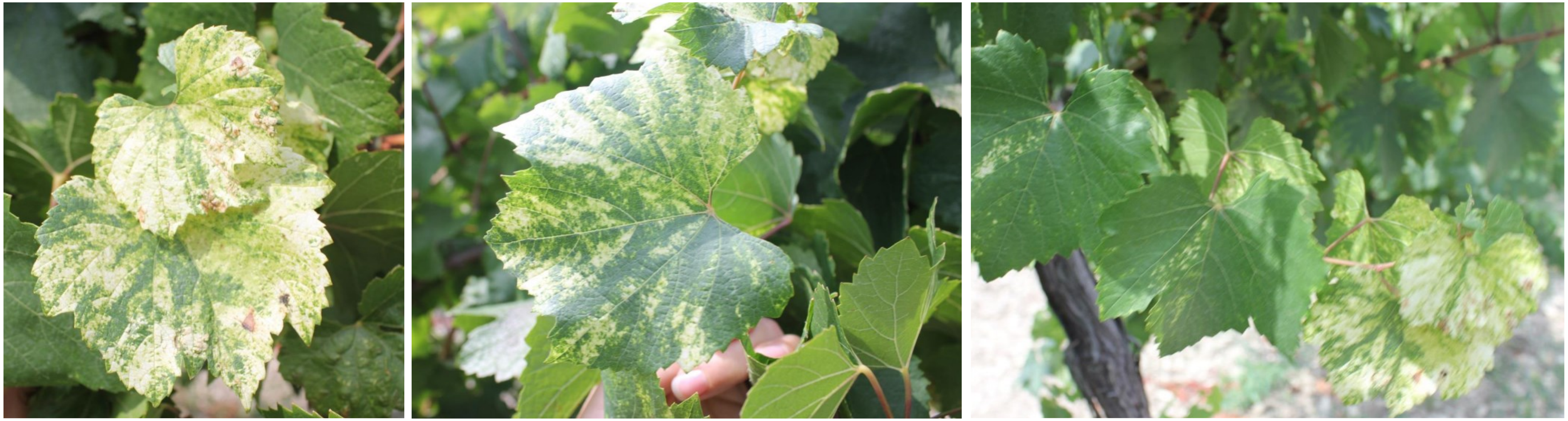

Supplement: Supplementary file 1 [file viruses-13-02432-s001.zip › Photographic material of symptoms.pdf]
