# Supplementary material for: High-Throughput Sequencing of Small RNAs for Diagnostics of Grapevine Viruses and Viroids in Russia
Source: Viruses. 2021 Dec 3;13(12):2432. doi: 10.3390/v13122432 (PMC8709451; doi:10.3390/v13122432)
Supplement: Supplementary file 1 [file viruses-13-02432-s001.zip › Supplementary Figures.pdf]

GLRaV-1 Coat protein gene

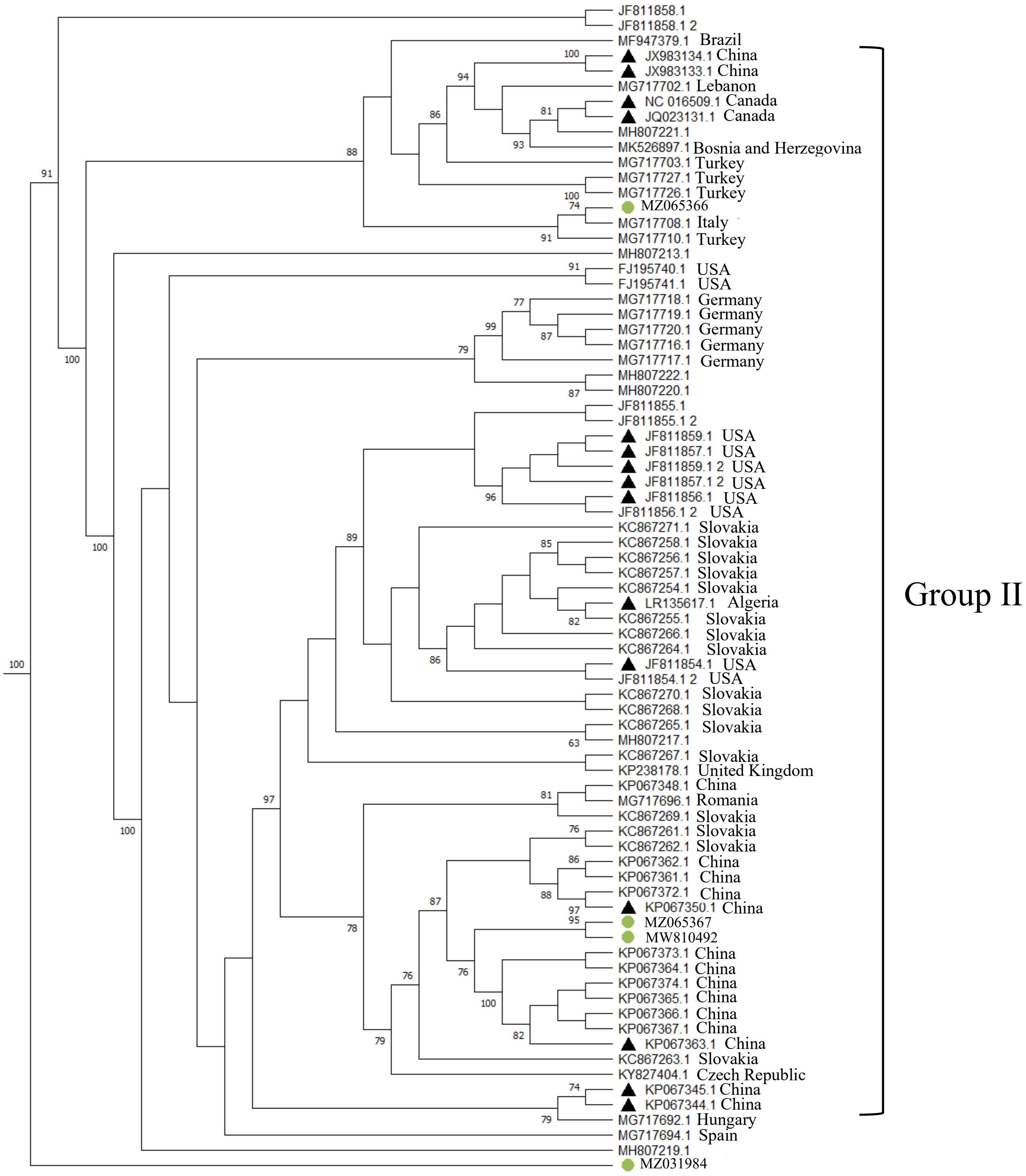

Figure S1. Phylogenetic analysis of global isolates of GLRaV-1 based on nucleotide sequences of the coat protein (cp) gene. Sequences previously reported by us and the corresponding sequences available from GenBank are identified by their isolate names, followed by their GenBank accession number and source. The isolates obtained in this study are indicated by 'green mark'; ▲- representative sequence for the group. The tree was constructed using the Maximum Likelihood method. The numbers at the nodes indicate bootstrap support (1,000 replicates). Values below 60% are not shown.

GLRaV-2 Coat protein gene

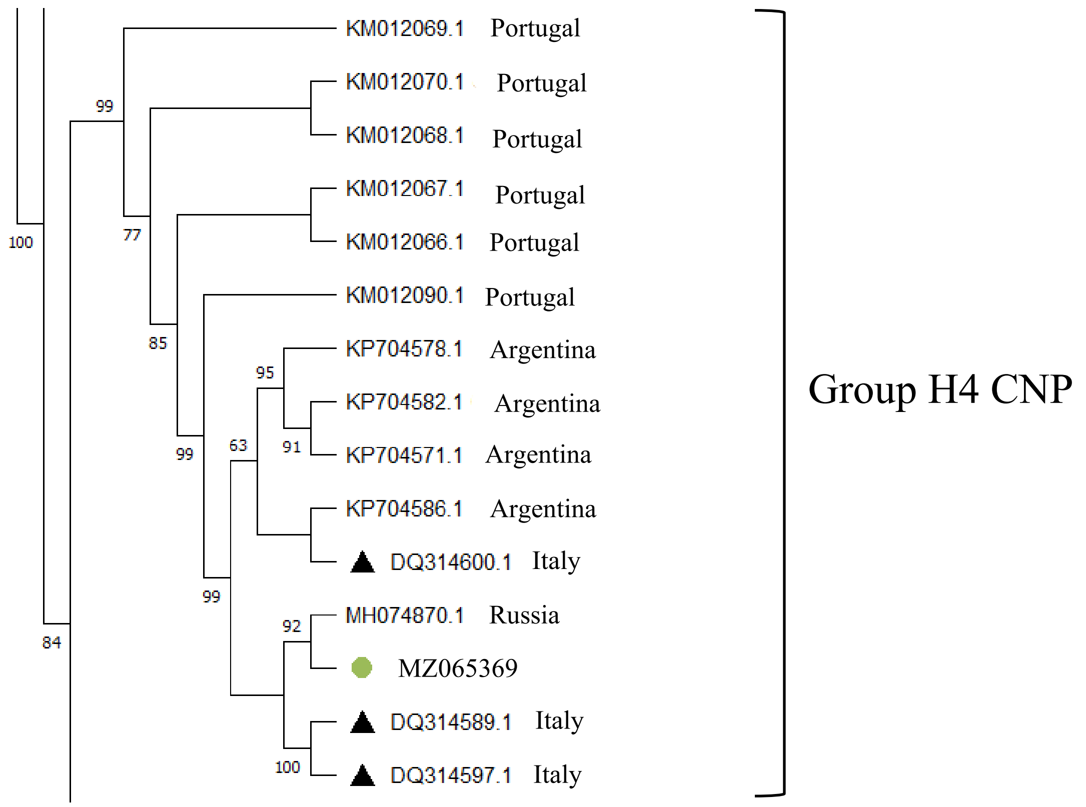

Figure S2. Phylogenetic analysis of global isolates of GLRaV-2 based on nucleotide sequences of the coat protein (cp) gene. Sequences previously reported by us and the corresponding sequences available from GenBank are identified by their isolate names, followed by their GenBank accession number and source. The isolates obtained in this study are indicated by 'green mark'; ▲- representative sequence for the group. The tree was constructed using the Maximum Likelihood method. The numbers at the nodes indicate bootstrap support (1,000 replicates). Values below 60% are not shown.

GLRaV-3 Coat protein gene

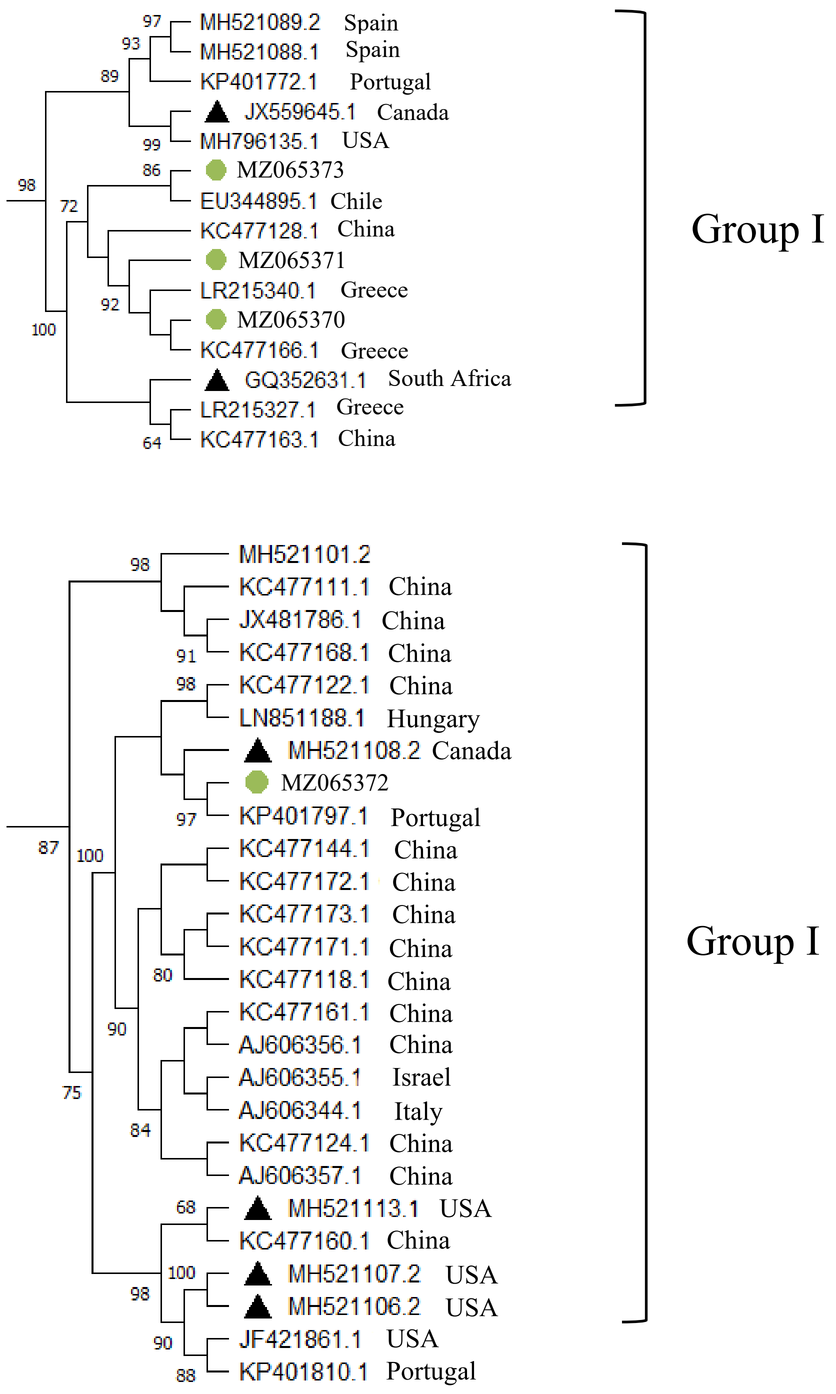

Figure S3. Phylogenetic analysis of global isolates of GLRaV-3 based on nucleotide sequences of the coat protein (cp) gene. Sequences previously reported by us and the corresponding sequences available from GenBank are identified by their isolate names, followed by their GenBank accession number and source. The isolates obtained in this study are indicated by 'green mark'; ▲- representative sequence for the group. The tree was constructed using the Maximum Likelihood method. The numbers at the nodes indicate bootstrap support (1,000 replicates). Values below 60% are not shown.

GLRaV-4 HSP70 like protein

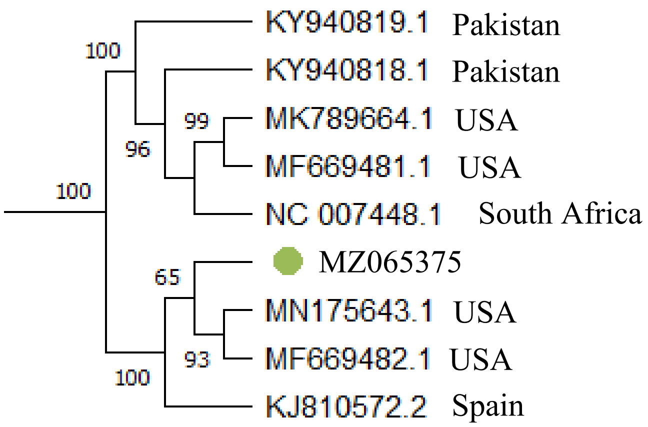

Figure S4. Phylogenetic analysis of global isolates of GLRaV-4 based on total nucleotide sequences from NCBI. Sequences previously reported by us and the corresponding sequences available from GenBank are identified by their isolate names, followed by their GenBank accession number and source. The isolates obtained in this study are indicated by 'green mark'. The tree was constructed using the Maximum Likelihood method. The numbers at the nodes indicate bootstrap support (1,000 replicates). Values below 60% are not shown.

GFLV Polyprotein gene

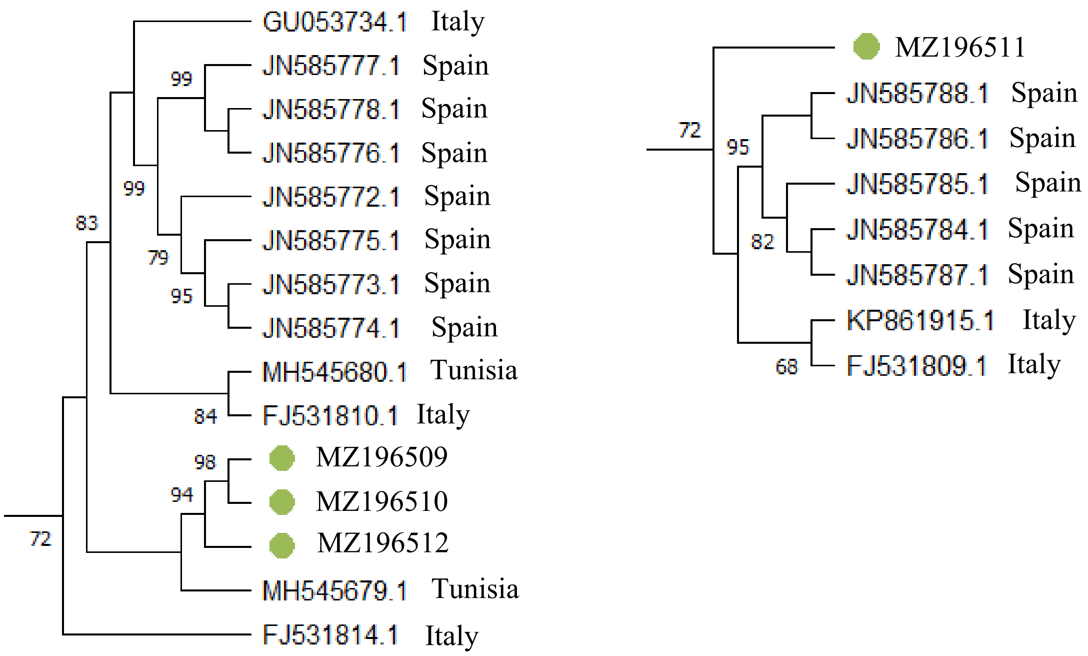

Figure S5. Phylogenetic analysis of global isolates of GFLV based on nucleotide sequences of the polyprotein gene. Sequences previously reported by us and the corresponding sequences available from GenBank are identified by their isolate names, followed by their GenBank accession number and source. The isolates obtained in this study are indicated by 'green mark'. The tree was constructed using the Maximum Likelihood method. The numbers at the nodes indicate bootstrap support (1,000 replicates). Values below 60% are not shown.

GVA   Coat protein gene

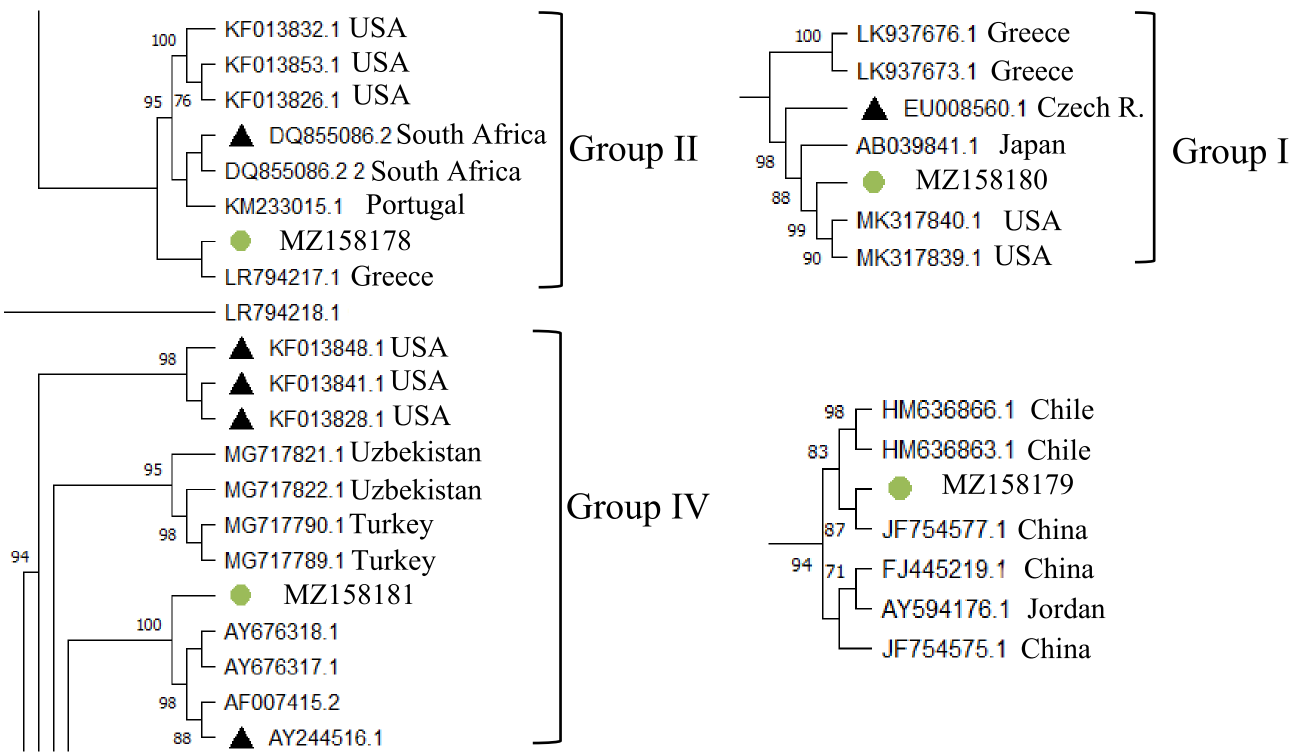

Figure S6. Phylogenetic analysis of global isolates of GVA based on nucleotide sequences of the coat protein (cp) gene. Sequences previously reported by us and the corresponding sequences available from GenBank are identified by their isolate names, followed by their GenBank accession number and source. The isolates obtained in this study are indicated by 'green mark'; ▲- representative sequence for the group. The tree was constructed using the Maximum Likelihood method. The numbers at the nodes indicate bootstrap support (1,000 replicates). Values below 60% are not shown.

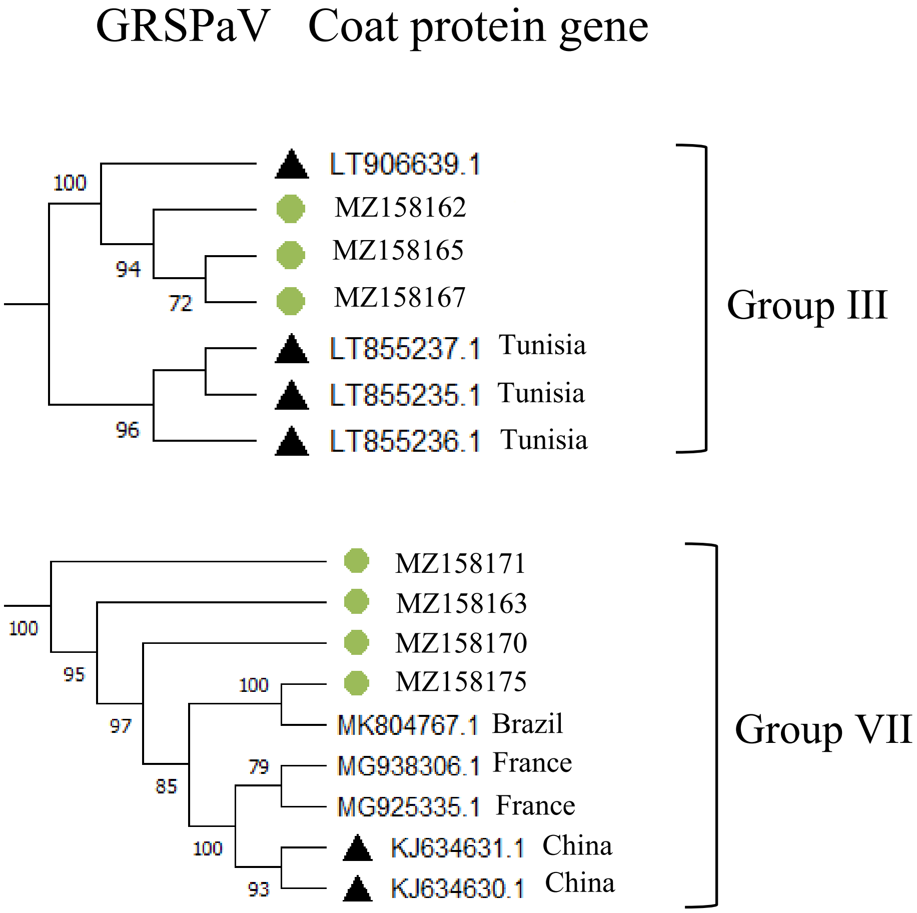

Figure S7. Phylogenetic analysis of global isolates of GRSPaV based on nucleotide sequences of the coat protein (cp) gene. Sequences previously reported by us and the corresponding sequences available from GenBank are identified by their isolate names, followed by their GenBank accession number and source. The isolates obtained in this study are indicated by 'green mark'; ▲- representative sequence for the group. The tree was constructed using the Maximum Likelihood method. The numbers at the nodes indicate bootstrap support (1,000 replicates). Values below 60% are not shown.

GVT Coat protein gene

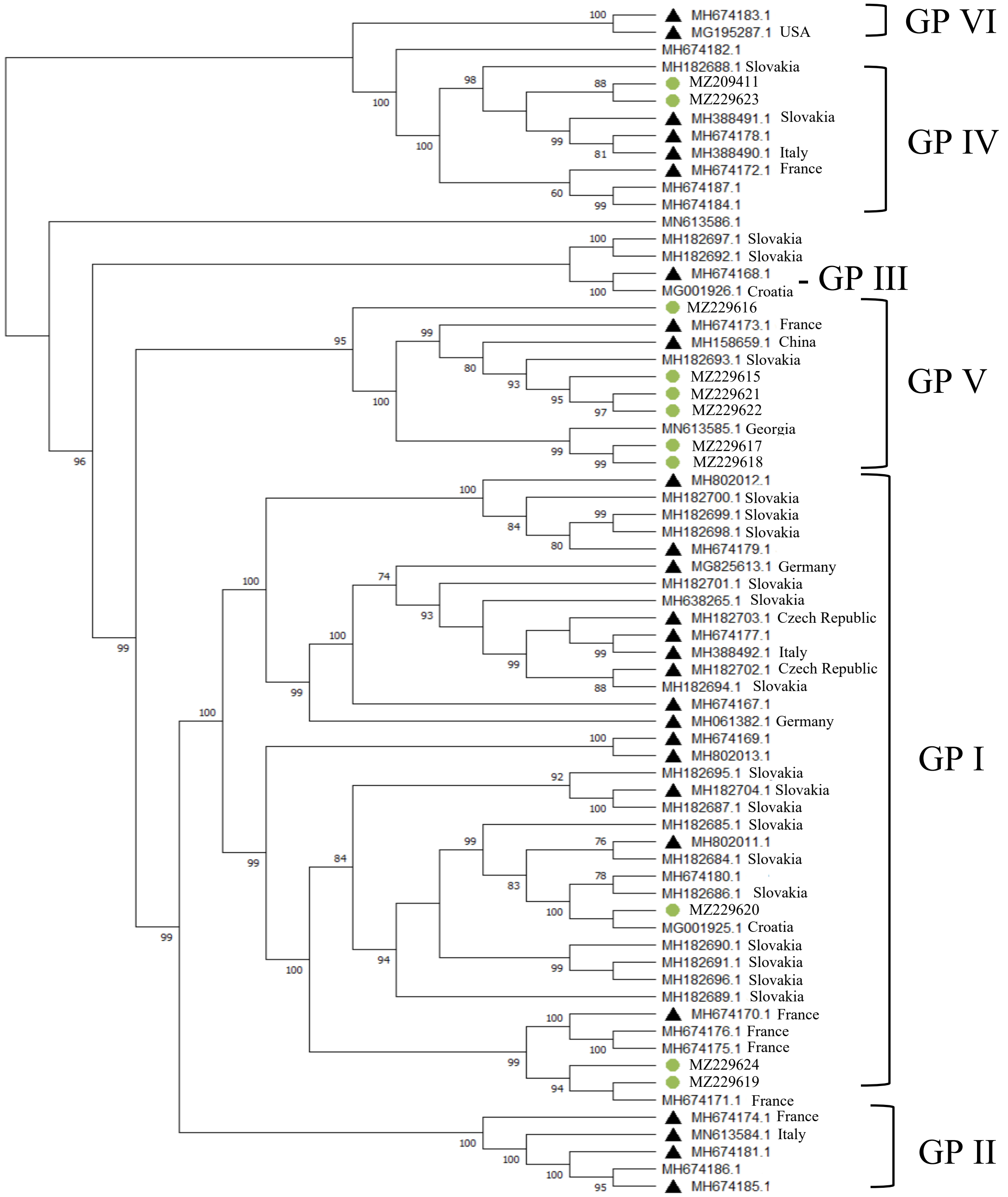

Figure S8. Phylogenetic analysis of global isolates of GVT based on total nucleotide from NCBI. Sequences previously reported by us and the corresponding sequences available from GenBank are identified by their isolate names, followed by their GenBank accession number and source. The isolates obtained in this study are indicated by 'green mark'; ▲- representative sequence for the group. The tree was constructed using the Maximum Likelihood method. The numbers at the nodes indicate bootstrap support (1,000 replicates). Values below 60% are not shown.

GPGV Coat protein gene

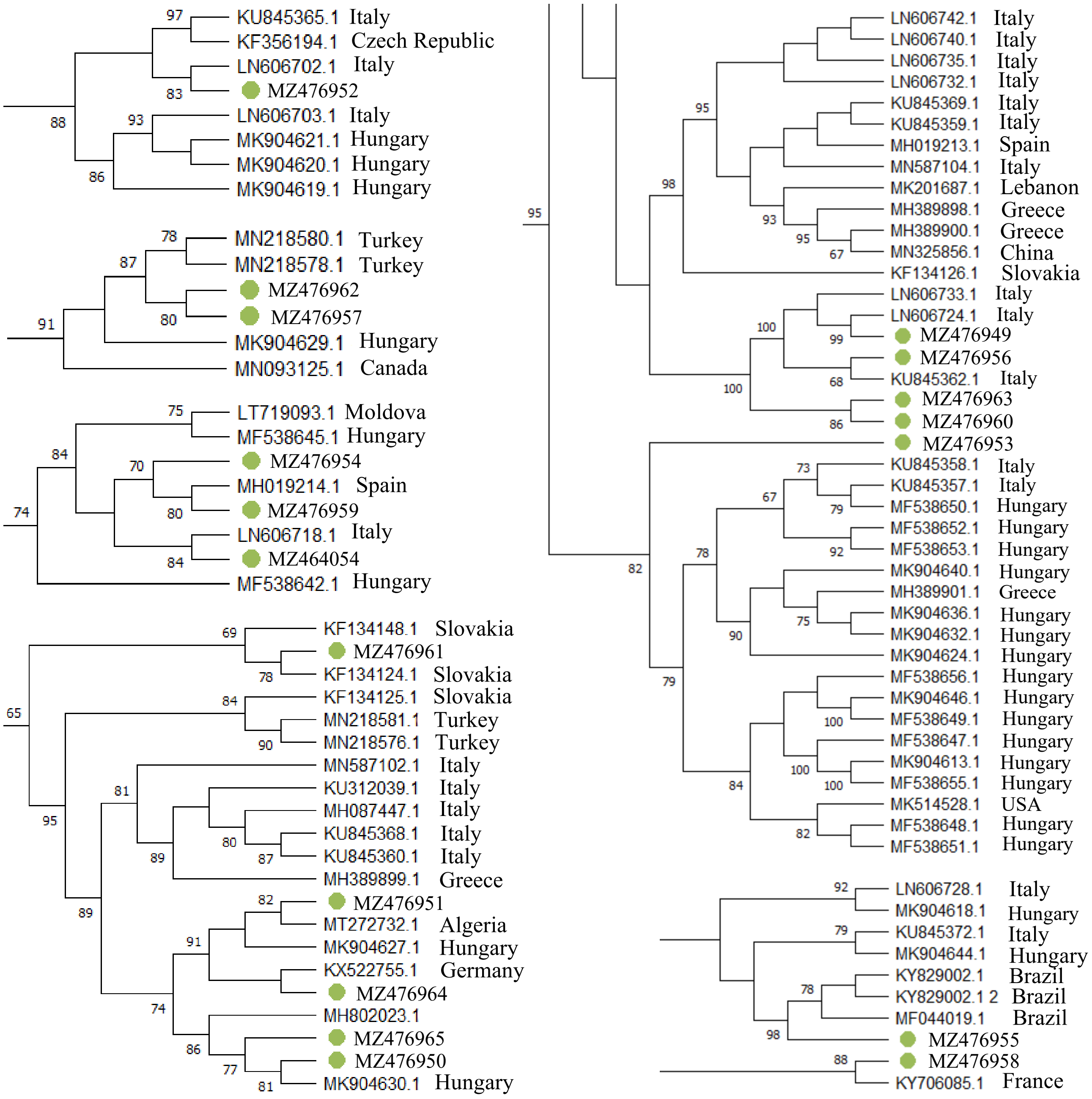

Figure S9. Phylogenetic analysis of global isolates of GPGV based on nucleotide sequences of the coat protein (cp) gene. Sequences previously reported by us and the corresponding sequences available from GenBank are identified by their isolate names, followed by their GenBank accession number and source. The isolates obtained in this study are indicated by 'green mark'. The tree was constructed using the Maximum Likelihood method. The numbers at the nodes indicate bootstrap support (1,000 replicates). Values below 60% are not shown.

GFkV Coat protein gene

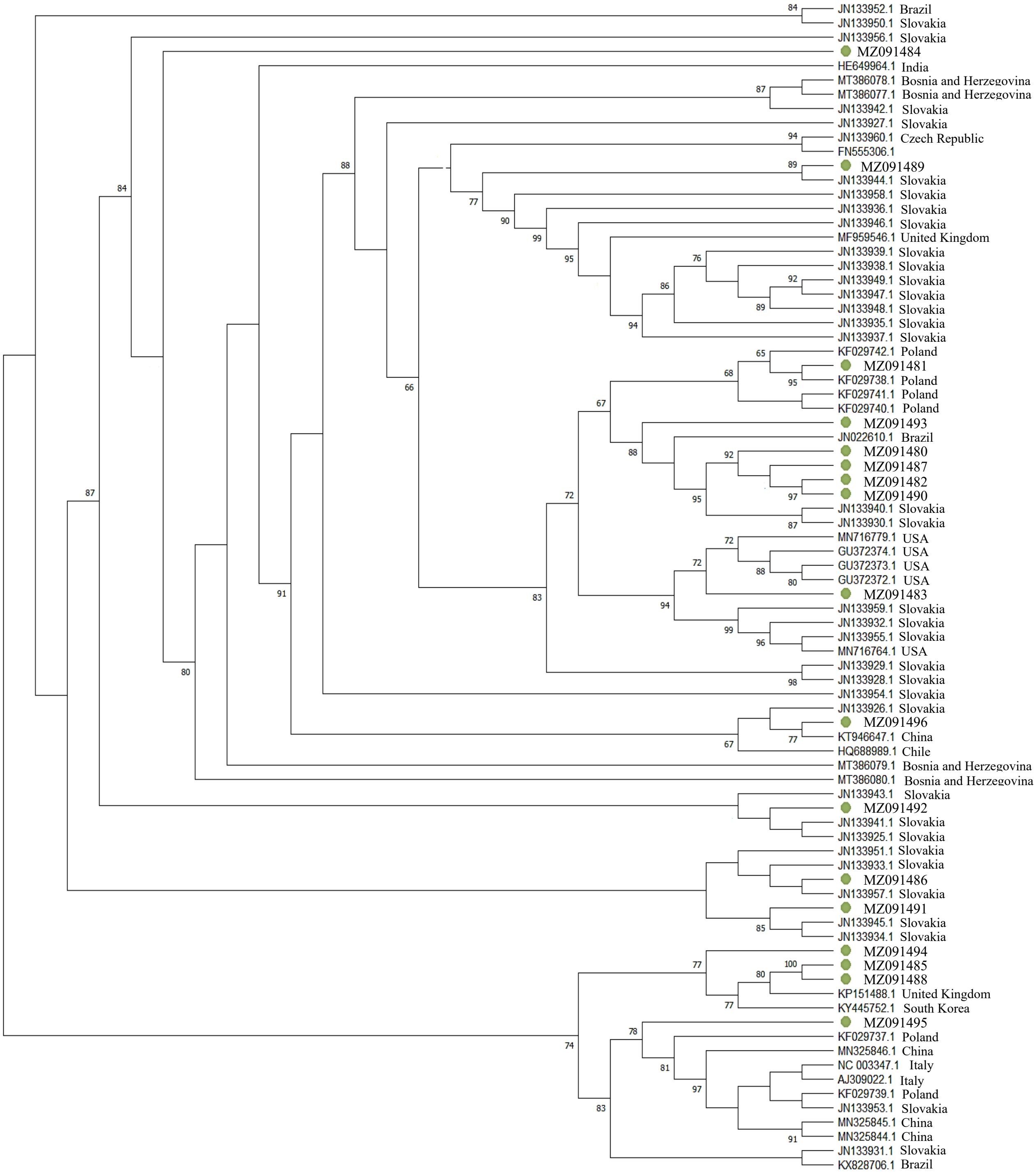

Figure S10. Phylogenetic analysis of global isolates of GFkV based on nucleotide sequences of the coat protein (cp) gene. Sequences previously reported by us and the corresponding sequences available from GenBank are identified by their isolate names, followed by their GenBank accession number and source. The isolates obtained in this study are indicated by 'green mark'. The tree was constructed using the Maximum Likelihood method. The numbers at the nodes indicate bootstrap support (1,000 replicates). Values below 60% are not shown.

GRVFV Polyprotein gene

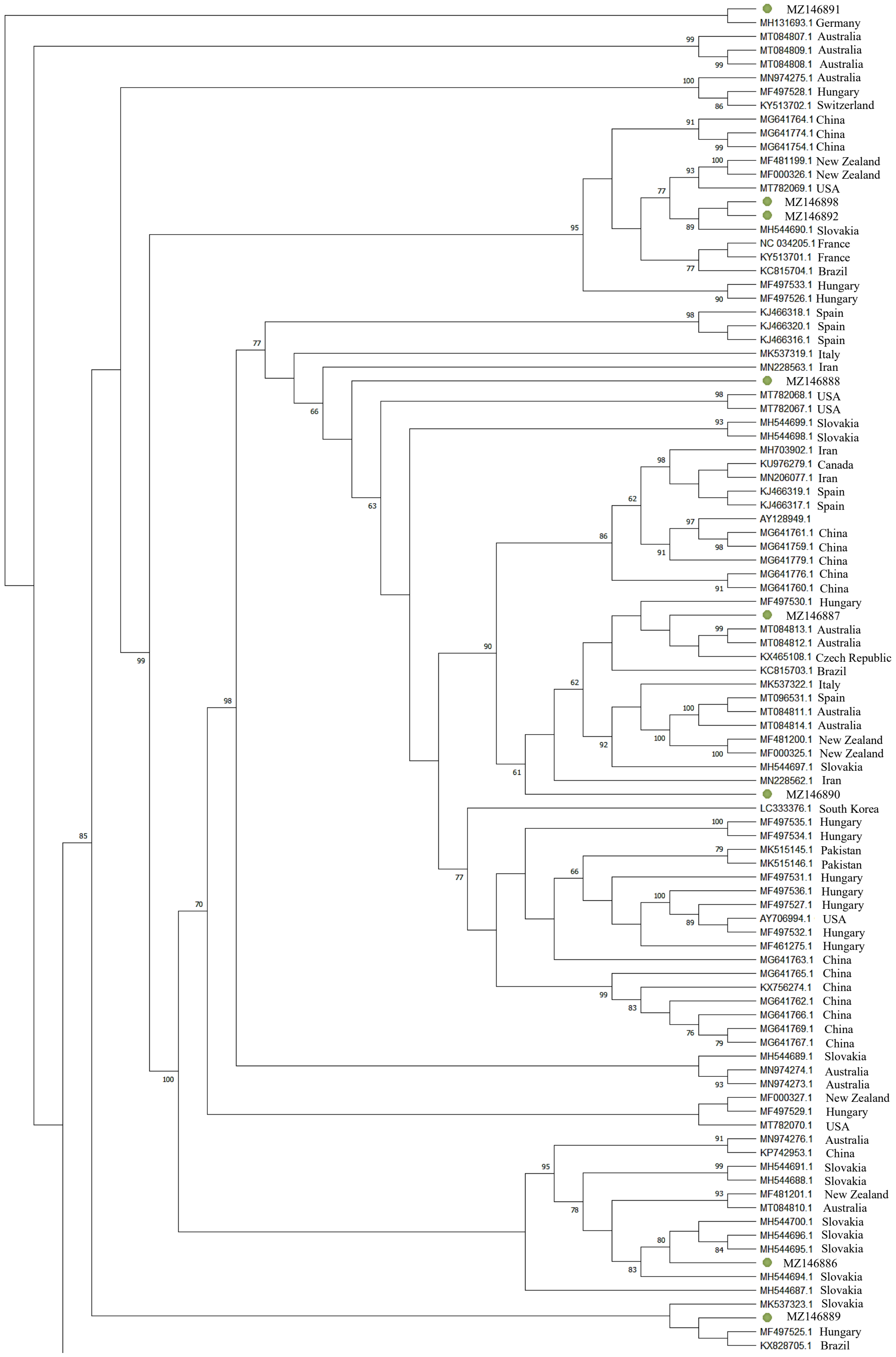

Figure S11. Phylogenetic analysis of global isolates of GRVFV based on total nucleotide sequences from NCBI. Sequences previously reported by us and the corresponding sequences available from GenBank are identified by their isolate names, followed by their GenBank accession number and source. The isolates obtained in this study are indicated by 'green mark'. The tree was constructed using the Maximum Likelihood method. The numbers at the nodes indicate bootstrap support (1,000 replicates). Values below 60% are not shown.

GSyV-1 Coat protein gene

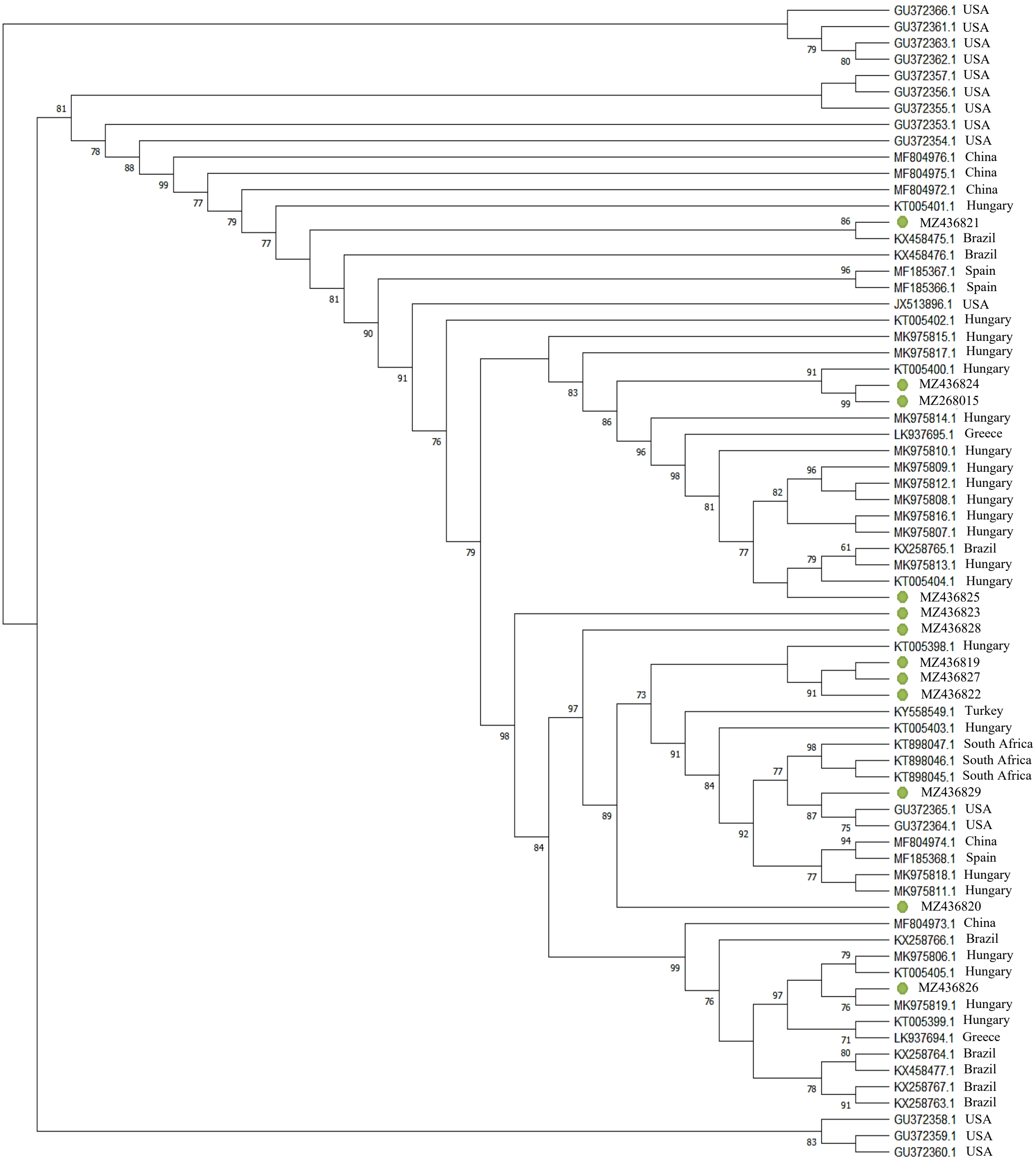

Figure S12. Phylogenetic analysis of global isolates of GSyV-1 based on nucleotide sequences of the coat protein (cp) gene. Sequences previously reported by us and the corresponding sequences available from GenBank are identified by their isolate names, followed by their GenBank accession number and source. The isolates obtained in this study are indicated by ‘green mark’. The tree was constructed using the Maximum Likelihood method. The numbers at the nodes indicate bootstrap support (1,000 replicates). Values below 60% are not shown.

RBDV – RNA2

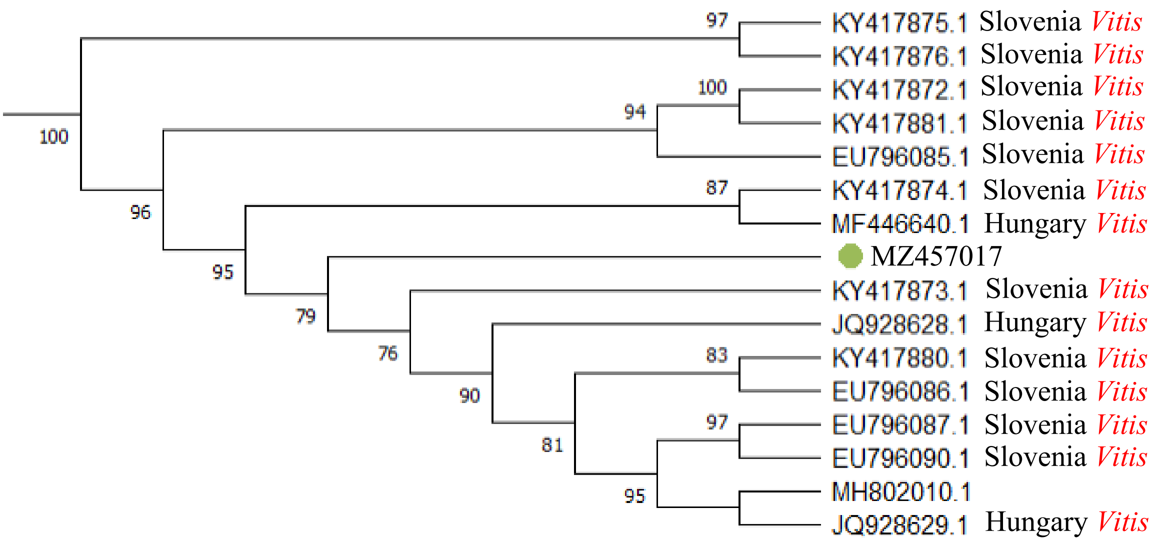

RBDV – RNA1

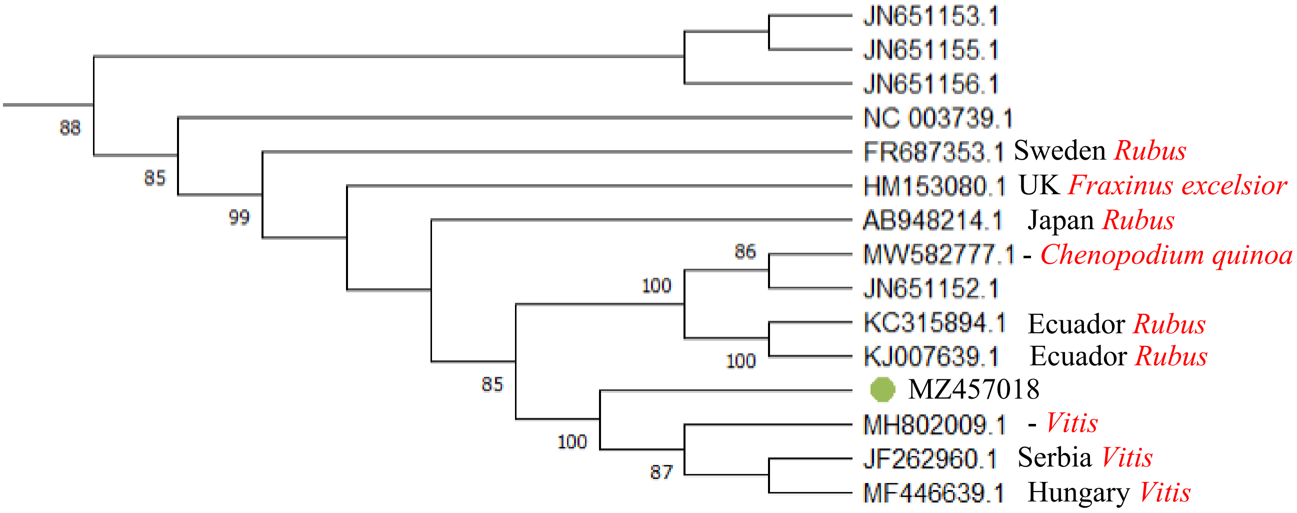

Figure S13. Phylogenetic analysis of global isolates of RBDV based on based on total nucleotide from NCBI. Sequences previously reported by us and the corresponding sequences available from GenBank are identified by their isolate names, followed by their GenBank accession number and source. The isolates obtained in this study are indicated by 'green mark'. The tree was constructed using the Maximum Likelihood method. The numbers at the nodes indicate bootstrap support (1,000 replicates). Values below 60% are not shown.

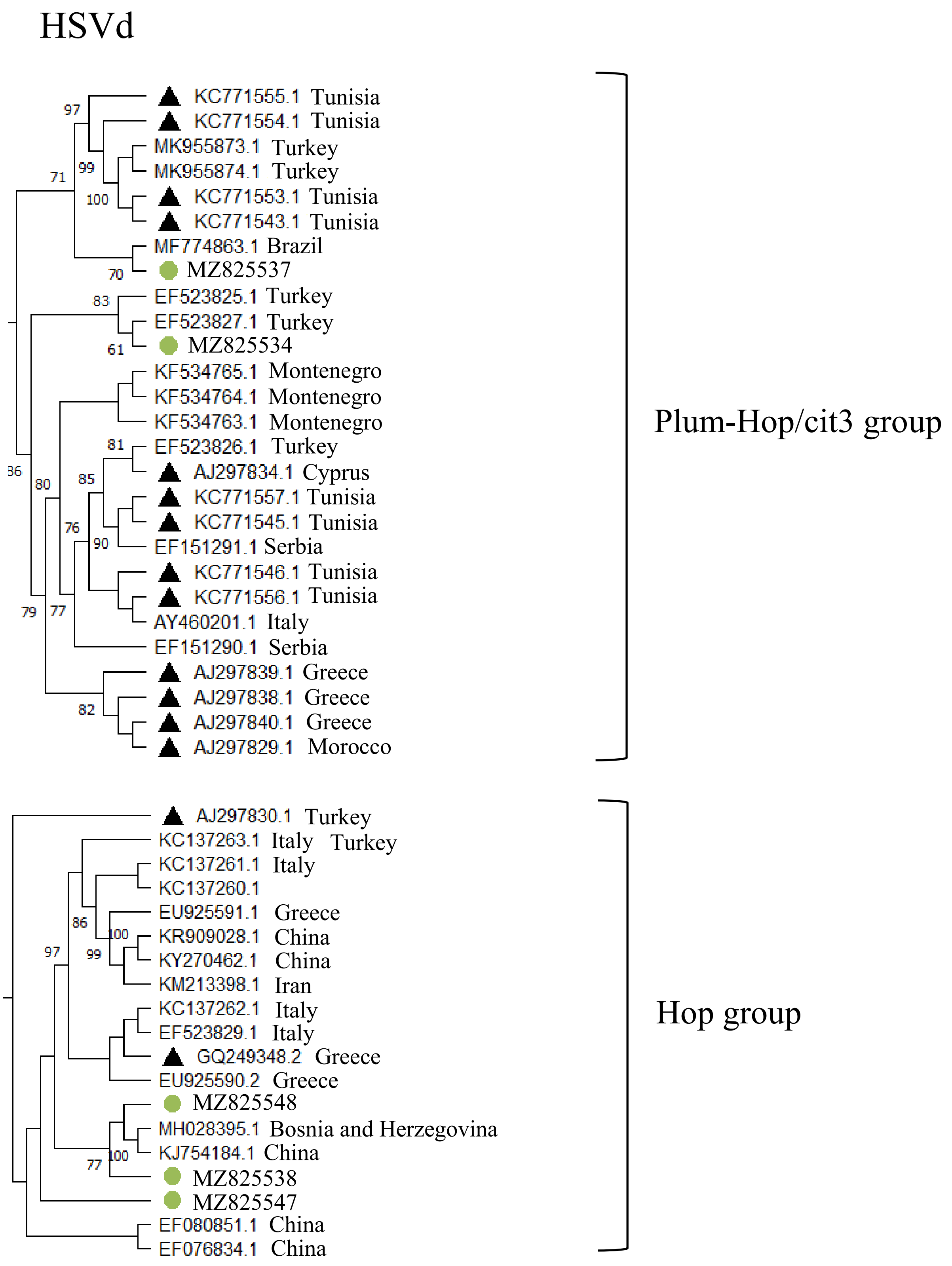

Figure S14. Phylogenetic analysis of global isolates of HSVd based on based on total nucleotide from NCBI. Sequences previously reported by us and the corresponding sequences available from GenBank are identified by their isolate names, followed by their GenBank accession number and source. The isolates obtained in this study are indicated by ‘green mark’, ▲- representative sequence for the group. The tree was constructed using the Maximum Likelihood method. The numbers at the nodes indicate bootstrap support (1,000 replicates). Values below 60% are not shown.

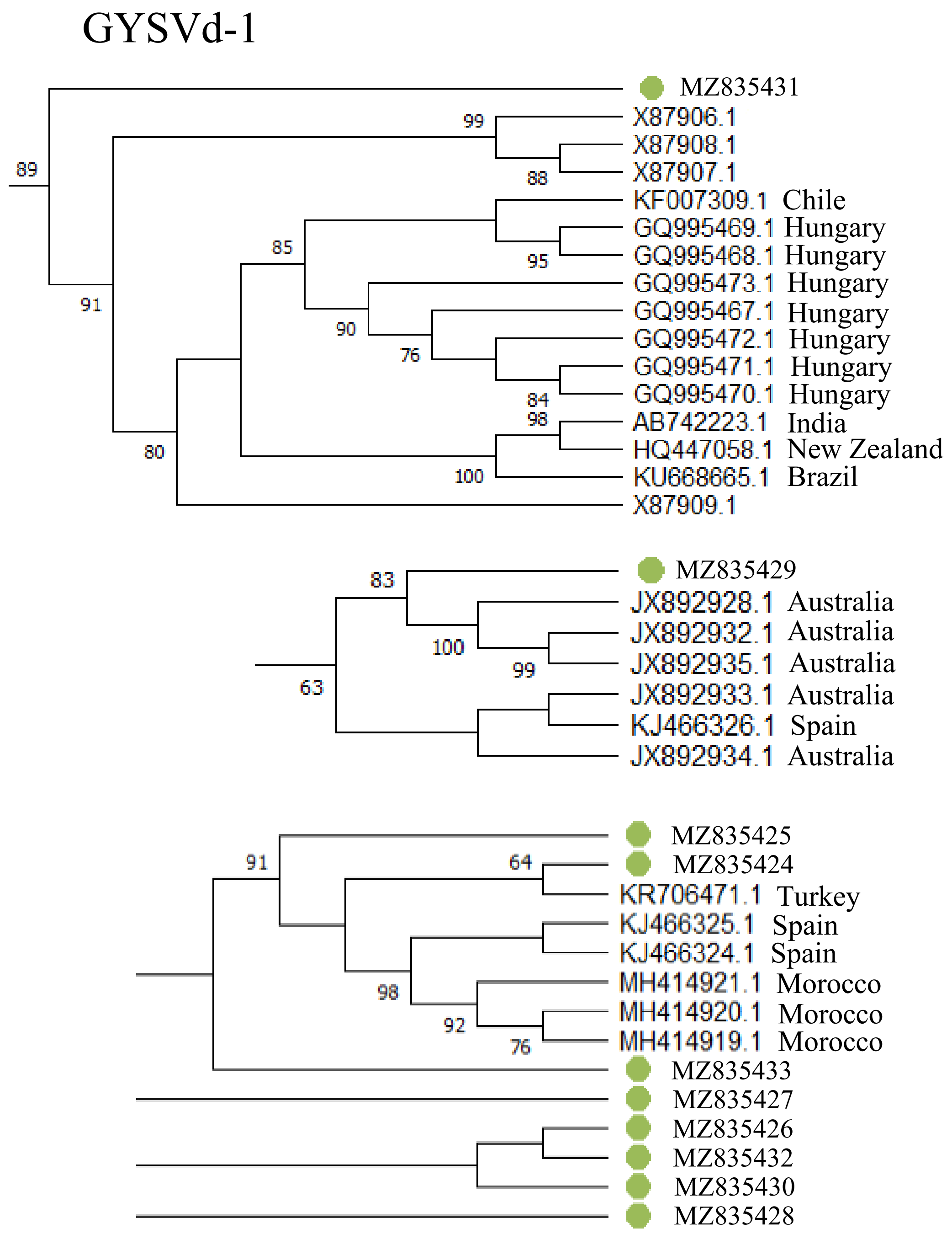

Figure S15. Phylogenetic analysis of global isolates of GYSVd-1 based on based on total nucleotide from NCBI. Sequences previously reported by us and the corresponding sequences available from GenBank are identified by their isolate names, followed by their GenBank accession number and source. The isolates obtained in this study are indicated by 'green mark'. The tree was constructed using the Maximum Likelihood method. The numbers at the nodes indicate bootstrap support (1,000 replicates). Values below 60% are not shown.

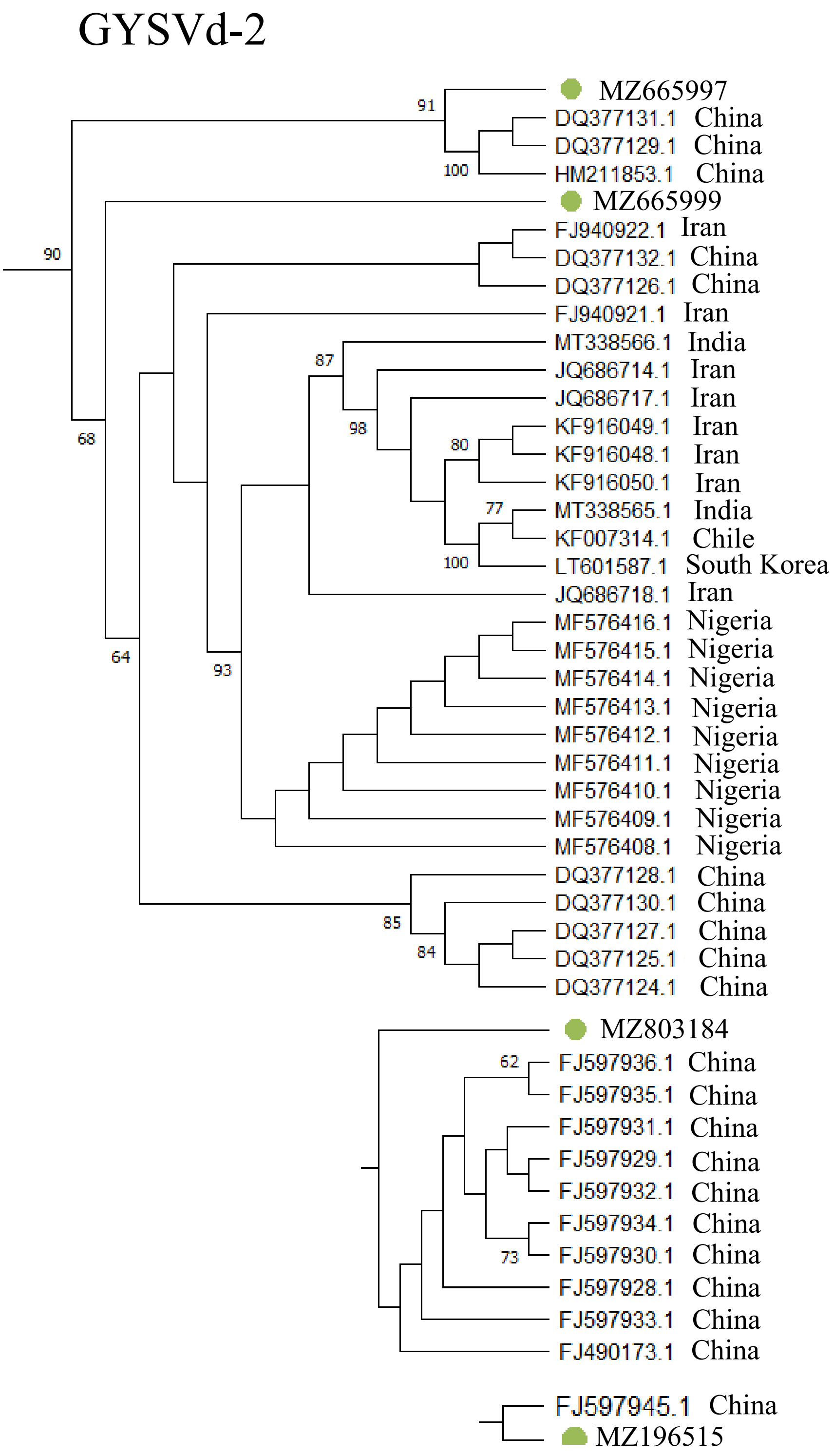

Figure S16. Phylogenetic analysis of global isolates of GYSVd-2 based on based on total nucleotide from NCBI. Sequences previously reported by us and the corresponding sequences available from GenBank are identified by their isolate names, followed by their GenBank accession number and source. The isolates obtained in this study are indicated by 'green mark'. The tree was constructed using the Maximum Likelihood method. The numbers at the nodes indicate bootstrap support (1,000 replicates). Values below 60% are not shown.

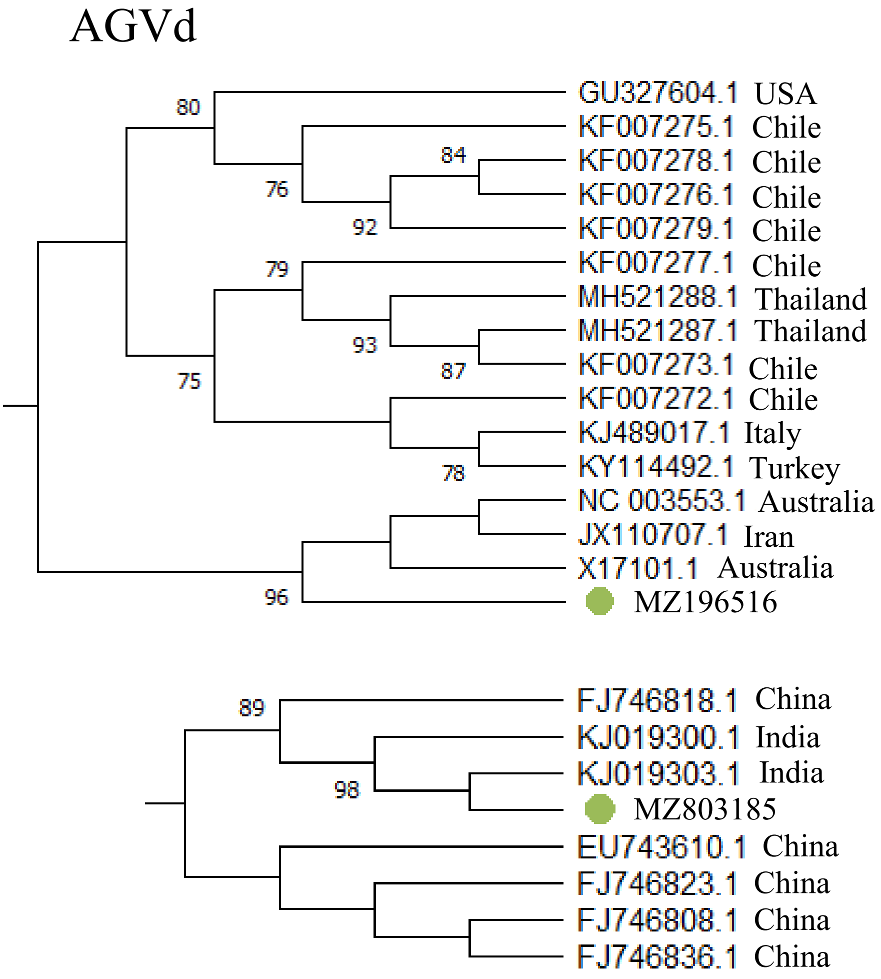

Figure S17. Phylogenetic analysis of global isolates of AGVd based on based on total nucleotide from NCBI. Sequences previously reported by us and the corresponding sequences available from GenBank are identified by their isolate names, followed by their GenBank accession number and source. The isolates obtained in this study are indicated by 'green mark'. The tree was constructed using the Maximum Likelihood method. The numbers at the nodes indicate bootstrap support (1,000 replicates). Values below 60% are not shown.
